# Spatial Transcriptomic Study Reveals Heterogeneous Metabolic Adaptation and a Role of Pericentral PPARα/CAR/Ces2a Axis During Fasting in Mouse Liver

**DOI:** 10.1002/advs.202405240

**Published:** 2024-09-05

**Authors:** Shiguan Wang, Bowen Xu, Jinyuan Liang, Yawei Feng, Penghu Han, Jing Shen, Xinying Li, Mengqi Zheng, Tingguo Zhang, Cuijuan Zhang, Ping Mi, Yi Zhang, Zhiping Liu, Shiyang Li, Detian Yuan

**Affiliations:** ^1^ Department of Biochemistry and Molecular Biology, School of Basic Medical Sciences, Cheeloo College of Medicine Shandong University Jinan 250012 China; ^2^ Department of Clinical Laboratory Qilu Hospital of Shandong University Jinan 250012 China; ^3^ Advanced Medical Research Institute Shandong University Jinan 250012 China; ^4^ Institute of Pathology and Pathophysiology, School of Basic Medical Sciences, Cheeloo College of Medicine Shandong University Jinan Shandong 250012 China; ^5^ Department of Biomedical Engineering, School of Control Science and Engineering Shandong University Jinan Shandong 250061 China

**Keywords:** constitutive androstane receptor (CAR), fasting response, liver zonation, metabolic heterogeneity, peroxisome proliferator‐activated receptor alpha (Pparα), spatial transcriptomics

## Abstract

Spatial heterogeneity and plasticity of the mammalian liver are critical for systemic metabolic homeostasis in response to fluctuating nutritional conditions. Here, a spatially resolved transcriptomic landscape of mouse livers across fed, fasted and refed states using spatial transcriptomics is generated. This approach elucidated dynamic temporal‐spatial gene cascades and how liver zonation—both expression levels and patterns—adapts to shifts in nutritional status. Importantly, the pericentral nuclear receptor Nr1i3 (CAR) as a pivotal regulator of triglyceride metabolism is pinpointed. It is showed that the activation of CAR in the pericentral region is transcriptionally governed by Pparα. During fasting, CAR activation enhances lipolysis by upregulating carboxylesterase 2a, playing a crucial role in maintaining triglyceride homeostasis. These findings lay the foundation for future mechanistic studies of liver metabolic heterogeneity and plasticity in response to nutritional status changes, offering insights into the zonated pathology that emerge during liver disease progression linked to nutritional imbalances.

## Graphical abstract

Spatial transcriptomics reveals how mouse liver zonation adapts to nutritional changes, highlighting the pericentral nuclear receptor CAR as a key regulator of triglyceride metabolism. CAR activation, transcriptionally governed by PPARα, enhances lipolysis during fasting by upregulating carboxylesterase 2a, crucial for maintaining triglyceride homeostasis. This study provides insights into the liver's metabolic heterogeneity and plasticity.

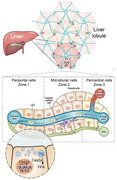

## 1 Introduction

The mammalian liver architecture consists of repeating anatomical and functional units termed lobules, forming a honeycomb‐like pattern.^[^ ^1^, ^2^, ^3^ ^]^ Each lobule is made up of ≈9 to 12 concentric layers of cells with a diameter of ≈0.5 mm. Hepatocytes are arranged between the portal and central veins and exhibits spatial heterogeneity. This zonation is critical for the liver to carry out the broad spectrum of different metabolic tasks simultaneously with great efficiency by the spatial compartmentalization of metabolic pathways.^[^ ^4^ ^]^ For example, periportal hepatocytes, receiving nutrient‐rich blood from the portal vein, and oxygen‐rich blood via the hepatic artery, specialize in gluconeogenesis, ureagenesis, protein secretion and lipid metabolism, whereas pericentral hepatocytes, located in a low oxygen microenvironment, are characterized by bile acid production, xenobiotic biotransformation and glutamine biosynthesis.^[^ ^1^, ^4^ ^]^ Understanding the spatial organization of metabolism and its regulatory mechanisms is vital to broaden our knowledge of liver's complex physiology and the zonated pathology observed during liver disease progression.

Recent advances in single‐cell sequencing technologies have facilitated more in‐depth studies into the cell heterogeneity at a single‐cell level. scRNA‐seq has been applied to reconstruct the spatial mRNA expression profiles along the portal–central axis in the murine liver^[^ ^2^ ^]^ and the human liver,^[^ ^3^, ^5^ ^]^ allowing for dissection of liver zonation at an unprecedented resolution. Beyond characterization of spatial heterogeneity in livers under physiological conditions, a recent study using single‐cell technologies has profiled the transcriptomes of murine hepatocytes during the liver stage of malaria infection, unraveling the effects of hepatocyte zonation on parasite infection at the molecular level.^[^ ^6^ ^]^ Notably, liver zonation is not a static phenomenon, but spatio‐temprorally regulated at the sub‐lobular scale, playing vital roles in maintaining body physiology and energy homeostasis in response to dynamic nutritional signals.^[^ ^7^ ^]^

Hepatocyte gene expression programs are constantly adjusted to changing nutrient availability.^[^ ^8^ ^]^ During fasting, the liver activates glycogenolysis and gluconeogenesis to supply extrahepatic tissues with glucose and restore homeostasis. Prolonged fasting further stimulates the lipolysis of triglyceride (TG) stored in the adipose tissue, liberating free fatty acids (FFAs) and other lipids into the circulation.^[^ ^9^ ^]^ Peripheral FFAs are captured by hepatocytes and directed to fatty acid oxidation (FAO) and ketogenesis, producing soluble ketone bodies that can be used as an energy source in extrahepatic tissues. Coordinated adaptation across different hepatic zones is essential for maintaining proper liver function and overall organismal well‐being. Although the transcriptional regulation of these programs in bulk liver has been intensively studied,^[^ ^10^ ^]^ identifying heterogeneous transcriptional remodeling in response to fasting using spatial transcriptomic approaches is lacking.

Spatial transcriptomics (ST) enables high‐resolution assessment of spatial gene expression across tissue sections.^[^ ^11^ ^]^ By assaying cells in their native tissue context, ST avoids the technical artefact associated with tissue dissociation.^[^ ^12^ ^]^ These advantages make ST a compelling venue to explore the transcriptional and functional compartmentalization in highly heterogeneous tissues including the liver. In this study, we applied ST to analyze livers from mice under varying nutritional conditions and investigated the spatial factors involved in liver heterogenous transcriptional regulation in response to fasting.

## 2 Results

### 2.1 ST Analysis of Murine Livers in Response to Fasting and Refeeding

**Figure 1:**
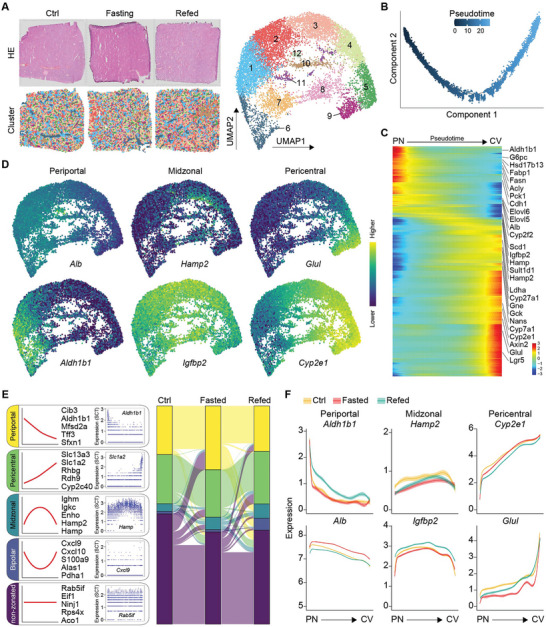
Spatial transcriptomic profiling of the murine livers in fed, fasted and refed conditions. A) Left top: H&E staining of liver sections from ctrl, fasted and refed mice. Right: UMAP visualization of 12 identified clusters. Left bottom: Spatial distribution of each cluster in liver tissue sections captured on 10x Visium slides. B) Pseudotime inference by the DPT algorithm. C) Heatmap displaying mRNA expression of selected genes detected by ST from periportal (PN) to centrilobular (CV) regions. D) Projection of selected marker genes in UMAP space. E) Left, Regression analysis defines five zonation patterns, displaying the standard fit model, top five genes by zonation index, and expression values for a representative gene across pseudotime for each pattern. Right, Sankey diagram depicting zonation pattern transitions across nutritional states. F) Gene expression changes along the PN‐CV axis. Orange, red, and green represent ctrl, fasted, and refed samples. Ribbons within the fitted line represent standard error of gene expression.

To analyze the spatial transcriptomic landscape of the murine liver during fasting, 8‐week‐old male C57BL/6J mice were fasted for 16 h starting at Zeitgeber time 12 (ZT12), followed by 6 h of refeeding. ST was performed on liver sections from the ctrl, fasted, and refed states. Approximately 4000 genes and 24 000 unique molecular identifiers (UMIs) were identified per spot, with comparable results across samples (Figure S1A, Supporting Information). The fasted sample exhibited a higher proportion of mitochondrial transcripts (Figure S1A, Supporting Information), reflecting the increased mitochondrial oxidative function during fasting. Pre‐processing, batch correction and dimensionality reduction revealed 12 spot clusters on the uniform manifold approximation and projection (UMAP) plot (**Figure** **1**A). Among these clusters, six major clusters were located at the outer edge, while six minor clusters were found at the inner side. Marker gene annotation indicated that the inner clusters contained a higher proportion of genes coding the alpha and beta globin chains indicative of red blood cells (Figure S1B, Supporting Information), as expected of the ≈55 µm spatial resolution available when using 10x Visium.

We ordered the 6 major clusters based on diffusion pseudotime (DPT) analysis (Figure 1B), an approach commonly used for reconstructing zonated profiles including human hepatocytes.^[^ ^3^, ^13^, ^14^ ^]^ DPT recovered zonated expression patterns of landmark genes: for example, *Aldh1b1*, *Fasn*, *Pck1*, *Cdh1*, *Alb* and *Cyp2f2* (periportal), *Scd1*, *Igfbp2*, *Hamp*, *Sult1d1* and *Hamp2* (midzonal), and *Gck*, *Cyp2e1*, *Axin2*, *Glul* and *Lgr5* (pericentral) along the pseudo‐time recapitulating the porto‐central lobule axis (Figure 1C). We chose six highly expressed liver genes with different zonation patterns and visualized their expression on UMAP, where they exhibited clear gradients matching their known zonation patterns (Figure 1D). When inspecting the expression of the six reference genes in their spatial context, these genes showed clear zonated pattern with the repeating hexagon‐shaped lobule structure corresponding to the anatomical units (Figure S1C, Supporting Information). These findings complemented previous single‐cell transcriptomics approaches and validated the accuracy of DPT in reconstructing spatial mRNA profiles.^[^ ^3^ ^]^

To complement the DPT analysis, we performed regression analysis on genes detected with over 10 counts in at least 200 spots along pseudotime. We utilized the normalized range of the fitted model for each gene to classify them into five zonation patterns: periportal, pericentral, midzonal, bipolar, and non‐zonated, as depicted in Figure 1E. Analyzing these patterns under three nutritional conditions showed that the majority of genes preserved their zonation patterns (Figure S1D, Supporting Information). For instance, the zonation and expression levels of six landmark genes remained consistent across the conditions (Figure 1F), indicating that the overall spatial organization of hepatocytes largely maintains its integrity through periods of fasting and refeeding. Subsequently, we investigated the effects of fasting on gene expression differences and zonated expression patterns.

### 2.2 Downregulated Metabolic Processes During Fasting Phase

**Figure 2:**
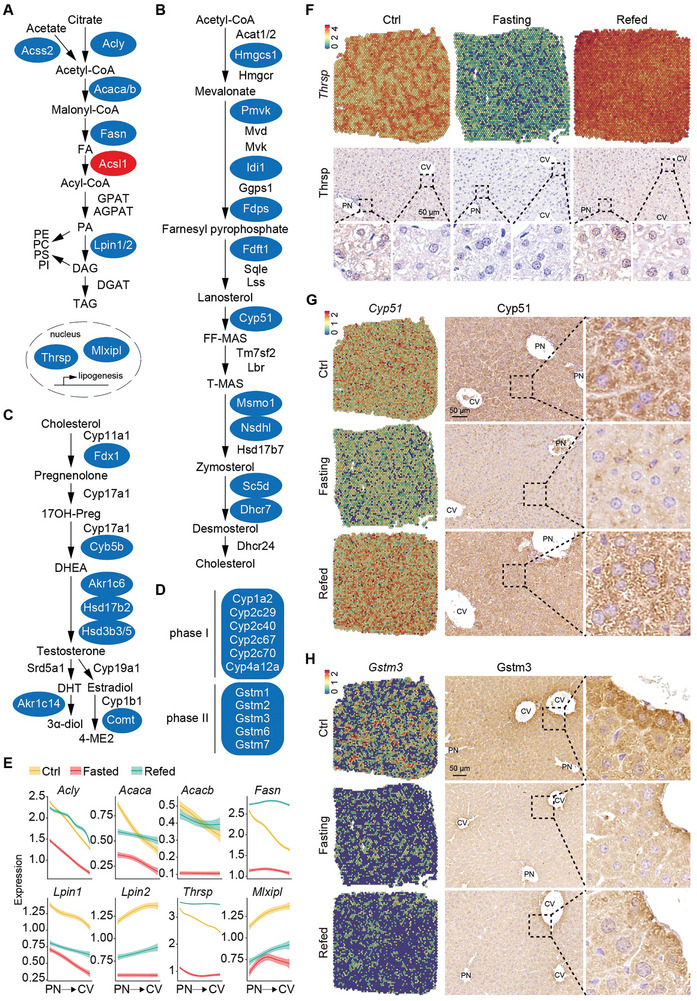
Downregulated metabolic processes revealed by ST during fasting. A–D) Key anabolic processes downregulated by fasting: A) de novo lipogenesis, B) cholesterol biosynthesis, C) steroidogenesis, and D) xenobiotic biotransformation. Blue indicates downregulation, and red indicates upregulation. E) Quantified profiles of the indicated genes along the PN‐CV axis from ST. F–H) Expression of Thrsp F), Cyp51 G) and Gstm3 H) visualized in tissue spots and detected by IHC in liver sections. The CV and PN are marked.

To select a reliable set of mRNA profiles for subsequent analyses, we defined differentially expressed genes (DEGs) for each of the 6 major clusters at an expression threshold of logFC > 0.25 and FDR < 0.05, identifying 185 and 714 genes in total that were differentially up‐ or downregulated, respectively (Figure S2A, Tables S1 and S2, Supporting Information). Functional annotation of the DE genes and Gene Set Enrichment Analysis (GSEA) revealed that the top pathways downregulated by fasting were anabolic processes (Figure S2B,C, Supporting Information), including *de novo* lipogenesis (**Figure** **2**A), cholesterol biosynthesis (Figure 2B) and steroidogenesis (Figure 2C), and xenobiotic biotransformation (Figure 2D). In contrast, comparing the refeeding state to the fasting state showed a marked increase in these anabolic processes (Figure S3A–C, Supporting Information), consistent with the physiological response to nutrient reintroduction and metabolic restoration.

All the three rate‐limiting enzymes in *de novo* lipogenesis, ATP‐citrate lyase (*Acly*), acetyl‐CoA carboxylase (encoded by *Acaca* and *Acacb* in mouse) and fatty acid synthase (*Fasn*), were portally biased and downregulated during overnight fasting (Figure 2A,E). Additionally, expression of all these enzymes were restored upon refeeding with the portally biased pattern, except for *Fasn*, whose expression became non‐zonated after refeeding (Figure 2A,E; Figure S4A, Supporting Information). Thyroid hormone responsive (*Thrsp*), a key lipogenic transcription factor, showed the same pattern with *Fasn*: enriched in periportal hepatocytes, downregulated by fasting and universally expressed after refeeding (Figure 2A,E). The dynamic expression of Thrsp was verified by immunohistochemistry (IHC) quantitative real‐time PCR (Figure 2F) and qRT‐PCR (Figure S4D, Supporting Information). These results were in line with the previous reports that periportal hepatocytes specialize with lipogenesis, and also suggested that the whole liver may be mobilized for *de novo* lipogenesis upon fasting/refeeding stimulation.

The majority of genes involved in cholesterol biosynthesis were reduced during fasting period and upregulated after refeeding, including *Hmgcs1*, the key enzyme in the mevalonate pathway, and *Cyp51*, the initial checkpoint in the transformation of lanosterol to other sterols (Figure 2B; Figure S4A,B, Supporting Information). Notably, other expression patterns were also seen (Figure S4A and D, Supporting Information). For instance, although *Hmgcr* and *Dhcr24* were substantially induced by refeeding, their mRNA levels were largely unaltered in response to fasting. Expression of *Lbr* and *Ggps1* remained almost steady across the three conditions. *Acat1* and *Tm7sf2* was not restored or even downregulated after refeeding. Contrary to the lipogenic genes that were predominantly periportal biased, genes involved steroid biosynthesis were weakly zonated under fed state (Figure S4A, Supporting Information). Expression alterations corresponding to food availability of Cyp51 were verified by IHC, showing non‐zonated distribution (Figure 2G). Notably, most of these cholesterol biosynthesis‐related genes were upregulated with a portal‐biased pattern after refeeding (Figure S4A, Supporting Information). This alteration in the zonation profiles could suggest an adaption to substrate availability, since periportal blood entering the lobule is rich in nutrients after refeeding. Cholesterol serves as the building block for synthesizing various steroid hormones such as testosterone, estrogen, progesterone, cortisol and aldosterone. Consistent with the decline of cholesterol biosynthesis, steroid hormone biosynthesis was also turned down during fasting phase, including ferredoxin 1 (Fdx1) (Figure 2C; Figures S3B and S4D, Supporting Information), which controls steroid hormone biosynthesis through electron transfer to *Cyp11a1*, the first and rate‐limiting steroidogenic enzyme.^[^ ^15^ ^]^

Xenobiotic metabolism was known to be pericentrally localized. We found that many enzymes involved in the biotransformation of xenobiotics via hepatic oxidative (phase I) and conjugative (phase II) pathways were dramatically downregulated during fasting period (Figure 2D; Figure S4C,D, Supporting Information). Expression dynamics of Glutathione S‐transferase mu 3 (*GSTM3*) in response to fasting and fasting/refeeding were validated using IHC (Figure 2H) and qRT‐PCR (Figure S4D, Supporting Information). This feature is line with the fact that fasting and nutritional deficiencies are associated with lower levels of xenobiotic enzymes and lowered rates of xenobiotic metabolism.^[^ ^16^ ^]^

### 2.3 Upregulated Cellular Processes During Fasting Phase

We next investigated the upregulated genes during the fasting stage (Figure S2B, Supporting Information). Chaperones has been recently shown to be zoned expressed and regulated rhythmically in the liver, with the cytosolic chaperones accumulating centrally and the endoplasmic reticulum (ER) chaperons portally biased.^[^ ^7^ ^]^ In accordance with the reported expression pattern that both groups of chaperones peak during the feeding phase, we found that several chaperons like *Hsp90b1*, *Dnajc3*, *Hsp90aa1* were strongly induced after refeeding (Figure S5A–C, Supporting Information). By contrast, several other chaperons including *Hspa5*, *Manf*, *Hsp90ab1*, *Dnajb9*, *Hspa1b*, *Hsph1*, *Hspb8*, *Bag3*, and *Hspa8* displayed the highest mRNA levels during the phase of overnight fasting (Figure S5A–C, Supporting Information), with the dynamics of Hspa5 expression validated by qRT‐PCR and IHC (Figure S5C,D, Supporting Information). We speculated that this discordance may reflect the differences between normal fasting/feeding cycles and overnight fasting, as the fasting duration has an obvious impact on metabolic adaption.^[^ ^8^, ^17^ ^]^ While the need for protein folding capacity is enhanced after feeding, overnight fasting may activate integrated stress response (ISR) due to insufficient amino acid supply. This interpretation is supported by the upregulation of two transcription factors, *Atf4* and *Ddit3*, known to counteract amino acid deficiency within the ISR pathway,^[^ ^18^, ^19^, ^20^ ^]^ were significantly upregulated upon overnight fasting (Figure S4E and F, Supporting Information).

**Figure 3:**
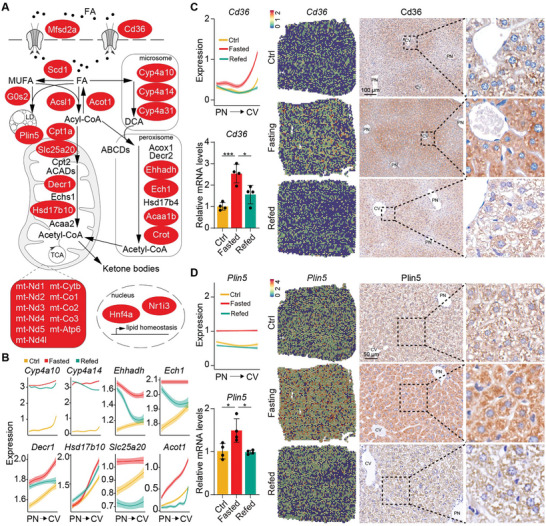
Enhancement of fatty acid oxidation pathways, including mitochondrial and peroxisomal β‐oxidation and microsomal ω‐oxidation, during fasting. A) Upregulated genes involved in the process of lipid metabolism during fasting are highlighted in red. B) Quantified profiles of the indicated genes along the PN‐CV axis from ST. C,D) Quantified profiles of *Cd36* C) and *Plin5* D) along the PN‐CV axis from ST, accompanied by corresponding qRT‐PCR analysis, visualization in tissue spots, and IHC staining in liver sections.n = 4 mice for each group.Data are shown in mean ± SEM; ns, not significant, *p < 0.05, **p < 0.01, ****p < 0.0001 by Student's t test.

As expected, FAO, including mitochondrial and peroxisomal β‐oxidation and microsomal ω‐oxidation, was strongly enhanced upon prolonged fasting (**Figure** **3**A–C; Figure S6A,B, Supporting Information). In line with the well‐documented centrally biased lipid metabolism, the majority of genes involved in FAO were enriched in the pericentral layers under fed state (Figure 3B; Figure S6A, Supporting Information). Importantly, the FAO‐related genes were strongly enhanced during the phase of fasting to the extent that the entire lobules were expressing these enzymes, forming non‐zonated pattern (Figure 3B). This phenomenon suggested that the whole liver lobules may most likely be reprogramed for FAO and ketogenesis to maintain energy homeostasis in response to food deficiency.

Other processes of lipid metabolism such as lipid abortion, lipid droplet (LD) formation and mobilization, as well as transcription factor *Hnf4a* ^[^ ^21^, ^22^ ^]^ and *Nr1i3*, were also reprogramed during fasting (Figure 3A). For instance, Cd36, a pericentral fatty acid translocase, was upregulated in response to fasting, in accordance with the higher needs for lipid uptake and utilization (Figure 3A). We verified the zonated and dynamic expression of Cd36 by qRT‐PCR and IHC (Figure 3C). *Plin5*, a lipid droplet (LD)‐associated gene keeping the balance between lipolysis and lipogenesis, was expressed in a non‐zonated manner. Expression of Plin5 was specifically upregulated in fasted liver,^[^ ^23^ ^]^ as also verified by quantitative real‐time PCR (qRT‐PCR) and IHC (Figure 3D). Uncoupling LD accumulation from lipo‐toxicity is very important for the liver under fasting conditions, as fasted liver is featured by intrahepatic TG accumulation due to influx of fatty acids from peripheral blood, despite high rates of FAO.^[^ ^24^, ^25^ ^]^ Indeed, it was recently reported that Plin5 deficiency triggered a pro‐inflammatory response in livers from mice only under fasting conditions,^[^ ^26^ ^]^ highlighting the importance of proper lipid storage and handling in promoting health.

### 2.4 PPARα‐Dependent CAR Induction In the Pericentral Region of the Liver in Response to Fasting

**Figure 4:**
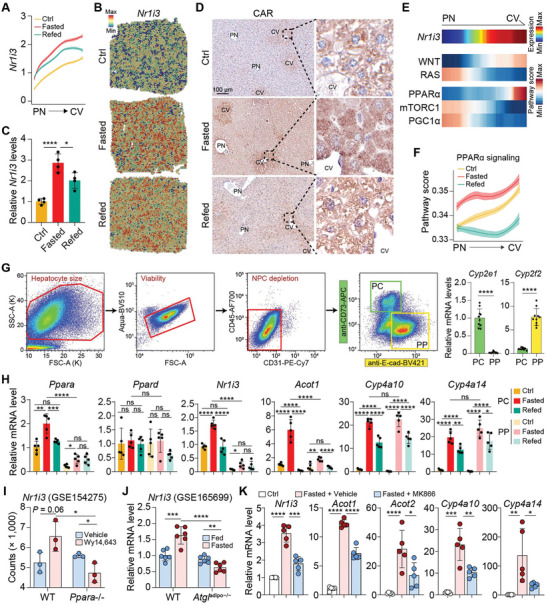
Fasting‐induced upregulation of CAR is associated with PPARα in the pericentral zone of liver lobules. A) Quantified profiles of Nr1i3 (encoding CAR) along the PN‐CV axis from ST. B) Visualization of Nr1i3 in tissue spots. C) mRNA levels of Nr1i3 in liver samples obtained from the ctrl, fasted and refed mice. n = 4 mice for each group. D) Representative IHC staining of Nr1i3 in the liver sections. E) Heatmap depicting the expression of Nr1i3, and pathway scores determined by ssGSEA for the indicated signaling pathway. F) Scores of PPARα signaling along the PN‐CV axis. G) Sorting strategy for PC and PP hepatocytes, accompanied by qRT‐PCR validation of Cyp2e1 and Cyp2f2. n = 10 mice for each group. H) mRNA levels of Nr1i3 and the genes related to PPARα signaling in PP and PC hepatocytes obtained from ctrl, fasted and refed mice. n = 5 mice for each group. I) Expression of Nr1i3 in livers from WT and Ppara knockout mice upon Wy14643 treatment. n = 3. J) Expression of Nr1i3 in livers from WT and Atgladipo‐/− mice upon fasting. n = 6. K) mRNA levels of the indicated genes in liver samples obtained from the ctrl or fasted mice treated with Vehicle or MK866. n = 5 mice for each group. Data are shown in mean ± SEM; ns, not significant, *p < 0.05, **p < 0.01, ***p < 0.001 by Student's t test.

Among the fasting‐responsive genes displaying zonational patterns, we observed that Nr1i3 (commonly known as CAR) is upregulated in a pericentral zone‐biased manner upon fasting (**Figure** **4**A,B). CAR, a member of the nuclear receptor superfamily, is a key regulator of xenobiotic and endobiotic metabolism.^[^ ^27^, ^28^ ^]^ Recent studies have implicated CAR in energy metabolism: it is upregulated during fasting;^[^ ^29^, ^30^ ^]^ specific CAR agonist TCPOBOP treatment significantly improves fatty liver histology and decreases hepatic TG in a mouse model of NAFLD;^[^ ^31^ ^]^ reduced hepatic lipid content by CAR activation was reported to be indirectly linked with the suppression of LXR signaling and the promotion of FAO.^[^ ^31^ ^]^ Importantly, the zonational expression of CAR within the liver, particularly in the pericentral zone, and its downstream targets responsible for these metabolic effects had not been thoroughly explored prior to our study. Given CAR's significant potential as a therapeutic target for conditions like NAFLD, we next focused on CAR to uncover its precise role in fasting‐induced metabolic adaptations.

We confirmed the elevated expression of CAR in response to fasting through qRT‐PCR and IHC, with IHC further validating its enrichment in the pericentral zone (Figure 4C,D). Peroxisome proliferator‐activated receptor α (PPARα) is recognized as the master regulator of lipid metabolism for the adaptive response to fasting.^[^ ^32^, ^33^, ^34^ ^]^ It is accepted that fatty acids derived from adipocyte lipolysis control PPARα activity in the liver.^[^ ^35^, ^36^ ^]^ Recent single‐cell RNAseq and proteomics study of hepatocytes revealed preferential gene expression in pericentral areas of *Ppara* and of its targets.^[^ ^2^, ^7^, ^37^ ^]^ Indeed, the elevation of the signaling pathway activity using the ssGSEA algorithm indicated that, among the three nutrition‐sensing pathways analyzed (PPARα, mTORC1, and PGC1α), the PPARα signaling pathway exhibited a pericentral bias, akin to the WNT pathway and in contrast to the periportal‐biased RAS pathway, both known for their roles in liver zonation (Figure 4E). Additionally, the PPARα pathway score increased in response to fasting (Figure 4F). To validate the zonated expression of PPARα signaling and CAR under different nutritional conditions, we employed a newly reported FACS‐based method to sort pericentral and periportal hepatocytes^[^ ^38^ ^]^ (Figure 4G), and found that *Ppara* and its known targets *Acot1*, but not *Ppard*, were more strongly upregulated in pericentral hepatocytes, while *Cyp4a10* and *Cyp4a14* were equally induced in both pericentral and periportal hepatocytes (Figure 4H), in aligning with ST findings (Figure 3D). Notably, *Nr1i3* expression closely mirrored *Ppara* (Figure 4H), positioning CAR within the Pparα network. Double immunofluorescence staining demonstrated the co‐localization of CAR and PPARα, accompanied by their upregulation specifically within the pericentral zone in response to fasting (Figure S7A, Supporting Information). Reanalysis of publicly available datasets revealed the Pparα‐dependent nature of Wy14643‐induced *Nr1i3* expression, as it was abolished in *Ppara* knockout mice (Figure 4I).^[^ ^39^ ^]^ Furthermore, fasting induced PPARα activity in hepatocytes relies on circulating free fatty acids released from adipocytes via adipose triglyceride lipase (ATGL).^[^ ^36^ ^]^ Mice with adipocyte‐specific *Atgl* deletion (*Atgl* ^adipo‐/−^) exhibited no increase in *Nr1i3* expression in the liver upon fasting (Figure 4J).^[^ ^36^ ^]^ Using an in vitro fasting model^[^ ^40^, ^41^, ^42^ ^]^ we observed that siRNA‐mediated knockdown of Pparα in AML12 cells significantly reduced the fasting‐induced upregulation of CAR (Figure S7B–D, Supporting Information). This dependency on Pparα for Nr1i3 induction was further corroborated by the diminished *Nr1i3* upregulation in fasted mice treated with Pparα inhibitor MK866, alongside other Pparα targets (Figure 4K).

**Figure 5:**
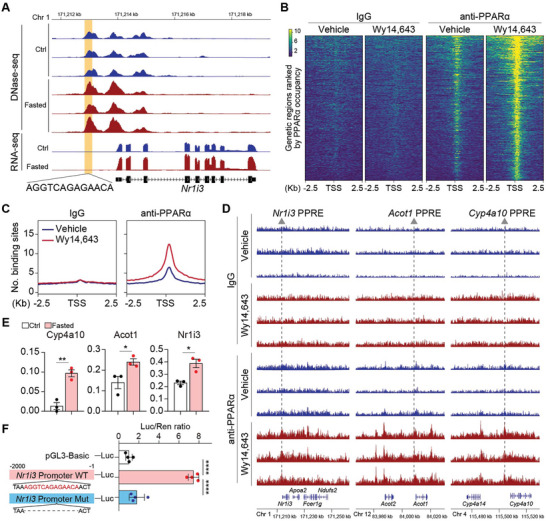
PPARα regulates CAR expression via PPRE binding in its promoter. A) DNase‐seq data illustrating the peak profiles at the Nr1i3 promoter region in the livers of ctrl and fasted mice, with the PPRE motif highlighted. B) Heatmaps displaying the ChIP‐seq signals. C) Profile plots representing the average ChIP signals. D) PPARα‐binding peaks were visualized using IGV software. E) ChIP‐qPCR validation of PPARα binding to the PPRE within the promoters of the indicated genes. n = 3 mice per group. F) Luciferase reporter assays in 293T cells transfected with pGL3 luciferase reporter plasmids the Nr1i3 promoter region (−2000 to ‐1 bp) and a variant lacking the PPRE motif. n = 4. Data are shown in mean ± SEM; ns, not significant, *p < 0.05, **p < 0.01, ****p < 0.0001 by Student's t test.

To further elucidate the in vivo activation mechanism of CAR by Pparα, we analyzed a DNase‐seq dataset comparing livers from fasted versus fed mice.^[^ ^39^ ^]^ Enhanced signal intensity was observed roughly 1 kb upstream of the Nr1i3 transcription start site, indicating increased chromatin accessibility upon fasting (**Figure** **5**A). Notably, this region harbors the DR1 motif, previously validated as necessary and sufficient for CAR induction in vitro.^[^ ^30^, ^43^ ^]^ Consistently, a reevaluation of ChIP‐seq data confirmed increased PPARα binding in response to Wy14643 treatment (Figure 5B,C), particularly at the Nr1i3 PPRE site and at loci of known PPARα targets, including Acot1/2, Cyp4a10 and Cyp4a14 (Figure 5D). ChIP‐qPCR further corroborated increased PPARα binding to Nr1i3's PPRE in fasted livers, alongside Acot1 and Cyp4a10 as positive controls (Figure 5E). Additionally, luciferase reporter assays with *Nr1i3* promoter constructs—both full‐length and a variant lacking the PPRE—underscored the necessity of the intact PPRE for Wy14643‐induced transcriptional activity (Figure 5F). Together with the results above, these data support that CAR expression is transcriptionally upregulated in the liver's pericentral region during fasting via a Pparα‐mediated mechanism.

### 2.5 Role of Pericentral CAR/Ces2a Axis in TG homeostasis During Fasting

**Figure 6:**
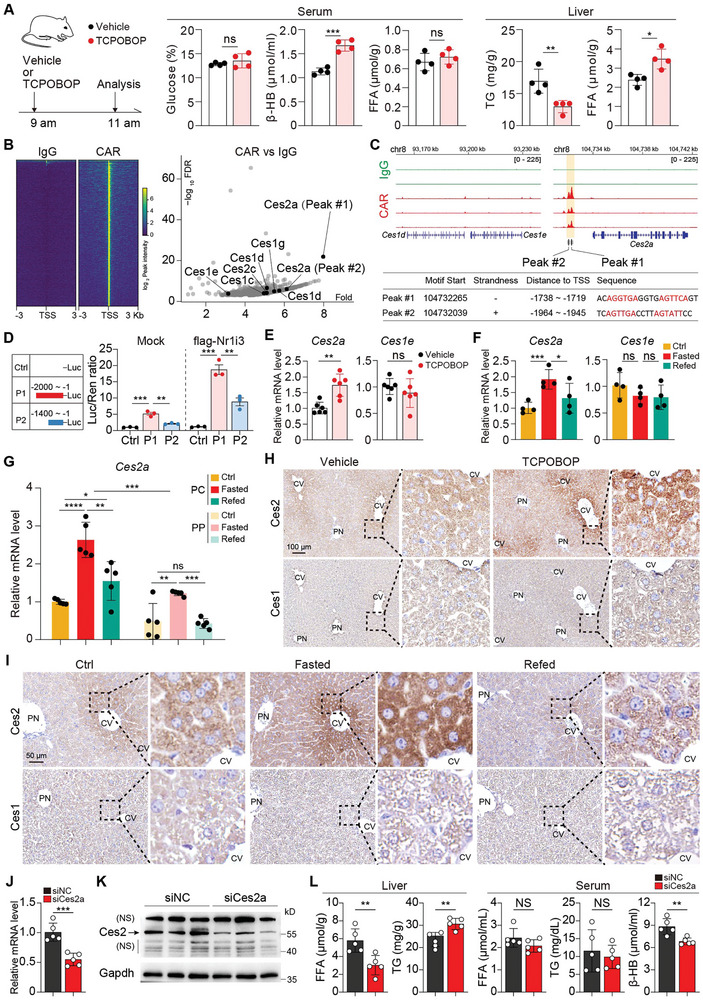
Ces2a, downstream of CAR, maintains TG homeostasis during fasting. A) Experimental setup for TCPOBOP treatment and analysis of serum β‐BHB, glucose, FFA and liver TG and FFA levels. n = 4 mice per group. B) Left: ChIP‐seq heatmaps showing CAR and IgG signals in mouse livers. Right: Quantification of CAR versus IgG signals. Peaks falling within promoter regions of Ces family genes are indicated. C) Genome browser tracks of CAR and IgG at the indicated Ces family gene promoters, featuring the canonical CAR‐binding site in the Ces2a promoter. D) Schematic of Ces2a promoters (P1/P2: with/without CAR‐binding region) and dual‐luciferase reporter assay. n = 3 per group. E) qRT‐PCR analysis of Ces2a and Ces1e in the liver samples treated with Vehicle or TCPOBOP. n = 6 mice per group. F) mRNA levels of the Ces1e and Ces2a in liver samples obtained from the ctrl, fasted and refed mice. n = 4 mice per group. G) Expression of Ces2a in PP and PC hepatocytes obtained from ctrl, fasted and refed mice. n = 5 mice per group. H) IHC of Ces1 and Ces2 in vehicle‐ or TCPOBOP‐treated livers. I) IHC of Ces2 and Ces1 in fed, fasted and refed livers. J,K) Silencing efficiency of Ces2a‐targeting siRNA assessed by qRT‐PCR J) and immunoblotting K) in mouse livers. n = 5 mice per group. H) Effects of Ces2a knockdown on liver FFA and TG contents and serum levels of TG and β‐HB in mice upon fasting. n = 5 mice per group. Data are shown in mean ± SEM; ns, not significant, **p < 0.01 by Student's t test.

We next investigated the role of pericentral CAR activation in response to fasting. Consistent with the previous report mentioned above, TCPOBOP treatment for 2 h resulted in reduction of liver TG levels and elevated levels of liver FFA and serum β‐BHB, while the plasma levels of glucose and FFA were unaffected (**Figure** **6**A). However, we did not find substantial effects of CAR activation on gene expression of key components of FAO and ketogenesis pathways (Figure S8A, Supporting Information), which was also in line with the previous observations.^[^ ^31^ ^]^ Although CAR has been shown to directly activate insulin‐induced gene‐1 (Insig1), an ER‐bound cholesterol sensor with anti‐lipogenic properties, in response to xenobiotics,^[^ ^44^ ^]^ expression of *Insig1* was downregulated during fasting phase and strongly induced after refeeding, a pattern opposite to that of CAR (Figure S8B, Supporting Information). Thus, the specific targets by which CAR contributes to fasting adaptation remain to be elucidated.

To discern CAR's downstream effector facilitating its fasting response, we analyzed a publicly available ChIP‐seq dataset performed with livers from TCPOBOP‐treated mice.^[^ ^45^ ^]^ We noticed that, among the peaks occupied by CAR, two binding sites located in the promoter region of carboxylesterase 2a (Ces2a), but not in the promoter regions of other family members (Figure 6B; Figure S9A, Supporting Information). Further examination revealed canonical CAR‐binding sites consisting of direct repeat 4 (DR4) units within both peaks^[^ ^46^ ^]^ (Figure 6C).

CES was initially categorized as serine esterase, playing crucial roles in both endo‐ and xenobiotic metabolism.^[^ ^47^ ^]^ It was subsequently recognized that CESs have TG hydrolase activity and regulates hepatic TG levels by modulating lipolysis.^[^ ^48^, ^49^ ^]^ Expression of CES2 is reduced in livers of NAFLD patients as well as in the pre‐clinical mouse models of NAFLD, highlighting the importance of lipolysis in hepatic TG homeostasis.^[^ ^49^ ^]^ The reported function of Ces2 in promoting TG hydrolase may explain the role of CAR in hepatic TG homeostasis.^[^ ^49^ ^]^ To test this hypothesis, we first verified the effect of CAR on Ces2a transcription by dual‐luciferase reporter assay, showing that *Nr1i3* significantly induced luciferase activity driven by the full‐length *Ces2a* promoter (P1) but not the truncated promoter without the CAR‐binding sites (P2) (Figure 6D). Consistently, transcripts of *Ces2a*, but not the other Ces superfamily members, was upregulated by TCPOBOP treatment (Figure 6E; Figure S9B, Supporting Information). Similarly, *Ces2a* was upregulated in livers upon fasting (Figure 6F). qPCR analysis of sorted hepatocytes further revealed a pericentral enrichment of *Ces2a* expression during fasting (Figure 6G). IHC further confirmed that Ces2 was predominantly peri‐central, and was responsive to TCPOBOP and fasting, while Ces1 protein was non‐zonated and did not fluctuate across these conditions (Figure 6H,I; Figure S9C, Supporting Information). These results indicated that Ces2a may function as a downstream target of CAR, paralleling CAR's spatiotemporal expression pattern in the fasting response.

To test whether the CAR/Ces2a axis was functionally linked to hepatic TG metabolism, we transfected cationic liposome‐encapsulated short interfering RNA (siRNA) targeting mouse Ces2a (siCes2a) into AML12 cells and screened for highly efficient siRNA (Figure S10A,B, Supporting Information). CAR (Nr1i3) overexpression in AML12 cells resulted in reduced TG levels, an effect that was significantly inhibited by Ces2a knockdown (Figure S10C,D, Supporting Information). In vivo, we administered siCes2a via tail vein injection, with a non‐targeting siRNA serving as the control (siNC). We confirmed efficient Ces2a knockdown via qRT‐PCR, immunoblotting and IHC (Figure 6J, K; Figure S10E, Supporting Information). Notably, we found that Ces2a knockdown exacerbated liver TG accumulation and reduced levels of liver FFA and serum β‐BHB in response to fasting, while serum levels of TG, FFA, and glucose were largely unaffected (Figure 6L; Figure S10F, Supporting Information). These findings underscore the role of Ces2a as a pericentral TG hydrolase governed by CAR, essential for modulating hepatic TG homeostasis during fasting.

## 3 Discussion

Here, we used spatially resolved approach to systemically uncover transcriptomic changes in the mouse livers in response to fasting and fasting/refeeding. ST preserves the spatial information of the gene expression in its native tissue context, avoiding tissue dissociation and the resultant undesirable transcriptional perturbations. These advantages make ST a compelling venue to explore the transcriptional and functional compartmentalization in highly heterogeneous tissues including the liver. In addition, previous scRNA‐seq studies used ab initio methods like diffusion pseudotime to infer the zonation coordinate.^[^ ^3^ ^]^ Our data showed that such computational methods can reconstruct the spatial mRNA profiles with high fidelity, thus complementing the previous single‐cell transcriptomics approaches.

We generated a spatially resolved transcriptional atlas of the liver associated with prolonged fasting and fasting/refeeding. Although the gross spatial organization of hepatocytes was largely stable during fasting and fasting/refeeding, we observed notable spatially dependent changes in gene expression at the molecular level. The spatially resolved profiles of the corresponding genes allowed us to better understand the identified metabolic processes. For instance, portally biased *de novo* lipogenesis was turned down upon fasting. While the majority of the enzymes were restored upon refeeding with the same portally biased pattern, the lipogenic transcription factor Thrsp and the key enzyme Fasn were strongly induced and adopted non‐zonated pattern. Similarly, while the majority of genes involved in FAO were enriched in the peri‐central layers under fed state, their expression was strongly enhanced during the phase of prolonged fasting and extended across the entire lobules. These observations support the notion that high transcriptional plasticity of hepatocytes ensures the metabolic plasticity, with the zonated liver functions reprogramed to optimize the diverse liver tasks in response to the changing nutrition status.

Our results from ST also complement a recent scRNA‐seq study of mouse hepatocytes at different times of the day by Droin et al., in which the authors characterized both zonation and chronobiology in the liver during the daily feeding/fasting cycles at single‐cell resolution.^[^ ^7^ ^]^ For example, both the ST data and the scRNA‐seq data showed that expression of chaperones is zonated in the liver. Notably, some quantitative differences were also observed. Droin et al. showed that chaperones peak during the activity/feeding phase, likely reflecting increased needs of protein folding during times of high protein synthesis and secretion. However, our ST data and IHC analysis showed that chaperones is substantially upregulated in livers fasted for 18 h. This discordance may reflect the differences between the daily feeding/fasting cycles and the prolonged fasting, the latter of which may lead to higher protein stress due to deficiency of dietary protein that drives ISR. This interpretation is corroborated by the observed increased levels of Atf4 and Ddit3 upon prolonged fasting, counteracting amino acid limitation.

The liver is critical for coordinating catabolic and anabolic metabolism during periods of starvation in mammals. Although the enzymes involved in liver lipid metabolism have been well defined, the regulatory mechanisms orchestrating its homeostasis in diverse physiological states remain incompletely characterized. As an important feature of starvation response, the marked influx of fatty acids leads to intrahepatic triglyceride accumulation (steatosis) despite high rates of fatty acid oxidation. The importance of maintaining liver lipid homeostasis is highlighted by widespread diet‐influenced pathologies such as non‐alcoholic fatty liver disease and other metabolic syndromes. Notably, a previous study in humans demonstrated that peripheral FFA during the fasting is the major source of liver TG accumulation in patients with NAFLD.^[^ ^50^ ^]^ In our study, we identified Ces2a as a CAR‐regulated CES enzyme responsible for increased hepatic lipolysis activity during fasting. Whereas previous studies have reported the role of CAR and CESs in liver metabolic modulation, this is to our knowledge the first report of zonation of CAR and Ces2a in the liver. Our results indicate that induction of Ces2a by CAR impact on the balance between intrahepatic TG accumulation and mobilization upon fasting. Intriguingly, contrary to Ces2, expression of Ces1 remains constant across the conditions explored in this study. We propose that Ces1 functions as the constitutive CES while Ces2 acts as the inducible CES in the liver, meeting the fluctuated requirement of the CES superfamily's enzymatic activity. Future studies utilizing liver‐specific knockout models for multiple Ces isoforms may provide further insights into their roles in different physiological and pathological processes. Additionally, resmetirom (MGL‐3196), a selective agonist of the thyroid hormone receptor β that also belongs to the nuclear receptor superfamily I, shows promise as a potential therapy for NASH patients with liver fibrosis.^[^ ^51^ ^]^ Elucidating the mechanism governing the regulation of Ces2 expression by CAR may inform novel therapeutic strategies for metabolic disorders characterized by TAG accumulation.^[^ ^52^ ^]^

One of the major limitations of this study is the inability to distinguish between hepatocytes and non‐parenchymal cells. The liver lobule exhibits immunological zonation.^[^ ^53^ ^]^ The increased resolution in the spatial genomics field will promote detailed investigations of the contribution of intercellular crosstalk in starvation adaption.^[^ ^8^ ^]^ Furthermore, the study's observations in C57BL/6J mice may not fully apply to humans due to evolutionary differences in hepatocyte zonation.^[^ ^3^ ^]^ Finally, transcript levels may not accurately reflect protein levels due to protein synthesis and half‐lives. Droin et al. observed a delay of maximally ≈6 h between protein and mRNA rhythms in the liver.^[^ ^7^ ^]^ The emerging possibilities of combining ST with spatial proteomics approaches offers potential for a detailed liver proteomic atlas and deeper insights into liver zonation biology.^[^ ^38^ ^]^

In summary, we generated a spatially resolved transcriptional atlas of the liver associated with overnight fasting and fasting/refeeding. The spatially dependent changes in gene expression supported the notion that high transcriptional plasticity of hepatocytes ensures the metabolic plasticity, with the zonated liver functions reprogramed to optimize the diverse liver tasks in response to the changing nutrition status. Given that liver zonation can give rise to zonated patterns of liver pathologies, advancing our understanding of metabolic compartmentalization will increase both the accuracy and efficacy of future treatments. This study revealed the advantages of ST in both qualitative and quantitative comparisons of gene expression in highly zonated tissue. We anticipate that ST is highly applicable for future studies addressing the zonated pathology during disease progression in the mammalian liver as well as other zonated tissues.

## 4 Experimental Section

### Ethical Statement

The mice were bred and maintained under specific‐pathogen‐free conditions with a 12 h night/day cycle and an ambient temperature of 22 °C. Eight‐week‐old male C57BL/6J mice underwent a fasting protocol, which started at Zeitgeber time 12 (ZT12), the beginning of their active phase, and lasted for 16 h. They were then refed for 6 h. All animal experiments were conducted in accordance with the guidelines provided by the National Institutes of Health Guide for the Care and Use of Laboratory Animals. The study protocol was approved by the Institutional Animal Care and Use Committee of the School of Basic Medical Sciences, Shandong University (Document No. ECSBMSSDU2022‐2‐141).

### Library Preparation and Sequencing

Liver tissues were carefully sliced into small pieces measuring ≈6.5 × 6.5 × 5 mm. These tissue fragments were then embedded in optimal cutting temperature (OCT) embedding solution and promptly frozen in isopentane chilled with liquid nitrogen. Next, ten‐micrometer cryosections were affixed onto the spatial transcriptomic array or Visium tissue optimization slide (10× Genomics Visium, Pleasanton, CA, USA). The sections were then fixed with methanol and subjected to H&E staining according to the Visium staining user guide. Brightfield images of the stained sections were acquired at 20× resolution using an Olympus VS120 slide scanner (Olympus, Tokyo, Japan). Following the imaging step, the cryosections were permeabilized for 18 min, as determined through initial optimization trials conducted with the Visium Spatial Tissue Optimization Kit (10× Genomics). The RNA integrity number (RIN) of the extracted RNA was assessed using Bioanalyzer High Sensitivity RNA Analysis (Agilent). After permeabilization, an on‐slide reverse transcription (RT) reaction was carried out at 53 °C for 45 min. Subsequently, the library was prepared according to the instructions provided in the Visium Spatial Gene Expression User Guide. The library preparation included 16 cycles of PCR for cDNA amplification and an additional 12 cycles for sample index PCR, utilizing the Dual Index Kit TT Set A (10× Genomics, PN‐1000215). Library quality was assayed using a Bioanalyzer High Sensitivity chip (Agilent). The prepared library was sequenced on an Illumina NovaSeq 6000 platform (San Diego, CA, USA).

### Data processing, Data Integration, and Cluster Annotation

The ST sequencing reads were aligned to the mm10 reference genome using Cell Ranger Single Cell v.2.0.1 software from 10x Genomics. The ST data was processed using Seurat version 3.1.4, and normalization was performed using SCTransform. To integrate the three ST datasets, the Seurat SCTransform integration workflow was applied, utilizing 3000 integration features and including all common genes across the three datasets. Principal component analysis and UMAP dimensionality reduction were conducted using default parameters. Initial clustering was performed using the FindClusters function implemented in the Seurat R package, with the resolution parameter set to 0.8. The average log2 fold change for each gene in the clusters was calculated using the Seurat function FindMarkers and the Wilcoxon rank sum test.

### Differential Gene Expression and Pathway Enrichment

Differential gene expression analysis was conducted using the limma package. For each cluster, differentially expressed genes (DEGs) were identified based on a log fold change threshold of >0.5 and a significance threshold of 0.05. The ClusterProfiler package v.3.14.0 was utilized for gene ontology functional enrichment analysis, using the identified DEGs as input. The top 10 significantly enriched processes were visualized using ggplot2 v.3.3.2. To further explore variations in biological process activity, Gene Set Enrichment Analysis (GSEA) was conducted using the fgsea R package v.1.26.0. This analysis utilized gene sets from the Molecular Signatures Database (MSigDB) to identify significant changes in biological processes across experimental conditions.

### Diffusion Pseudotime (DPT) Analysis for Inferring Central to Portal Trajectories

To infer central to portal trajectories, pseudotemporal ordering was performed using Monocle 2 v.2.10.1 with default parameters. Integrated gene expression matrix was exported from Seurat v.3 into Monocle to construct a CellDataSet. The setOrderingFilter function was used to filter the cell ordering based on all variable genes identified by the differentialGeneTest function with a cutoff of q < 0.001. Dimensionality reduction was performed using the DDRTree reduction method in the reduceDimension step, with no normalization methods applied.

### Nonlinear Model Fitting for Gene Expression Zonation Analysis

To analyze spatial transcriptomics data for understanding gene expression zonation within the liver, nonlinear least squares fitting using the nlsLM method was applied. This analysis helps to elucidate how gene expression varies from the periportal to the pericentral regions. Three distinct modeling approaches were employed to capture the variety of expression patterns:

Polynomial Model: This model was utilized to identify genes exhibiting a gradual change in expression across the liver zones. The mathematical representation involves a second‐degree polynomial equation where the gene expression was modeled as a function of pseudotime, incorporating linear and quadratic terms. Initial parameter values were set to unity, and the model iteratively refined these parameters to achieve the best fit.

Gaussian Peak Model: To detect genes with expression peaks at specific locations, a Gaussian distribution model was fitted to the expression data. This approach was suited for identifying genes with localized maxima in expression along the liver's spatial gradient. The starting parameters for the model included setting the peak height to one, the mean to the average pseudotime across samples, and the standard deviation to one, with adjustments made through iterative fitting.

Inverted Gaussian Peak Model: For genes expected to show a dip in expression within a specific region, surrounded by higher expression levels, an inverted Gaussian model was applied. This model was characterized by subtracting a Gaussian function from a constant, effectively modeling a trough in the gene expression profile. The initial parameters involved setting the constant to the maximum expression observed, with other parameters tuned to capture the expression minimum accurately.

Following model fitting, the variability in expression captured by each model using a normalized range metric, calculated as the difference between the maximum and minimum model values, divided by the maximum value was quantified. This measure helps to distinguish genes with significant zonal expression variation. Genes with a normalized range less than 0.1 were classified as “non‐zonated,” indicating relatively uniform expression across the liver zones. In contrast, genes with greater variability were categorized according to their fitted model, highlighting specific zonation patterns such as periportal, pericentral, midzonal or biporlar.

### Single‐Sample Gene Set Enrichment Analysis with Spatial Transcriptomics Data

Single‐sample gene set enrichment analysis (ssGSEA) using the GSVA package in R to determine the enrichment levels of predefined gene sets was performed. For this analysis, gene sets from the Molecular Signatures Database (MSigDB) was curated and tailored them to the specific biological context of this study. The ssGSEA algorithm ranked the genes within each sample based on their expression levels and calculated an enrichment score that reflected the extent to which the genes in the gene set were coordinately up‐ or down‐regulated. These enrichment scores were computed for each gene set within each spatial spot, enabling us to map pathway activities in a spatial context. The following gene sets were employed: “HALLMARK_KRAS_SIGNALING_UP” for RAS, “HALLMARK_MTORC1_SIGNALING” for mTORC1, “BIOCARTA_PGC1A_PATHWAY” for PGC1α, “MEBARKI_HCC_PROGENITOR_WNT_UP_CTNNB1_DEPENDENT” for WNT, and “KEGG_PPAR_SIGNALING_PATHWAY” for PPARα.

### Cell Culture

AML12 cells were maintained in DMEM supplementedwith10%FBS (Gibco) and1% penicil.lin‐streptomycin (Solarbio). To model liver fasting response, cells were washed twice with PBS and cultured in glucose‐free DMEM (Gibco, #A1443001) with FBS 2% and Palmitic acid (300 µm) for 4 h.^[^ ^6^, ^41^, ^42^ ^]^ Plasmid transfection was carried out using Lipofectamine 3000 transfection reagent (L3000015, Invitrogen). Briefly, cells were seeded in 60 mm dishes to be at 70% confluence on the day of transfection and the medium was changed 24 h post‐transfection. For siRNA transfection, 60 pmol siRNA diluted in siRNA transfection medium was added to 6 µL siRNA transfection reagent (E607402, Sangon Biotech) diluted in an equal volume of siRNA transfection medium. The mixture was allowed to incubate at room temperature for 15 min, and further diluted in siRNA transfection medium before adding to Aml12 cells in a 6‐well plate.

### Immunohistochemistry and Immunofluorescence

Mouse liver tissue samples were fixed overnight in 4% formalin, embedded in paraffin, and sliced into 5 µm thick sections. The slices were microwaved in in 10 mM citrate buffer or Tris‐EDTA buffer (pH 9.0) for 10 min. Subsequently, the sections were blocked in room temperature for 1 h with 2% bovine serum, and incubated overnight with the primary antibody at 4 °C. The following primary antibodies were used: polyclonal Rabbit anti‐Thrsp antibody (1:200, #13054‐1‐AP, Proteintech), polyclonal Rabbit anti‐Gstm3 antibody (1:200, #15214‐1‐AP, Proteintech), monoclonal rat anti‐Cyp51 antibody (1:200, # ab210792, Abcam), polyclonal Rabbit anti‐Gstm3 antibody (1:200, #15214‐1‐AP, Proteintech), polyclonal Rabbit anti‐Bip antibody (1:200, #3177, CST), monoclonal Rabbit anti‐Cd36 antibody (1:200, #ET1701‐24, HUABIO), polyclonal Rabbit anti‐Plin5 antibody (1:200, #26951‐1‐AP, Proteintech), polyclonal Rabbit anti‐Ces1 antibody (1:200, #PA5‐19740, ThermoFisher), polyclonal Rabbit anti‐Ces2 antibody (1:200, #PA5‐102415, ThermoFisher). For immunohistochemical staining, sections were incubated with a secondary antibody (ZLI‐9018, ZSBg‐BIO) coupled with horseradish peroxidase (HRP) at room temperature for 3 h. Color development was achieved using peroxide‐labeled polymers and substrates. For immunofluorescence staining, Car was detected using a rabbit anti‐Car primary antibody (1:50, #A1970, Abclonal) followed by incubation with Multi‐rAb CoraLite® Plus 488‐Goat Anti‐Rabbit recombinant secondary antibody (1:500, #RGAR002, proteintech). Pparα was detected using a mouse anti‐Pparα primary antibody (1:10, #sc‐398394, Santa Cruz Biotechnology) followed by incubation with a Multi‐rAb CoraLite® Plus 594‐Goat Anti‐Mouse recombinant secondary antibody (1:500, #RGAM004, Proteintech). After washing, the sections were counterstained with DAPI (1 µg/mL) for 5 min, mounted with an anti‐fade mounting medium, and visualized using a fluorescence microscope.

### ChIP and Quantitative PCR

The Chromatin Immunoprecipitation (ChIP) Assay Kit (Beyotime, P2083S) was used for ChIP‐qPCR assays, according to the manufacturer's instructions. HEK‐293T were cross‐linked in 1% formaldehyde then nuclei were purified and lysed. Anti‐PPARα (Santa, sc‐398394, dilution: 1:20) and protein A/G agarose beads (Vazyme, PB101) were used in the immunoprecipitation. The purified ChIP DNA samples and their input DNA were diluted and used for ChIP‐qPCR. All ChIP experiments were quantified by quantitative PCR (qPCR) with appropriate primers (Data S3, Supporting Information).

### Dual Luciferase Reporter Assay

A Dual‐Luciferase Reporter Assay System (Promega) was used for the luciferase reporter assay. The promoter regions of Nr1i3, Nr1i3 mutation were synthesized by Qingke Biotechnology Co. and subsequently cloned into pGL‐3 Basic vector (Promega, Madison, WI, USA). In the Nr1i3 dual luciferase reporter assay, HEK‐293T cells were co‐transfected with pGL‐3‐Basic‐Luc, pGL‐3‐Nr1i3‐Luc, pGL‐3‐Nr1i3‐Mut‐Luc, and a Renilla overexpression construct using Lipofectamine 2000 transfection reagent (Invitrogen). WY‐14643 (10 uM, Selleck, S8029) was added 24 h after DNA transfection, and cells were harvested 48 h post‐transfection. For the Ces2a dual luciferase reporter assay, the promoter region of *Ces2a* was amplified by PCR and cloned into pGL‐3 Basic vector (Promega, Madison, WI, USA). The primer sequences employed for cloning were as follows: Full‐length promoter (P1) F: TGGTGCCAAGTATCTCTGCGTG, R: GGCCTGGTCCAAGCAGGAAT; Truncated promoter (P2) F: CCTGTCTTCAGCACAGAGTAGCT, R: GGCCTGGTCCAAGCAGGAAT. HEK‐293T cells were co‐transfected with pGL‐3‐*Ces2a*‐Luc and *pcDNA3*.1‐*Nr1i3*‐flag *or pcDNA3*.1‐flag overexpression construct using Lipofectamine 2000 transfection reagent (Invitrogen). TCPOBOP (250 nM) was added 24 h after DNA transfection, and cells were harvested 48 h after DNA transfection. The dual luciferase reporter assay was performed using Vazyme Kit (DD1205‐01), with Renilla luciferase activity serving as the normalization factor for transfection efficiency.

### In Vitro siRNA Transfection

The AML12 cell line was cultured in DMEM (F‐12) medium supplemented with 10% FBS, 1% penicillin, 1% ITS, and 40 ng mL^−1^ dexamethasone. AML12 cells were transfected with short interfering RNAs (siRNAs) at a concentration of 100 nM using Lipofectamine™ 2000 Transfection Reagent (11 668 019, Thermo Fisher) for 48 h. The cells were then harvested to assess the targeting efficiencies of the siRNAs by qRT‐PCR and Western blot. The siRNAs corresponded to three target sites of the mouse *Ces2a* coding region: si*Ces2a* #1: 5′‐GCCTGATATAATGAATTTAGA‐3′, si*Ces2a* #2: 5′‐TTCCATGAATGATGTATCTAA‐3′ and si*Ces2a* #3: 5′‐GCAGAAGAATATTGCTTACTT‐3′. For in vivo experiments, si*Ces2a* #1 was selected due to its superior knockdown efficiency. The *Ppara* siRNA sequences are as follows: si*Ppara* #1: 5′‐GAAGAAUUCUUACAAGAAATT‐3′, si*Ppara* #2: 5′‐GCAAGAUUCAGAAGAAGAATT‐3′, si*Ppara* #3: 5′‐GACCAAGUCACCUUGCUA‐3′. si*Ppara* #2 was chosen for subsequent experiments due to its superior knockdown efficiency. A non‐targeting siRNA (siNC: 5′‐TTCTCCGAACGTGTCACGTTT‐3′) was used as control.

### In Vivo siRNA Delivery

For in vivo siRNA delivery, the in vivo‐jetPEI™ (Polyplus Inc.) was utilized following the manufacturer's instructions. Each mouse received two injections of siRNA at a concentration of 3 nM. The siRNA sequences employed in the in vivo experiment were as follows: siNC: 5′‐TTCTCCGAACGTGTCACGTTT‐3′ and si*Ces2a* #1: 5′‐GCCTGATATAATGAATTTAGA‐3′.

### Western Blot Analysis

Total protein was isolated from tissue samples using RIPA lysis buffer (50 mM Tris‐HCl, pH 7.4, 150 mM NaCl, 1% NP‐40, 0.5% sodium deoxycholate, 0.1% SDS, 1 mM EDTA) supplemented with protease inhibitor cocktail tablets (HY‐K0011; MedChemExpress) and phosphatase inhibitor tablets (G2007, Servicebio). The total protein samples were loaded and separated on SDS–PAGE gels and transferred onto PVDF membranes (IPVH00010; Merck Millipore). The membranes were blocked with 5% skim milk and incubated with the specified primary antibodies overnight at 4 °C, followed by incubation with the corresponding secondary antibodies for 1 h at room temperature. The membranes were visualized by enhanced chemiluminescence (ECL) reagents (E411‐03; Vazyme) and captured by a Chemiluminescence Imaging System (Tanon 5500). Gapdh was used as a loading control. The following primary antibodies were used: polyclonal Rabbit anti‐Gapdh antibody (1:1000, #AB0037, Abways), polyclonal Rabbit anti‐Ces2 antibody (1:200, #PA5‐102415, ThermoFisher).

### Flow Cytometry and Cell Sorting

Liver cells were isolated via perfusion to facilitate their dissociation. To prepare for FACS, dissociated liver cells were labeled using the Zombie Aqua™ Fixable Viability Kit (BioLegend) to discern live cells. The cells were suspended in chilled PBS at a density of 10^6 cells per 100 µl. Zombie Aqua™ was applied at a 1:1000 dilution. The cell suspension was then rotated in darkness at room temperature for 30 min. Following centrifugation (1000 rpm, 5 min, 4 °C), the pellet was resuspended in FACS buffer (2 mM EDTA, pH 8.0, plus 0.5% BSA in 1x PBS) to the original concentration. Cells were stained with Brilliant Violet 421™ anti‐mouse/human CD324 (E‐Cadherin) Antibody (BioLegend, 147 319), APC‐anti‐CD73 (BioLegend, 127 210), PE‐Cy7‐anti‐CD31 (BioLegend, 102 418), and Alexa Fluor™ 700‐anti‐CD45.2 (eBioscience, 56‐0454‐82) at a 1:200 dilution. FcX blocking solution (BioLegend) was included at a 1:100 dilution. The cell mixture was incubated on a rotator in the dark at 4 °C for another 30 min. Finally, the cells were resuspended in FACS buffer and sorted using the MoFlo Astrios EQS (BECKMAN COULTER).

### Quantitative Real‐Time PCR

Total RNA was extracted with Total RNA Extraction Reagent (R401‐01, Vazyme) and then reverse transcription was performed using a reverse transcriptase kit (R233‐01, Vazyme). Using cDNA as template, CFX Connect Real‐Time PCR Detection System (BIO‐RAD) was used for real‐time quantitative PCR. *Gapdh* was used as the normalized control. The primers used in this study are listed in Table S3 (Supporting Information).

### Liver TG/FAA Measurement

Mice liver samples (50‐100 mg) was homogenized by Tissue Lyser. Triglyceride were measured using a commercially available kit (BC0625, Solarbio) and Free Fatty acid assay kit (BC0595, Solarbio). According to the manufacturer's instructions, the extracted homogenates were determined using a 96‐well enzymoscope (BIO‐RAD) at 420/550 nm wavelength.

### Plasma Glucose Assay

Blood glucose in mice were measured directly via blood from the tail tips using a glucometer.

### Serum β‐HB Assay

Serum β‐HB were measured using a commercially available kit (BC5085, Solarbio). According to the manufacturer's instructions, the extracted homogenates were determined using a 96‐well enzymoscope (BIO‐RAD) at 450 nm wavelength.

### Statistical Analysis

The statistical significance of differences between groups was examined using unpaired two‐tailed Student's t test. Statistically significant differences were considered as *P < 0.05, **P < 0.01, ***P < 0.001 and ****P < 0.0001. All data were expressed as the mean ± SEM values and were analyzed using GraphPad Prism 7.0 (GraphPad Software Inc.).

### Data and Code Availability

The raw and processed data used for this study have been deposited on GEO with accession numbers GSE222796. Any additional information required to reanalyze the data reported in this paper is available from the lead contact upon request.

## Conflict of Interest

The authors declare no conflict of interest.

## Author Contributions

S.W., B.X., J.L, P.H., Y.F., and P.H. contributed equally to this work. D.Y., S.L., Z.L. and Y.Z. conceived this study, generated hypotheses, designed experiments, and wrote, reviewed and edited the paper. S.W. performed the ST experiment. S.W., B.X., P.H., X.L., Y.F., and J.L. performed in vivo experiments and biochemical experiments. P.Z., P.M., C.Z., Z.L., S.L., and D.Y. contributed to the acquisition of financial support for the study. All authors discussed the results and contributed to the final manuscript.

## Supporting information

Supporting Information

## Data Availability

The data that support the findings of this study are openly available in GEO Database at https://www.ncbi.nlm.nih.gov/geo/, reference number 222796.
