# Supplementary material for: Spatial Transcriptomic Study Reveals Heterogeneous Metabolic Adaptation and a Role of Pericentral PPARα/CAR/Ces2a Axis During Fasting in Mouse Liver
Source: Adv Sci (Weinh). 2024 Sep 5;11(41):2405240. doi: 10.1002/advs.202405240 (PMC11538668; doi:10.1002/advs.202405240)
Supplement: Supplementary file 1 — Supporting Information [file ADVS-11-2405240-s001.docx]

Supplemental information

# Spatial transcriptomic study reveals heterogeneous metabolic adaptation and a role of pericentral PPARα/CAR/Ces2a axis during fasting in mouse liver

Shiguan Wang,^1,2,6^ Bowen Xu,^1,3,6^ Penghu Han,^3,6^ Yawei Feng,^1,6^ Jinyuan Liang,^1,6^ Jing Shen,^3^ Xinying Li,^1^ Mengqi Zheng,^3^ Tingguo Zhang,^4^ Cuijuan Zhang,^4^ Ping Mi,^1^ Yi Zhang,^2,*^ Zhiping Liu,^5,*^ Shiyang Li,^3,*^ Detian Yuan^1,7,*^

^1^ Department of Biochemistry and Molecular Biology, School of Basic Medical Sciences, Cheeloo College of Medicine, Shandong University, Jinan 250012, China

^2^ Department of Clinical Laboratory, Qilu Hospital of Shandong University, Jinan 250012, China

^3^ Advanced Medical Research Institute, Shandong University, Jinan 250012, China

^4^ Institute of Pathology and Pathophysiology, School of Basic Medical Sciences, Cheeloo College of Medicine, Shandong University, Jinan, Shandong 250012, China

^5^ Department of Biomedical Engineering, School of Control Science and Engineering, Shandong University, Jinan, Shandong 250061, China

^6^ These authors contributed equally: Shiguan Wang, Bowen Xu, Penghu Han, Yawei Feng, Jinyuan Liang.

^7^ Lead contact

^*^ Correspondence:

yizhang@sdu.edu.cn (Y.Z.)

zpliu@sdu.edu.cn (Z.L.)

lishiyang@sdu.edu.cn (S.L.)

yuandt@sdu.edu.cn (D.Y.)

**Supplementary Figure 1**

**
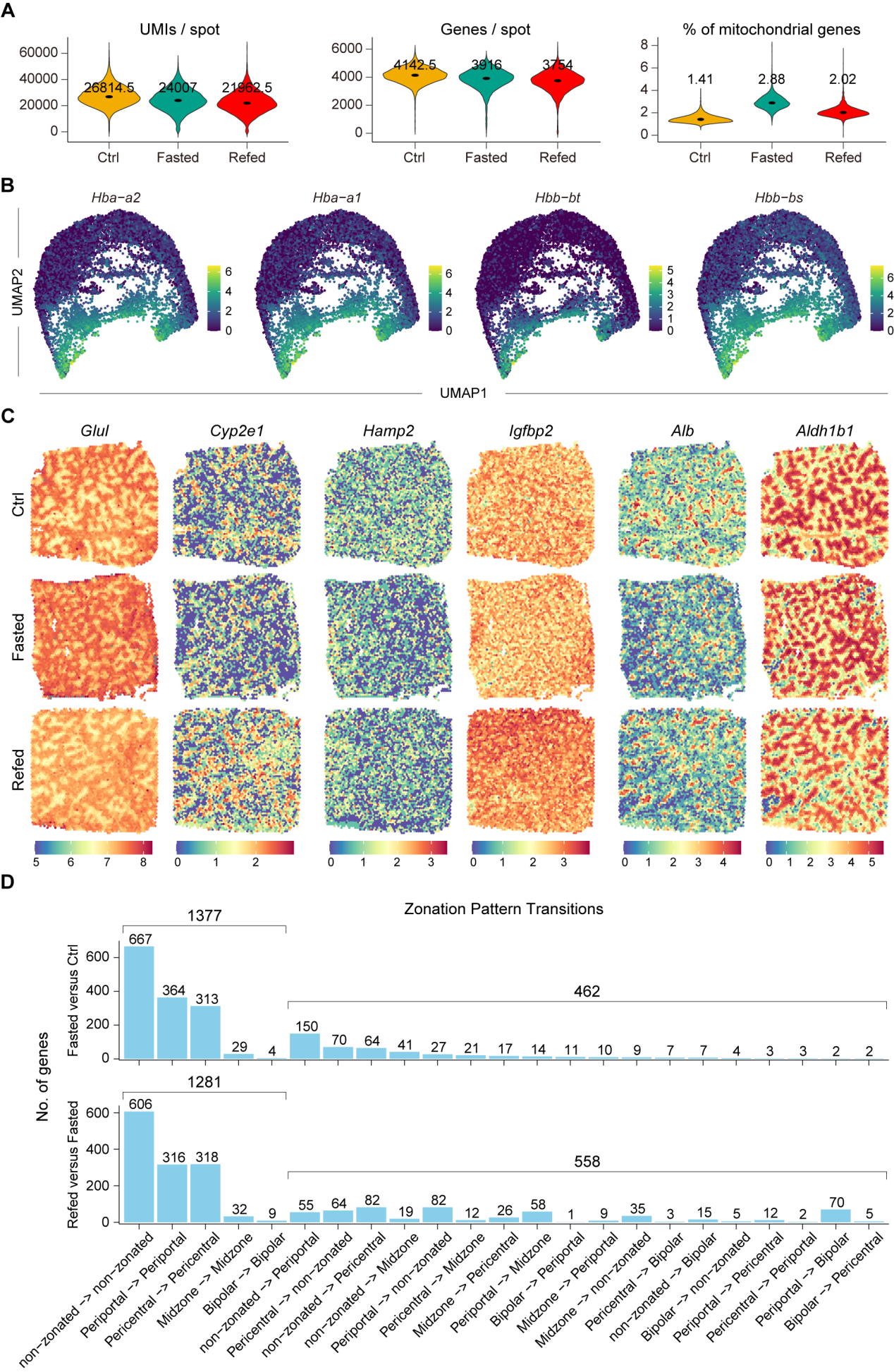
**

**Figure S1.** **Quality control and analysis of the zonation pattern changes in spatial transcriptomics sequencing results.**

1. Evaluation of sequencing metrics for ctrl, fasted, and refed samples by ST, including the average number of unique molecular identifiers (UMIs) per spot (left panel), average number of genes detected per spot (middle panel), and proportions of mitochondrial transcripts (right panel).
2. Visualization of selected genes projected onto UMAP space.
3. Visualized of expression for the indicated genes in tissue spots.

(D) Quantification of the transitions in zonation patterns under three nutritional conditions.

**Supplementary Figure 2**

**
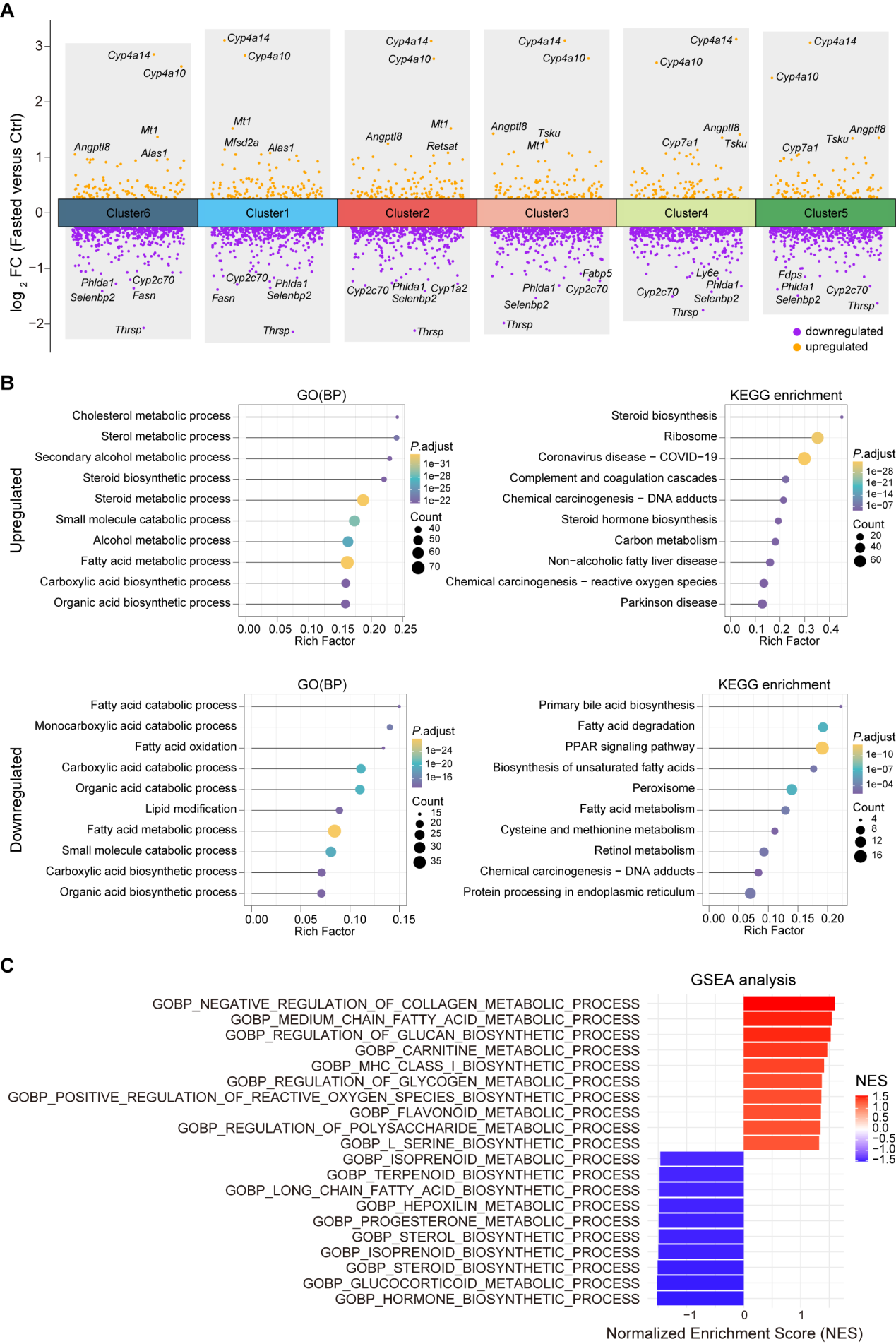
**

**Figure S2. Analysis of functional enrichment of genes downregulated and upregulated during fasting.**

(A) Identification of significantly upregulated and downregulated genes within the six clusters representing hepatocytes along the portal-central axis.

(B) Left: Gene Ontology Biological Process (GOBP) enrichment analysis of downregulated and upregulated genes in livers during fasting. Right: Kyoto Encyclopedia of Genes and Genomes (KEGG) pathway enrichment analysis of downregulated and upregulated genes in livers during fasting.

(C) GSEA comparison of fasting versus Ctrl conditions, highlighting the top 10 upregulated and downregulated genesets derived from GOBP terms from the Molecular Signatures Database (MSigDB).

**Supplementary Figure 3**

**
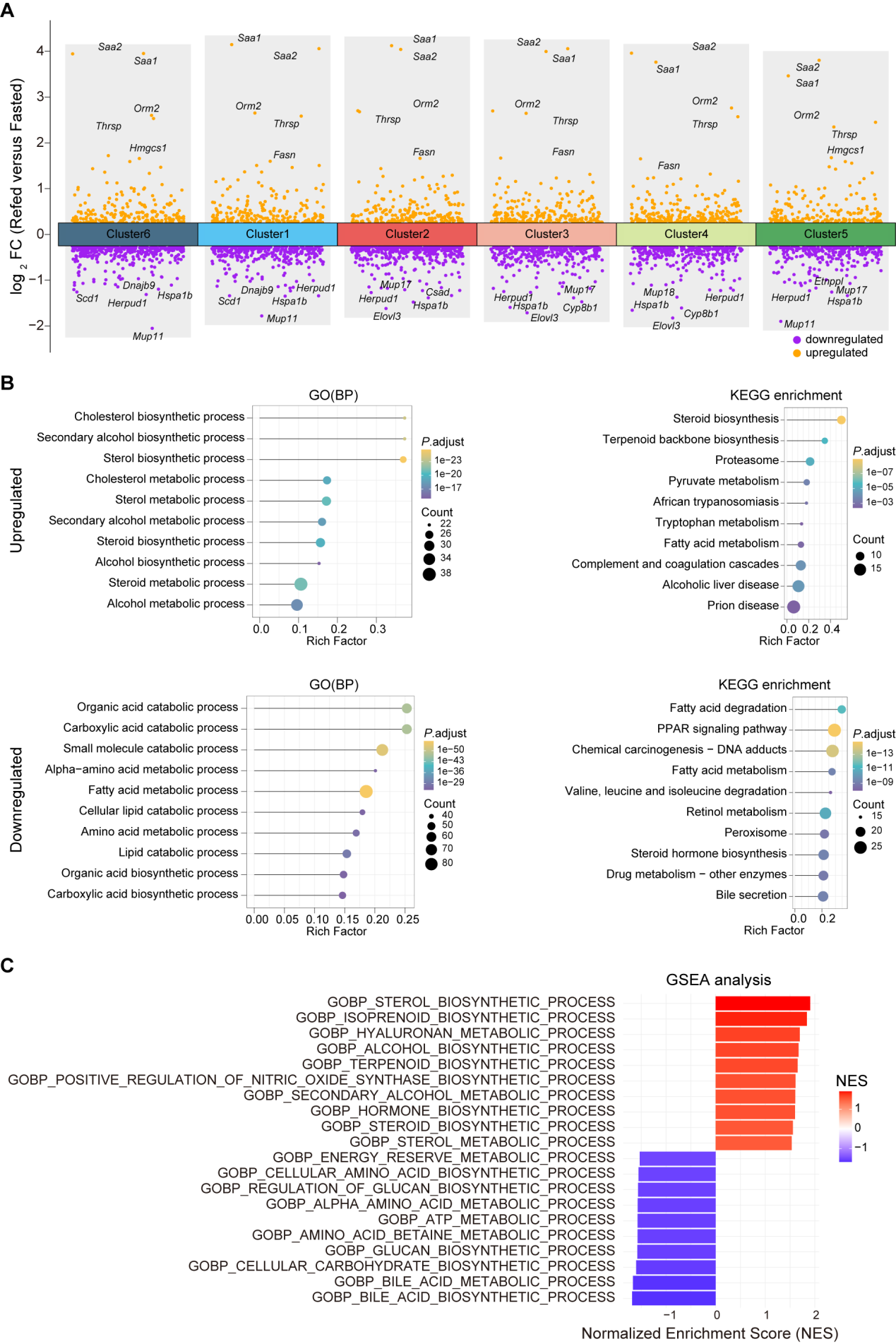
**

**Figure S3. Analysis of functional enrichment of genes downregulated and upregulated during refeeding compared to fasting**

(A) Identification of significantly upregulated and downregulated genes within the six clusters representing hepatocytes along the portal-central axis.

(B) Left: Gene Ontology Biological Process (GOBP) enrichment analysis of downregulated and upregulated genes in livers during refeeding compared to fasting. Right: Kyoto Encyclopedia of Genes and Genomes (KEGG) pathway enrichment analysis of downregulated and upregulated genes in livers during refeeding compared to fasting.

(C) GSEA comparison of refeeding versus fasting conditions, highlighting the top 10 upregulated and downregulated genesets derived from GOBP terms from the Molecular Signatures Database (MSigDB).

**Supplementary Figure 4**

**
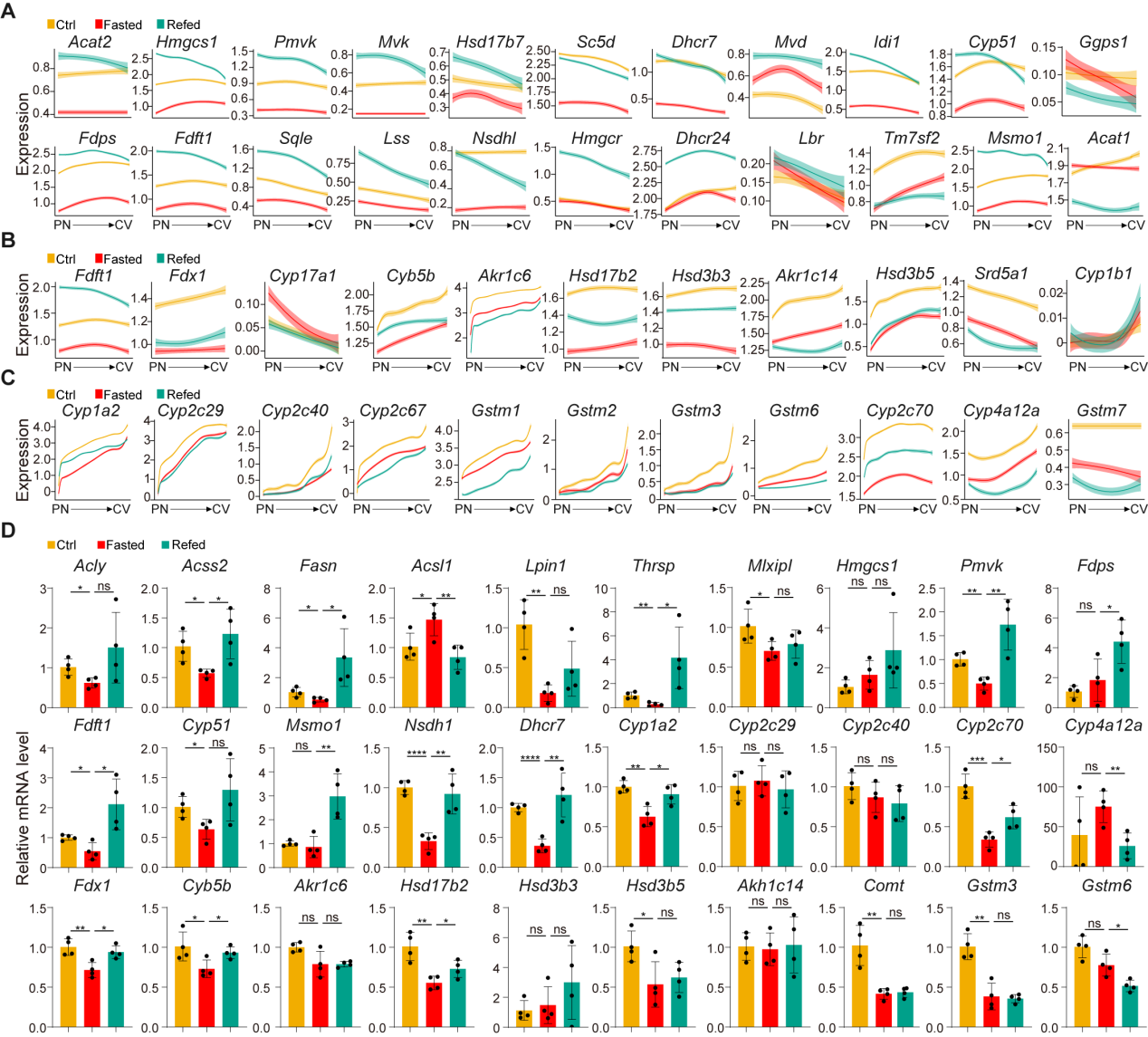
 Figure S4.** **Genes downregulated according to ST during fasting and qRT-PCR validation.**

1. Expression of genes involved in cholesterol biosynthesis along the portal-central axis.
2. Expression of genes associated with steroid hormone biosynthesis evaluated using ST along the portal-central axis.
3. Expression of genes involved in hepatic oxidative (phase I) and conjugative (phase II) pathways for xenobiotic biotransformation along the portal-central axis.
4. qRT-PCR analysis was performed to assess mRNA expression levels of the indicated genes involved in de novo lipogenesis, cholesterol biosynthesis, steroidogenesis and xenobiotic biotransformation in liver samples obtained from ctrl, fasted, and refed mice. n = 4 mice per group.

Data are shown in mean ± SEM; ns, not significant, *p < 0.05, **p < 0.01, ***p < 0.001, ****p < 0.0001 by Student’s t test.

**Supplementary Figure 5**

**
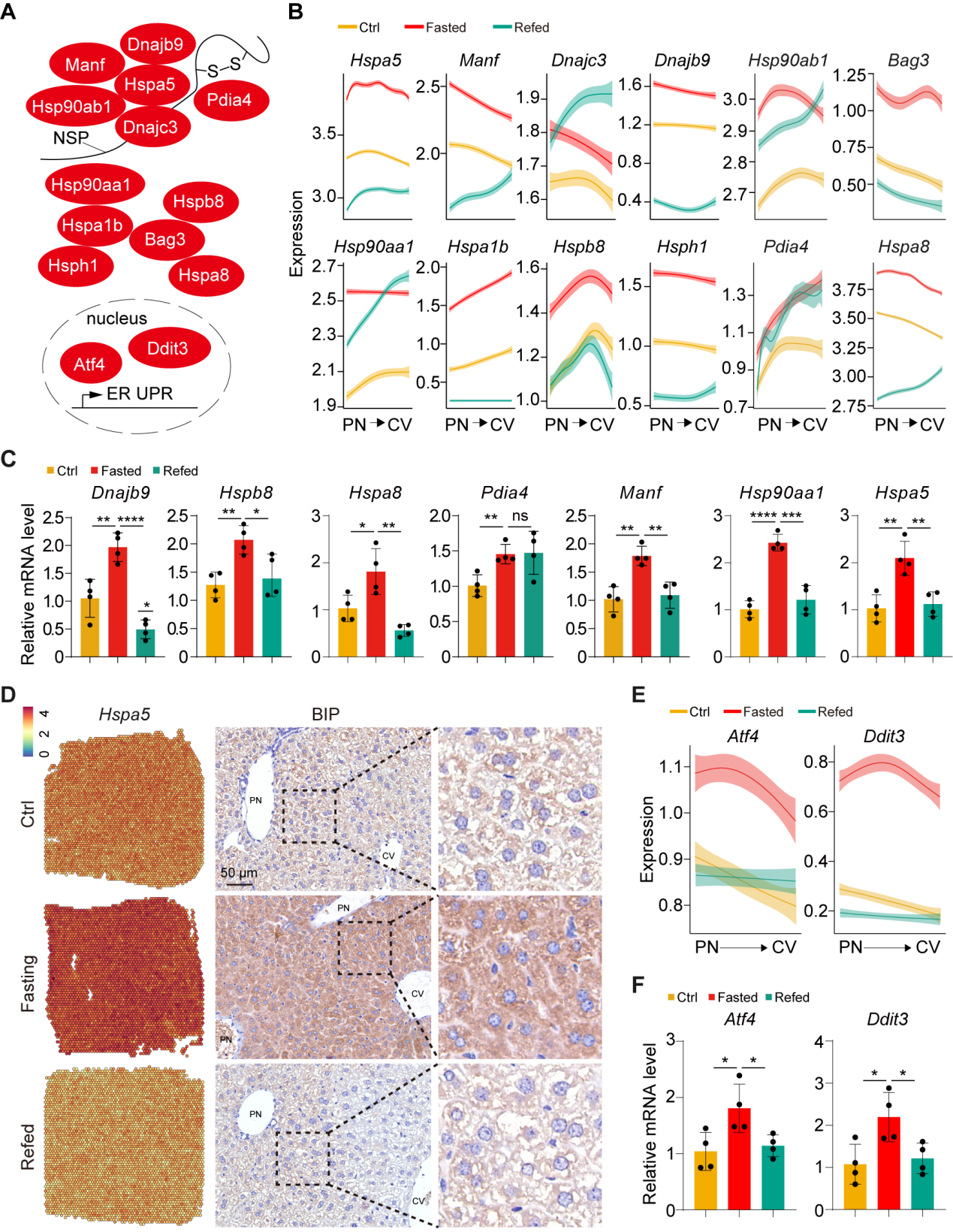
**

**Figure S5.** Molecular chaperons are upregulated during fasting.

1. Upregulated ISR-related genes during fasting revealed by ST.
2. Quantified profiles of the indicated genes along the PN-CV axis from ST.
3. qRT-PCR analysis of mRNA expression levels for selected ISR-related genes in mouse livers from the ctrl, fasted and refed groups. n = 4 mice per group.
4. Visualization of *Hspa5* in tissue spots and IHC of Hspa5 in liver sections.
5. Line graph depicting changes in expression of *Atf4* and *Ddit3* along the portal-central axis in the three experimental groups.
6. mRNA levels of the *Atf4* and *Ddit3* in liver samples obtained from the ctrl, fasted and refed mice. n = 4 mice per group.

Data are shown in mean ± SEM; ns, not significant, **p < 0.01, ****p < 0.0001 by Student’s t test.

**Supplementary Figure 6**


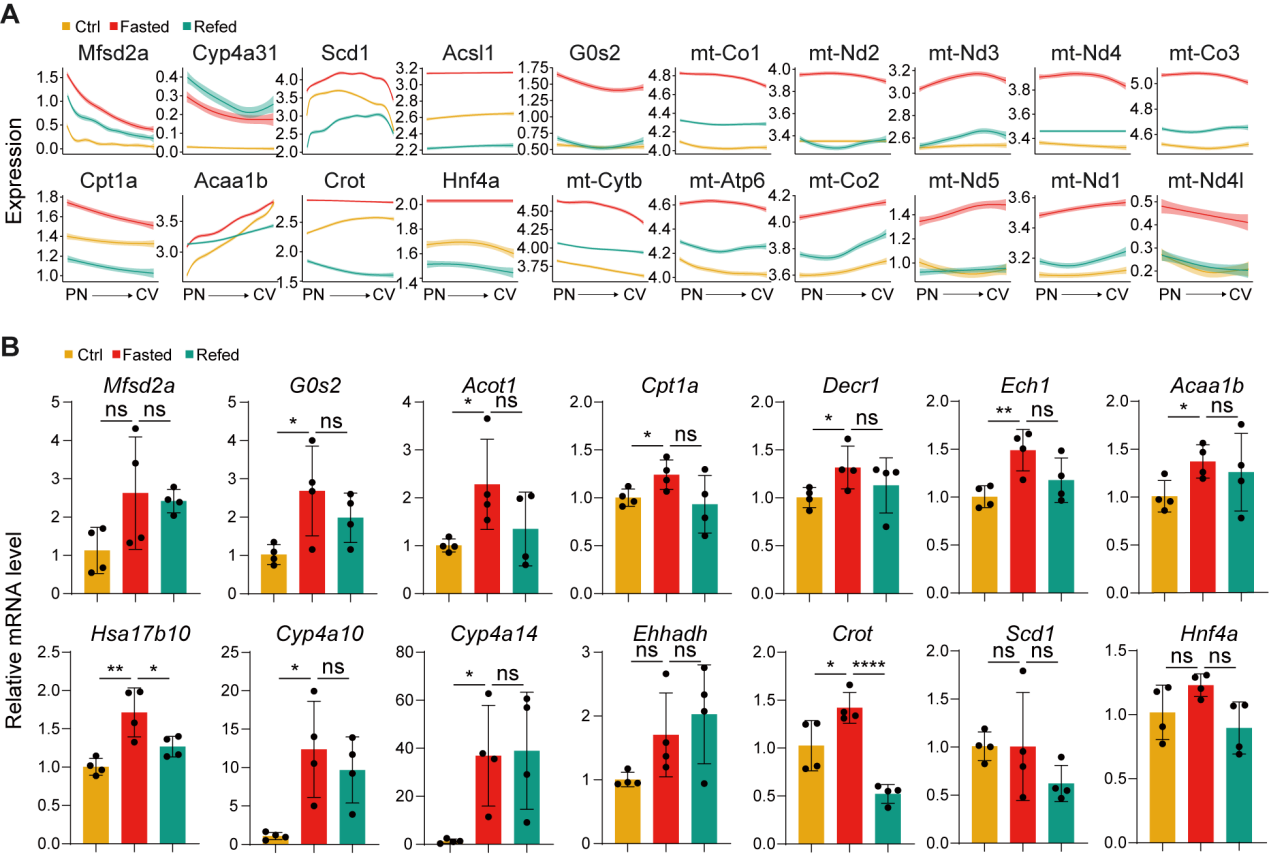


**Figure S6.** **Expression of FAO-related genes revealed by ST and qRT-PCR validation of the specific genes during fasting.**

1. Line graph depicting changes in expression of FAO-related genes along the portal-central axis in the three experimental groups.
2. qRT-PCR analysis of mRNA expression levels for the indicated genes in mouse livers from the ctrl, fasted and refed groups. n = 4 mice per group.

Data are shown in mean ± SEM; ns, not significant, *p < 0.05, **p < 0.01, ****p < 0.0001 by Student’s t test.

**Supplementary Figure 7**


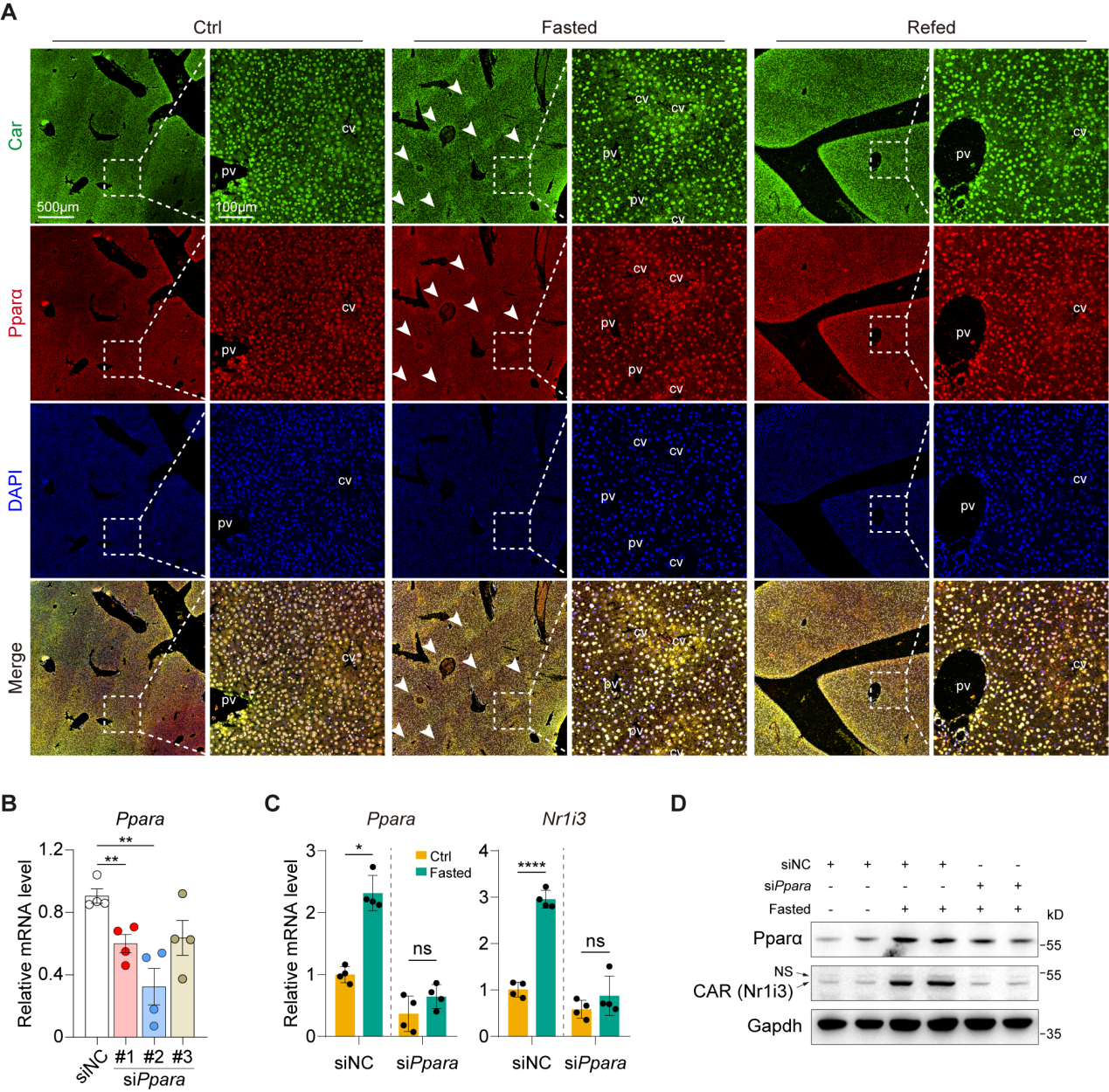


**Figure S7. Pparα-Mediated regulation of CAR expression**

1. Immunofluorescent co-staining of CAR (green) and PPARα (red) in liver sections under different states as indicated. The periportal vein (pv) and central vein (cv) are labeled. White arrowheads highlight the upregulation of both PPARα and CAR in the pericentral zone (cv) following fasting.
2. Quantitative RT-qPCR analysis of Ppara mRNA levels in AML12 cells transfected with three different siRNA sequences targeting Ppara (siPpara #1, #2, #3). Data are presented as mean ± SEM (n = 4), with statistical significance indicated by **p < 0.01.
3. Quantitative RT-qPCR analysis of Ppara and Nr1i3 (CAR) mRNA levels in AML12 cells transfected with either non-targeting siRNA (siNC) or siPpara #2, under control (Ctrl) or fasting conditions. Data are presented as mean ± SEM (n = 4), with statistical significance indicated by *p < 0.05, ****p < 0.0001, ns: not significant.
4. Western blot analysis of Ppara and CAR (Nr1i3) protein levels in AML12 cells transfected with either siNC or siPpara #2, under control or fasting conditions. Gapdh was used as a loading control.

**Supplementary Figure 8**

**
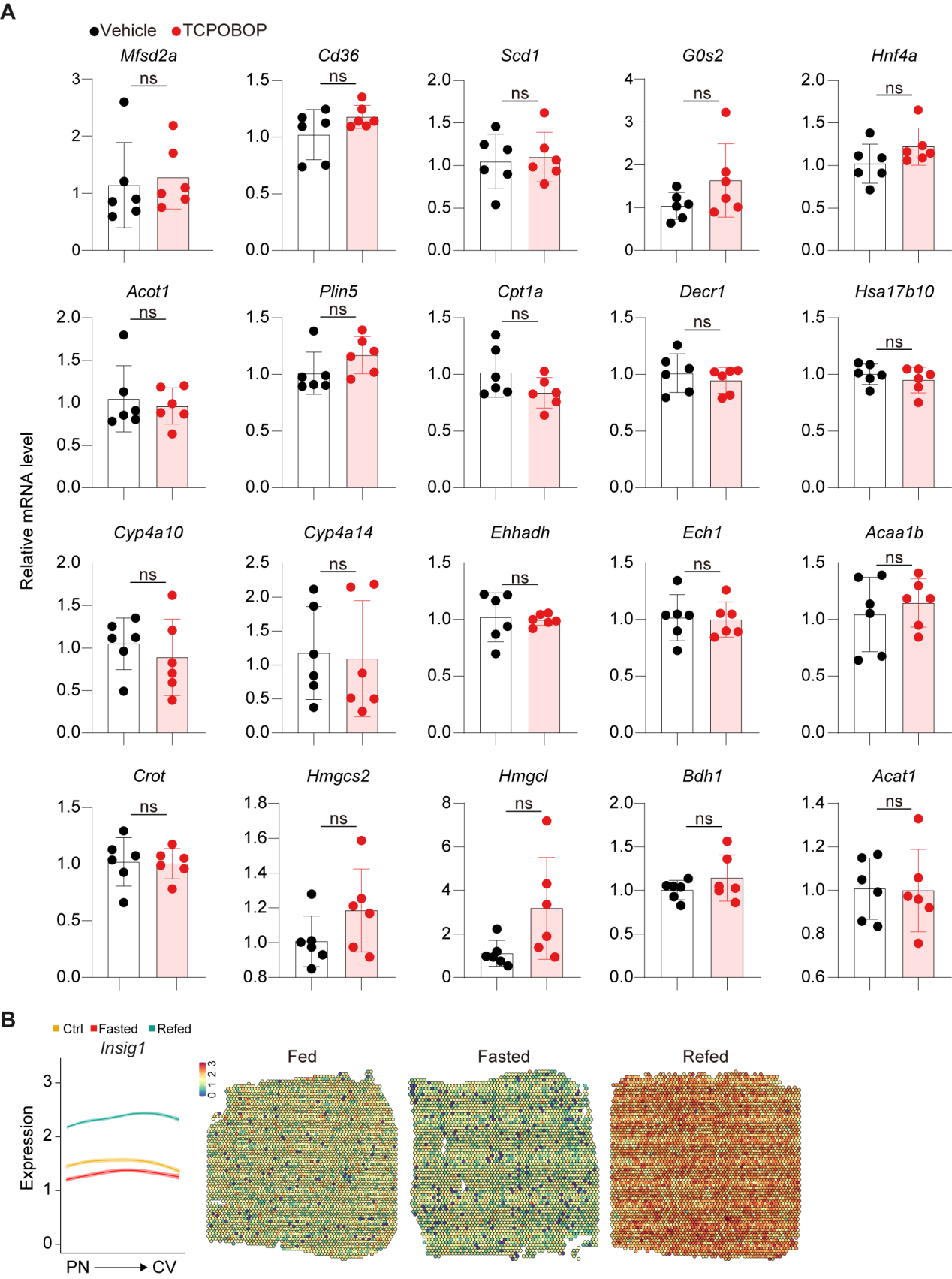
**

**Figure S8. Effects of CAR activation on the expression of key components of FAO and ketogenesis pathways and** ***Insig1* expression analyzed by ST**

1. qRT-PCR analysis of mRNA levels for the indicated genes in mouse livers following TCPOBOP treatment. n = 6 mice per group.
2. Left: Quantified profile of *Insig1* along the portal-central axis using ST. Right: Spatial gene expression distribution of *Insig1* across the liver sections.

Data are shown in mean ± SEM; ns, not significant by Student’s t test.

**Supplementary Figure 9**


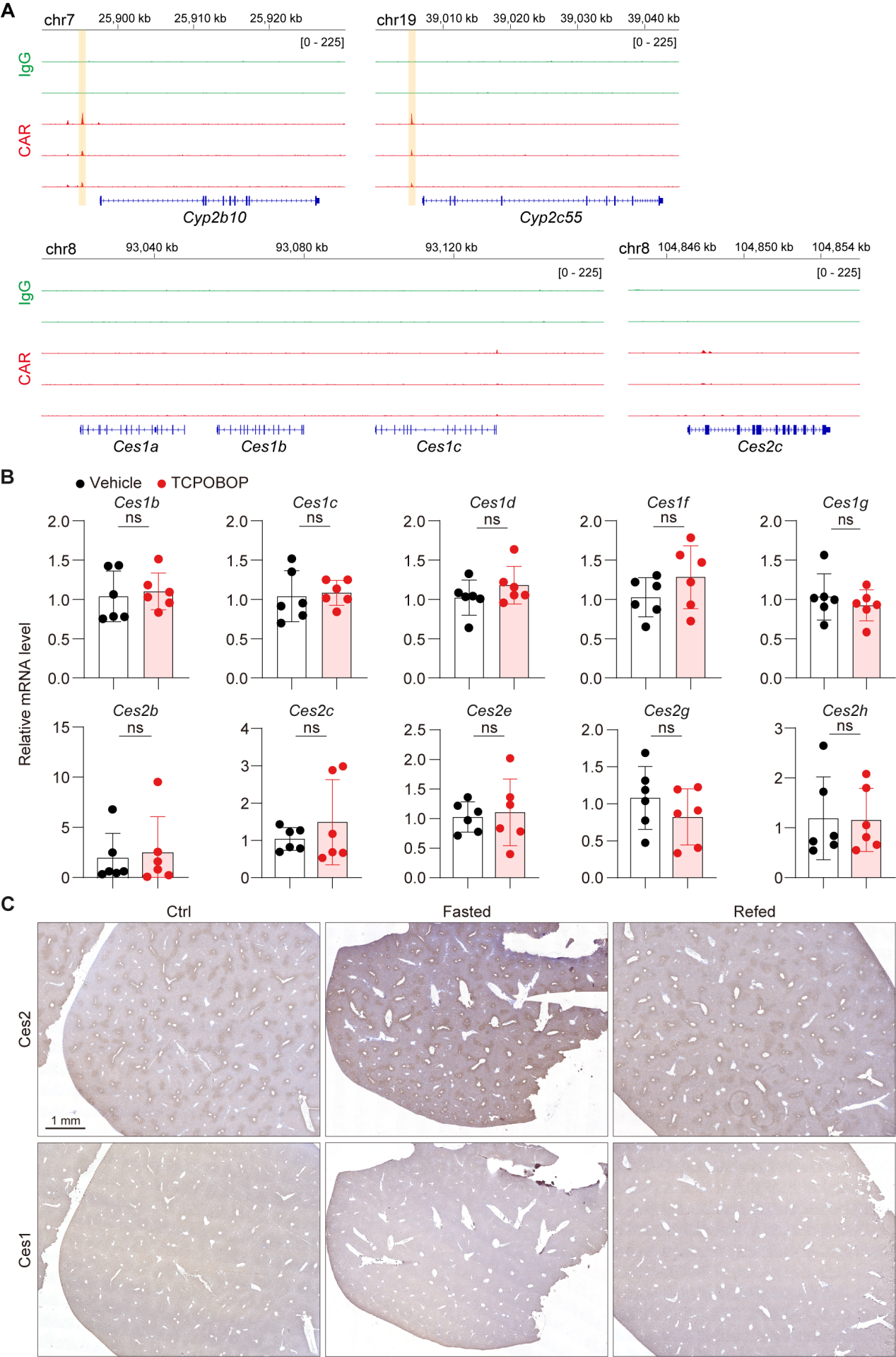


**Figure S9.** **Ces2a as a downstream target of CAR in mouse liver in response to fasting.**

1. Genome browser tracks illustrating the binding of CAR and IgG at the promoters of *Cyp2b10*, *Cyp2c55*, *Ces1a*, *Ces1b*, *Ces1c*, and *Ces2c* genes, as indicated in the panel.
2. qRT-PCR analysis of liver samples from vehicle and TCPOBOP-treated mice for the indicated Ces family genes, as shown in the panel. n = 6 mice per group.
3. Representative IHC images of Ces2 and Ces1 in liver samples from ctrl, fasted and refed mice. Scale bar: 1 mm.

Data are shown in mean ± SEM; ns, not significant by Student’s t test.

**Supplementary Figure 10**


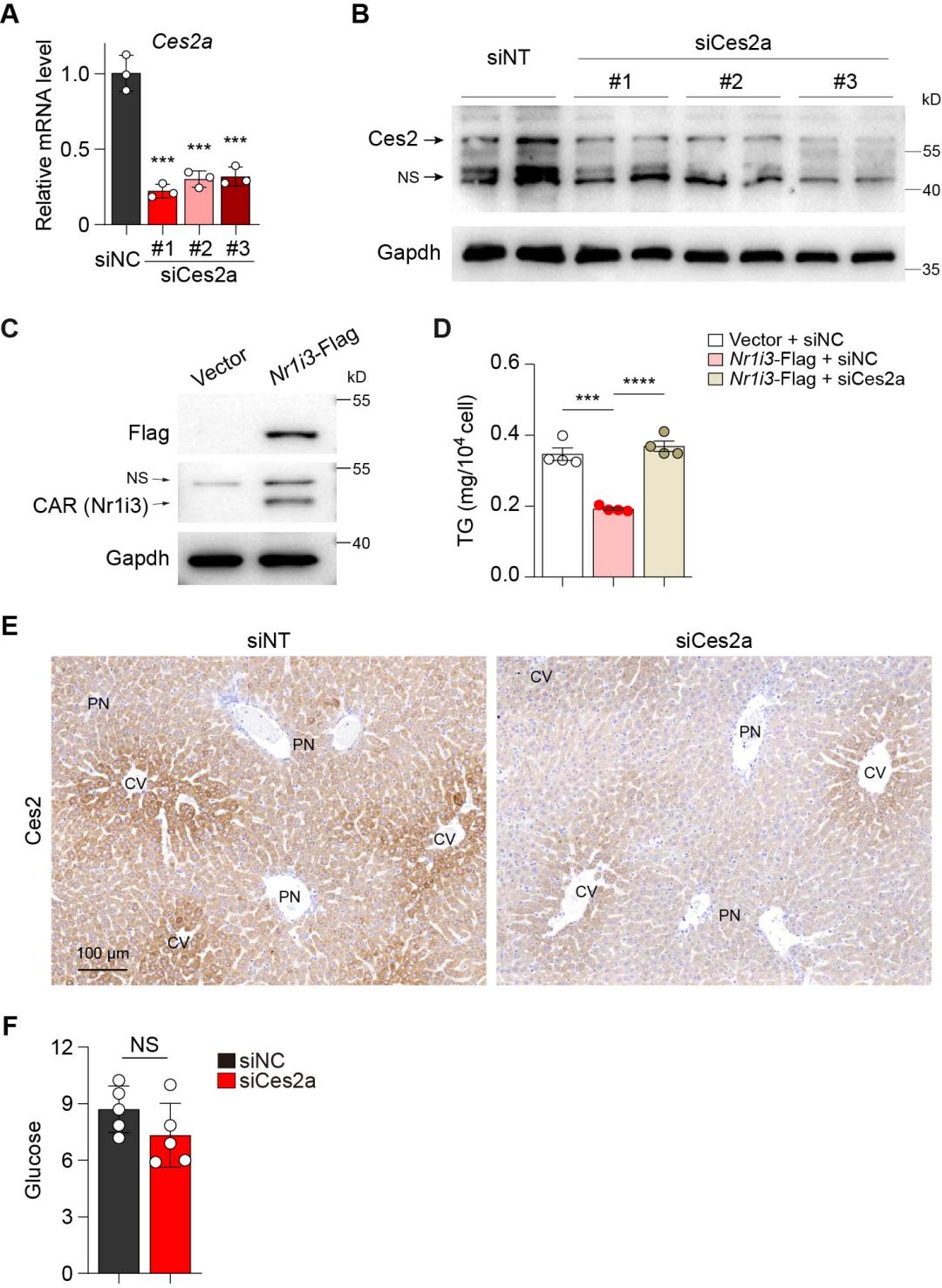


**Figure S10.** **Impact of liver *Ces2a* knockdown on fasting response.**

(A, B) Evaluation of the silencing efficiency of siRNAs targeting *Ces2a* in the murine liver cell line AML12 using qRT-PCR (A) (n=3) and immunoblotting (B).

(C) Western blot showing the overexpression of Flag-tagged CAR in AML12 cells.

(D) TG levels measured in AML12 cells transfected with either *Nr1i3*-Flag and si*Ces2a* or non-targeting control siRNA (siNC). Data are presented as mean ± SEM (n = 4).

(E) Knockdown efficiency of Ces2 in mouse livers detected by IHC.

(F) Effects of Ces2a knockdown on serum levels of glucose in mice following overnight fasting. n = 5 mice per group.

Data are shown in mean ± SEM; ns, not significant, ***p < 0.001, ****p < 0.0001 by Student’s t test.

Table S1

|  | logFC | AveExpr | t | P.Value | adj.P.Val | B |
| --- | --- | --- | --- | --- | --- | --- |
| Ppia | -0.250298672 | 2.775596738 | -17.4123608 | 1.51515E-63 | 8.26201E-62 | 133.2775156 |
| Otc | -0.251820818 | 2.407261994 | -14.49503116 | 1.86403E-45 | 7.62332E-44 | 91.79274114 |
| Ndufab1 | -0.25186306 | 1.556399095 | -10.02624618 | 3.82653E-23 | 8.98216E-22 | 40.76291108 |
| Chid1 | -0.251922761 | 0.516830974 | -9.616137252 | 1.87233E-21 | 4.10928E-20 | 36.91208686 |
| Gstm2 | -0.252234071 | 0.342552941 | -10.90008024 | 5.95237E-27 | 1.58477E-25 | 49.45237946 |
| Rpl3 | -0.252281144 | 2.488909105 | -14.88988485 | 9.43537E-48 | 4.10193E-46 | 97.05384191 |
| Wdr83os | -0.252406985 | 0.748560597 | -8.826160383 | 2.22826E-18 | 4.33841E-17 | 29.91159435 |
| Tcp11l2 | -0.252453308 | 0.20792215 | -13.40990876 | 2.08567E-39 | 7.6966E-38 | 77.93735739 |
| Ghr | -0.252635166 | 1.825241263 | -11.30350717 | 8.36567E-29 | 2.34121E-27 | 53.68289978 |
| Bola3 | -0.252765177 | 1.411256171 | -9.550240612 | 3.45126E-21 | 7.50201E-20 | 36.30704777 |
| Rps15a | -0.252916766 | 2.676661875 | -16.45633114 | 2.45341E-57 | 1.25989E-55 | 119.0317929 |
| Lamp1 | -0.25302324 | 2.292639405 | -13.91931256 | 3.37121E-42 | 1.31114E-40 | 84.33015329 |
| Cdh1 | -0.253851372 | 0.389781687 | -10.44230875 | 6.37806E-25 | 1.58881E-23 | 44.8186906 |
| Romo1 | -0.253985203 | 1.340657805 | -9.326395136 | 2.67882E-20 | 5.61525E-19 | 34.28031679 |
| Rpl24 | -0.254149152 | 2.805027382 | -17.82818143 | 2.50303E-66 | 1.4185E-64 | 139.661737 |
| Srpr | -0.254438839 | 1.017213413 | -8.83948209 | 1.98643E-18 | 3.87708E-17 | 30.02504595 |
| Acsf2 | -0.255042293 | 0.284140513 | -11.97892573 | 4.90981E-32 | 1.51574E-30 | 61.06889589 |
| Aldh4a1 | -0.255065984 | 1.580237064 | -10.26528934 | 3.70721E-24 | 8.96738E-23 | 43.07486844 |
| Entpd8 | -0.255168298 | 0.716006226 | -9.166034733 | 1.13196E-19 | 2.31767E-18 | 32.85560623 |
| Agmat | -0.255928419 | 1.171356026 | -9.089069681 | 2.2426E-19 | 4.56226E-18 | 32.17991713 |
| Slco2b1 | -0.255951974 | 0.877039057 | -8.735032127 | 4.86896E-18 | 9.331E-17 | 29.13978316 |
| Aldh8a1 | -0.256595334 | 1.699400455 | -10.58136762 | 1.57089E-25 | 3.96924E-24 | 46.20740346 |
| F13b | -0.256759063 | 1.153675254 | -9.118354139 | 1.72999E-19 | 3.52846E-18 | 32.43638989 |
| Prdx3 | -0.257726945 | 0.74910953 | -9.022111621 | 4.04792E-19 | 8.13068E-18 | 31.59636651 |
| Nme2 | -0.258104165 | 2.867283364 | -18.5292489 | 3.98262E-71 | 2.47828E-69 | 150.6751033 |
| Ufc1 | -0.258263672 | 1.321072717 | -9.435263732 | 9.94191E-21 | 2.10625E-19 | 35.26051313 |
| Aldh1l1 | -0.258271666 | 3.315979374 | -22.51932204 | 8.4889E-101 | 7.36079E-99 | 218.8199408 |
| Cyp27a1 | -0.258360785 | 1.689396359 | -10.68718071 | 5.34977E-26 | 1.3626E-24 | 47.27516787 |
| Ttc23 | -0.260011444 | 0.554220917 | -9.933519231 | 9.33962E-23 | 2.16036E-21 | 39.87940503 |
| Rnf103 | -0.26083999 | 0.858365127 | -9.184828904 | 9.57156E-20 | 1.96229E-18 | 33.02140261 |
| Atp5g1 | -0.261211067 | 2.254310915 | -13.59808645 | 1.98649E-40 | 7.48732E-39 | 80.27565225 |
| Prdx5 | -0.262296216 | 1.823284441 | -11.66160377 | 1.69603E-30 | 4.95628E-29 | 57.55193742 |
| Acy3 | -0.263714652 | 0.71091692 | -9.383934786 | 1.5885E-20 | 3.3519E-19 | 34.79707042 |
| Lims2 | -0.264005383 | 0.568280649 | -9.776696331 | 4.15348E-22 | 9.32212E-21 | 38.40221572 |
| Rpl36a | -0.264551801 | 2.239168509 | -14.36475217 | 1.03982E-44 | 4.16661E-43 | 90.08224258 |
| Copz1 | -0.264693452 | 1.543644225 | -10.28021366 | 3.19928E-24 | 7.77429E-23 | 43.22084822 |
| Eif5a | -0.26513961 | 2.173037176 | -14.56316008 | 7.5492E-46 | 3.13588E-44 | 92.69227322 |
| Fpgs | -0.265262314 | 0.923757779 | -9.469154645 | 7.28698E-21 | 1.55E-19 | 35.56778311 |
| Cox6b1 | -0.265294217 | 2.305291322 | -14.87129757 | 1.21321E-47 | 5.23132E-46 | 96.80360686 |
| Taf10 | -0.26551933 | 1.117409296 | -9.277481143 | 4.16764E-20 | 8.66737E-19 | 33.84333003 |
| Fkbp8 | -0.265628025 | 1.665712991 | -10.9175469 | 4.9627E-27 | 1.32796E-25 | 49.63270589 |
| Aqp9 | -0.265938754 | 1.295231826 | -9.745418673 | 5.57914E-22 | 1.24462E-20 | 38.1101628 |
| Gm26917 | -0.266164995 | 0.53916359 | -9.820181888 | 2.75186E-22 | 6.20263E-21 | 38.80967859 |
| Ndufa13 | -0.266225677 | 2.47084371 | -15.773299 | 4.58484E-53 | 2.21131E-51 | 109.2351971 |
| Fau | -0.266254601 | 3.371062428 | -24.19928389 | 2.3503E-114 | 2.4217E-112 | 249.9733957 |
| Gstt1 | -0.267014035 | 1.345013988 | -10.1622329 | 1.02024E-23 | 2.42715E-22 | 42.07208001 |
| St3gal3 | -0.268165259 | 0.850154278 | -9.393644308 | 1.454E-20 | 3.07219E-19 | 34.88455825 |
| Rpl34 | -0.268423255 | 2.784832429 | -19.49037981 | 6.44638E-78 | 4.31608E-76 | 166.2657556 |
| Adra1b | -0.268430613 | 0.465147576 | -10.22378888 | 5.57914E-24 | 1.33933E-22 | 42.6699473 |
| Cyp2j6 | -0.268468243 | 0.918973084 | -9.379172755 | 1.65889E-20 | 3.49578E-19 | 34.75419246 |
| Tomm7 | -0.268496214 | 1.276561537 | -9.770938508 | 4.38562E-22 | 9.82925E-21 | 38.34838827 |
| Hykk | -0.269987728 | 0.524829139 | -10.33007034 | 1.95285E-24 | 4.77471E-23 | 43.70990316 |
| Rpl18a | -0.270901001 | 2.855176044 | -18.94786811 | 4.69968E-74 | 3.01921E-72 | 157.3970181 |
| Tuba1c | -0.271240989 | 1.580175392 | -10.87392358 | 7.81167E-27 | 2.06249E-25 | 49.18281947 |
| Ndufa11 | -0.271278655 | 1.391341798 | -9.894295554 | 1.35921E-22 | 3.11226E-21 | 39.50792943 |
| Rps25 | -0.271756849 | 2.28417409 | -15.02824046 | 1.44098E-48 | 6.29901E-47 | 98.92441901 |
| Edf1 | -0.271777936 | 1.865582669 | -12.43905082 | 2.49801E-34 | 8.1393E-33 | 66.31439504 |
| Gstm3 | -0.272045436 | 0.255975727 | -13.23889545 | 1.72577E-38 | 6.25218E-37 | 75.8361802 |
| Cp | -0.273549003 | 2.161812867 | -13.6076231 | 1.76206E-40 | 6.65724E-39 | 80.39488279 |
| Selenow | -0.273761792 | 1.461374498 | -10.16952019 | 9.50038E-24 | 2.26355E-22 | 42.14268763 |
| Rps3 | -0.27410198 | 2.307437502 | -15.20321968 | 1.31195E-49 | 5.86424E-48 | 101.3100955 |
| Rbbp4 | -0.274334407 | 0.740577637 | -9.745314439 | 5.58462E-22 | 1.24462E-20 | 38.10919095 |
| Lamtor4 | -0.274355381 | 1.46089242 | -10.61438608 | 1.12359E-25 | 2.8481E-24 | 46.5395699 |
| Ugt2b38 | -0.274367992 | 0.531814377 | -10.06193462 | 2.70896E-23 | 6.37771E-22 | 41.10494047 |
| Aldh1a7 | -0.275569528 | 1.609287298 | -11.54730866 | 5.95374E-30 | 1.71771E-28 | 56.30548525 |
| Ahsg | -0.27593192 | 5.372690554 | -50.42414369 | 0 | 0 | 825.0639222 |
| Slc25a30 | -0.276044544 | 0.139438954 | -18.01189768 | 1.42571E-67 | 8.37893E-66 | 142.5178091 |
| Tmem50a | -0.276314097 | 1.074433009 | -9.734595482 | 6.17772E-22 | 1.37487E-20 | 38.00930113 |
| Ebpl | -0.276845537 | 1.623453712 | -11.24380079 | 1.58608E-28 | 4.38464E-27 | 53.04818397 |
| Proz | -0.277587392 | 2.120123183 | -14.61989053 | 3.54724E-46 | 1.48909E-44 | 93.4439355 |
| Atpif1 | -0.277717555 | 0.473719223 | -10.84334514 | 1.0726E-26 | 2.80397E-25 | 48.86842442 |
| Fbxo21 | -0.278070442 | 0.371562477 | -12.05123825 | 2.16535E-32 | 6.75045E-31 | 61.88188865 |
| Cebpa | -0.278518049 | 1.172034466 | -9.989793036 | 5.43917E-23 | 1.26738E-21 | 40.41469342 |
| Sucnr1 | -0.278685378 | 0.350391442 | -11.84865668 | 2.12281E-31 | 6.42837E-30 | 59.61509308 |
| Higd1a | -0.279533524 | 1.543251241 | -11.06030586 | 1.11203E-27 | 3.00096E-26 | 51.11620446 |
| Selenof | -0.279983224 | 1.447594249 | -10.79547481 | 1.75921E-26 | 4.56129E-25 | 48.37783126 |
| F7 | -0.280421061 | 1.287337764 | -10.33964081 | 1.77587E-24 | 4.34869E-23 | 43.80402654 |
| Fis1 | -0.280941475 | 1.282703927 | -10.38632689 | 1.116E-24 | 2.75837E-23 | 44.26430194 |
| Rpl35a | -0.280996622 | 2.985827222 | -20.83844009 | 7.79231E-88 | 6.15166E-86 | 189.0416244 |
| Hnrnpa2b1 | -0.281163231 | 1.819068899 | -12.97949666 | 4.07587E-37 | 1.41523E-35 | 72.69269519 |
| Rps9 | -0.281332641 | 2.536475222 | -17.24204302 | 2.02254E-62 | 1.09535E-60 | 130.6950828 |
| Amacr | -0.281671409 | 1.180895838 | -9.852091411 | 2.03228E-22 | 4.61349E-21 | 39.10972259 |
| Stra6l | -0.281872274 | 0.885093569 | -9.843978502 | 2.19528E-22 | 4.97639E-21 | 39.03335306 |
| Stt3a | -0.282127369 | 0.983464776 | -9.833979632 | 2.41408E-22 | 5.45678E-21 | 38.93930929 |
| Rps15 | -0.282175243 | 2.823582324 | -20.05841783 | 4.83796E-82 | 3.55411E-80 | 175.7371526 |
| Ganab | -0.282590246 | 1.096554316 | -9.929137002 | 9.74011E-23 | 2.24645E-21 | 39.83783585 |
| Cnpy2 | -0.284343422 | 1.255340373 | -10.28731744 | 2.98237E-24 | 7.25832E-23 | 43.29039999 |
| Rhou | -0.284538461 | 0.826115931 | -9.892610676 | 1.38125E-22 | 3.15818E-21 | 39.49200245 |
| Rpl9 | -0.284942512 | 2.805104104 | -19.45615714 | 1.13558E-77 | 7.5712E-76 | 165.7011321 |
| Slc46a3 | -0.285269861 | 0.781118924 | -10.81849614 | 1.38702E-26 | 3.60808E-25 | 48.61352017 |
| Acox2 | -0.28634254 | 1.311957339 | -10.45716862 | 5.49539E-25 | 1.37324E-23 | 44.96629926 |
| Eps8l2 | -0.286575027 | 0.602523893 | -10.82834388 | 1.25275E-26 | 3.26415E-25 | 48.71447692 |
| Rps18 | -0.28680249 | 2.6129948 | -18.46744215 | 1.06793E-70 | 6.56816E-69 | 149.6917772 |
| Rpl4 | -0.28741144 | 2.727771799 | -19.63846141 | 5.51906E-79 | 3.80767E-77 | 168.7168041 |
| Coq8a | -0.287881904 | 1.584663503 | -11.76462606 | 5.41861E-31 | 1.61016E-29 | 58.68469178 |
| Furin | -0.288285725 | 0.945545714 | -10.06375502 | 2.66157E-23 | 6.27546E-22 | 41.12241631 |
| Rsrp1 | -0.290363625 | 1.577516304 | -11.49625912 | 1.0396E-29 | 2.97233E-28 | 55.7522547 |
| Rarres2 | -0.290426466 | 2.704391322 | -20.07074757 | 3.92864E-82 | 2.89952E-80 | 175.9447973 |
| Dap | -0.290553107 | 1.462036391 | -11.44916265 | 1.7353E-29 | 4.91711E-28 | 55.24378144 |
| Prkcsh | -0.290617047 | 1.327565878 | -10.86662633 | 8.42625E-27 | 2.21747E-25 | 49.10772029 |
| Habp4 | -0.290823458 | 0.910926361 | -10.02518444 | 3.86599E-23 | 9.06138E-22 | 40.75275249 |
| Sdhb | -0.292786935 | 1.703362533 | -12.56722047 | 5.56716E-35 | 1.83658E-33 | 67.80597092 |
| Id2 | -0.293348103 | 1.474128118 | -11.12949565 | 5.35332E-28 | 1.45706E-26 | 51.84137167 |
| Rpl23 | -0.294676024 | 3.215251744 | -23.88606453 | 8.7426E-112 | 8.7803E-110 | 244.0661909 |
| Slc39a4 | -0.294751715 | 0.608053492 | -10.86183409 | 8.85572E-27 | 2.32269E-25 | 49.0584257 |
| Gstm6 | -0.295494702 | 0.502312138 | -11.29348418 | 9.31598E-29 | 2.60257E-27 | 53.57614124 |
| Clec2d | -0.296251421 | 0.665055891 | -10.83161148 | 1.21112E-26 | 3.16085E-25 | 48.74799379 |
| Gstm1 | -0.296419371 | 2.787285841 | -17.0766701 | 2.45899E-61 | 1.30937E-59 | 128.205923 |
| Psmd13 | -0.296725135 | 0.812173213 | -10.3948474 | 1.02508E-24 | 2.5376E-23 | 44.34850714 |
| Hes6 | -0.296753332 | 1.286792524 | -11.0708231 | 9.95328E-28 | 2.69061E-26 | 51.22617472 |
| Lpcat3 | -0.297228758 | 0.725655157 | -10.41506175 | 8.37708E-25 | 2.08024E-23 | 44.54852766 |
| Rpl17 | -0.298164861 | 2.725193309 | -20.71097441 | 7.05174E-87 | 5.43189E-85 | 186.8444274 |
| Ide | -0.298367561 | 1.062230956 | -10.67818145 | 5.86518E-26 | 1.49148E-24 | 47.18398487 |
| Gpd1 | -0.298853825 | 1.944182012 | -13.96656153 | 1.83876E-42 | 7.1689E-41 | 84.93317768 |
| Pcbd1 | -0.298919053 | 2.250509306 | -15.64933086 | 2.63931E-52 | 1.25391E-50 | 107.492128 |
| Ndufb3 | -0.29928668 | 1.083867555 | -10.56604773 | 1.83457E-25 | 4.6208E-24 | 46.05360106 |
| Tmem238 | -0.299642228 | 0.829989576 | -10.45994314 | 5.34456E-25 | 1.33766E-23 | 44.99388049 |
| H2-T22 | -0.300363641 | 0.658824615 | -10.86189241 | 8.85036E-27 | 2.32269E-25 | 49.05902552 |
| Hsd17b6 | -0.300545007 | 0.787802393 | -9.798204342 | 3.38901E-22 | 7.62792E-21 | 38.60354107 |
| Tufm | -0.30078144 | 1.360674433 | -11.59570482 | 3.50297E-30 | 1.01248E-28 | 56.83195404 |
| Derl2 | -0.301100857 | 1.528446039 | -12.16650361 | 5.82126E-33 | 1.84375E-31 | 63.18659223 |
| Rps11 | -0.301952325 | 2.52591819 | -18.54279044 | 3.20759E-71 | 2.01178E-69 | 150.8908607 |
| Gstp3 | -0.302189998 | 0.248031906 | -14.85183376 | 1.57815E-47 | 6.76811E-46 | 96.54184258 |
| Klhl24 | -0.302559675 | 0.350808843 | -13.03022194 | 2.20535E-37 | 7.72505E-36 | 73.30324409 |
| Serpina1e | -0.302641038 | 5.944339754 | -48.36734602 | 0 | 0 | 778.0370735 |
| Slc23a1 | -0.303311445 | 0.474905629 | -11.83522055 | 2.4669E-31 | 7.42785E-30 | 59.46593663 |
| Idh1 | -0.303336594 | 2.251096885 | -16.15374792 | 1.99216E-55 | 9.90957E-54 | 114.6519485 |
| Ndst1 | -0.303823911 | 0.509400953 | -11.73774538 | 7.3038E-31 | 2.15421E-29 | 58.38828958 |
| G6pc | -0.305552834 | 1.281747458 | -10.9185765 | 4.90975E-27 | 1.31601E-25 | 49.64334357 |
| Echdc1 | -0.306053471 | 0.543402832 | -11.53797513 | 6.59355E-30 | 1.89198E-28 | 56.20417527 |
| Slc7a2 | -0.306419639 | 1.373567568 | -11.53832441 | 6.56842E-30 | 1.88818E-28 | 56.20796512 |
| H2-Q10 | -0.3068391 | 4.382161814 | -40.58346658 | 1.591E-266 | 4.855E-264 | 599.8714974 |
| Sephs2 | -0.307385851 | 2.844145954 | -20.13194852 | 1.39596E-82 | 1.0351E-80 | 176.976761 |
| Fermt2 | -0.307451106 | 0.533950134 | -11.77795309 | 4.67206E-31 | 1.39093E-29 | 58.83186384 |
| Cd164 | -0.307485258 | 1.433803257 | -11.48613595 | 1.16081E-29 | 3.30103E-28 | 55.64280531 |
| Mup14 | -0.307522361 | 1.553590462 | -12.3177273 | 1.02209E-33 | 3.27647E-32 | 64.91465593 |
| Eif4g1 | -0.308024769 | 1.450710001 | -11.97305613 | 5.24617E-32 | 1.61643E-30 | 61.00309253 |
| Cers2 | -0.308222402 | 1.756827185 | -13.52183105 | 5.1679E-40 | 1.92498E-38 | 79.32480337 |
| Mvk | -0.308395385 | 0.495472112 | -12.1877776 | 4.5626E-33 | 1.44799E-31 | 63.42857498 |
| Cpb2 | -0.308877269 | 2.131283266 | -15.98887662 | 2.12953E-54 | 1.03973E-52 | 112.2921243 |
| Foxa2 | -0.30893463 | 0.351008768 | -13.67996282 | 7.08109E-41 | 2.69455E-39 | 81.30157904 |
| Tfpi2 | -0.309944259 | 0.592666557 | -11.49497221 | 1.05429E-29 | 3.00889E-28 | 55.73833619 |
| Tkt | -0.310302893 | 0.758734266 | -10.90861711 | 5.44636E-27 | 1.45493E-25 | 49.5404822 |
| Pex11g | -0.311259077 | 0.892951325 | -10.79678864 | 1.73552E-26 | 4.50725E-25 | 48.39126997 |
| Lamtor2 | -0.311776089 | 1.092317056 | -10.73525712 | 3.26912E-26 | 8.42117E-25 | 47.76345797 |
| Hibadh | -0.312543843 | 1.515042617 | -12.13163924 | 8.67102E-33 | 2.72078E-31 | 62.79081877 |
| Gpx1 | -0.312817925 | 4.57113704 | -37.99666355 | 4.8135E-241 | 1.2946E-238 | 541.2676373 |
| Gpcpd1 | -0.313244076 | 0.354974342 | -13.66820238 | 8.21456E-41 | 3.11839E-39 | 81.15390134 |
| Sult2a8 | -0.313689809 | 2.549185818 | -18.39560962 | 3.35053E-70 | 2.03702E-68 | 148.5519224 |
| F10 | -0.313888146 | 2.602046911 | -19.57991781 | 1.46063E-78 | 9.8627E-77 | 167.7462553 |
| Sema4g | -0.31432802 | 1.405148947 | -12.13158862 | 8.67603E-33 | 2.72078E-31 | 62.79024479 |
| Rpn1 | -0.314775476 | 1.513532388 | -12.29026672 | 1.40371E-33 | 4.4817E-32 | 64.59948379 |
| Cyp4v3 | -0.315316675 | 2.147751813 | -15.75171915 | 6.22282E-53 | 2.99223E-51 | 108.9309903 |
| B2m | -0.315731894 | 3.855270135 | -33.76454674 | 4.1188E-200 | 8.1696E-198 | 447.1322322 |
| Rpl26 | -0.316437482 | 2.635272846 | -20.91634386 | 2.01888E-88 | 1.60178E-86 | 190.3888611 |
| Pdcd4 | -0.316576572 | 0.832660559 | -11.12033889 | 5.89848E-28 | 1.60269E-26 | 51.74517021 |
| Ptprf | -0.317054051 | 1.150244075 | -11.38686976 | 3.40769E-29 | 9.58745E-28 | 54.57407264 |
| Paox | -0.317446148 | 0.495618954 | -12.09349351 | 1.33944E-32 | 4.18391E-31 | 62.35892712 |
| Pnp | -0.318619263 | 0.954087383 | -10.79469976 | 1.77333E-26 | 4.5904E-25 | 48.36990423 |
| Atg101 | -0.318766843 | 0.720601811 | -11.13936553 | 4.82161E-28 | 1.31685E-26 | 51.94514402 |
| Lrg1 | -0.319935236 | 3.247337287 | -26.33531819 | 2.299E-132 | 2.7848E-130 | 291.3662957 |
| Rpl21 | -0.32145586 | 3.040417036 | -23.07331269 | 3.3222E-105 | 3.0124E-103 | 228.9466592 |
| Rps13 | -0.321837408 | 2.546957972 | -20.46236563 | 5.0463E-85 | 3.84975E-83 | 182.5847574 |
| Tmem176b | -0.322942145 | 1.45305851 | -12.57287213 | 5.209E-35 | 1.7256E-33 | 67.8720453 |
| Tprkb | -0.322973796 | 0.809577636 | -11.250564 | 1.47543E-28 | 4.09302E-27 | 53.11993147 |
| Rdh11 | -0.323638963 | 0.389187505 | -13.37940955 | 3.04535E-39 | 1.1186E-37 | 77.56095763 |
| Cyp4f14 | -0.326389375 | 1.512146606 | -12.96357017 | 4.94073E-37 | 1.71178E-35 | 72.50141624 |
| Me1 | -0.327049939 | 1.196363064 | -11.81470918 | 3.10188E-31 | 9.28692E-30 | 59.23852247 |
| Edem2 | -0.328687774 | 0.681241363 | -11.9542124 | 6.48859E-32 | 1.99166E-30 | 60.79202811 |
| Rplp1 | -0.32875955 | 3.73204519 | -34.64674932 | 1.5248E-208 | 3.226E-206 | 466.5221559 |
| Rps14 | -0.329418872 | 2.530034472 | -20.83468444 | 8.31587E-88 | 6.53248E-86 | 188.9767591 |
| Fgb | -0.329562919 | 4.667572122 | -52.97908179 | 0 | 0 | 883.2198258 |
| Gpr146 | -0.330125592 | 0.689261278 | -11.76374324 | 5.47205E-31 | 1.623E-29 | 58.6749479 |
| Slc10a1 | -0.33204738 | 2.493636454 | -19.67850366 | 2.83314E-79 | 1.97176E-77 | 169.3817849 |
| Rpl8 | -0.332164122 | 2.904212876 | -25.48826813 | 3.978E-125 | 4.4768E-123 | 274.7291713 |
| Cdk5rap3 | -0.332578034 | 1.181087399 | -12.44721032 | 2.27121E-34 | 7.4311E-33 | 66.40895836 |
| Cyp2u1 | -0.332688691 | 0.52635008 | -12.47161229 | 1.70805E-34 | 5.61147E-33 | 66.69208062 |
| Kmo | -0.332871987 | 1.298499053 | -12.4125898 | 3.40005E-34 | 1.10557E-32 | 66.00809717 |
| Rpl12 | -0.333490088 | 2.269672726 | -18.50577753 | 5.79383E-71 | 3.59127E-69 | 150.3014024 |
| Tapbp | -0.333499384 | 0.999848805 | -11.75490517 | 6.0368E-31 | 1.78716E-29 | 58.57743491 |
| Rpl6 | -0.334147534 | 2.410687604 | -19.52670759 | 3.53151E-78 | 2.37449E-76 | 166.865864 |
| Ssr4 | -0.334592236 | 1.57644913 | -13.21679722 | 2.26389E-38 | 8.18301E-37 | 75.56633102 |
| Apoc3 | -0.334689183 | 4.670909581 | -51.3507327 | 0 | 0 | 846.1942876 |
| 4931406C07Rik | -0.335300477 | 0.990445666 | -11.82331008 | 2.81794E-31 | 8.45276E-30 | 59.33384053 |
| Kyat1 | -0.335450921 | 1.29386646 | -12.37542743 | 5.23739E-34 | 1.69261E-32 | 65.57887785 |
| Pgd | -0.335966783 | 0.497837966 | -13.0393883 | 1.97325E-37 | 6.92733E-36 | 73.41379003 |
| Cope | -0.336638582 | 1.103068873 | -12.21219027 | 3.44828E-33 | 1.09654E-31 | 63.70671112 |
| Lman1 | -0.336806631 | 1.339472347 | -12.60588244 | 3.53068E-35 | 1.17453E-33 | 68.25848358 |
| Sigmar1 | -0.336911891 | 0.890662656 | -11.805302 | 3.44507E-31 | 1.02756E-29 | 59.1343385 |
| Ces2e | -0.337044623 | 1.575068306 | -13.14475429 | 5.47003E-38 | 1.95933E-36 | 74.68924227 |
| Gys2 | -0.337221741 | 1.129767674 | -12.16236093 | 6.10382E-33 | 1.92556E-31 | 63.13951365 |
| Acat2 | -0.338597911 | 0.715449115 | -12.03895605 | 2.48912E-32 | 7.72941E-31 | 61.74350215 |
| Ddost | -0.338670375 | 1.314568687 | -12.57867651 | 4.86501E-35 | 1.61502E-33 | 67.93993163 |
| Elovl6 | -0.338859549 | 0.521750117 | -12.70250058 | 1.12554E-35 | 3.79194E-34 | 69.3945539 |
| Rcl1 | -0.33994468 | 1.190051575 | -12.44319334 | 2.38019E-34 | 7.77135E-33 | 66.36239745 |
| Smdt1 | -0.340030341 | 1.564411071 | -13.75686054 | 2.67505E-41 | 1.03028E-39 | 82.26981283 |
| H13 | -0.340065898 | 1.521177372 | -13.70541243 | 5.13334E-41 | 1.95807E-39 | 81.62151762 |
| Rpl15 | -0.3415182 | 2.115602578 | -16.96956715 | 1.22793E-60 | 6.51667E-59 | 126.6035172 |
| Acaca | -0.341910118 | 0.547357827 | -12.8421149 | 2.12941E-36 | 7.31373E-35 | 71.04931568 |
| Rpl14 | -0.342931539 | 2.535486708 | -21.22203637 | 9.76724E-91 | 7.90748E-89 | 195.7070724 |
| Rpsa | -0.34301427 | 3.454351676 | -33.60559791 | 1.3403E-198 | 2.5623E-196 | 443.6542058 |
| Nme1 | -0.343067376 | 1.549049191 | -13.64493023 | 1.10167E-40 | 4.17216E-39 | 80.86198257 |
| Fam25c | -0.345499201 | 1.306896067 | -12.77185321 | 4.93171E-36 | 1.67932E-34 | 70.21461188 |
| Cmtm6 | -0.345958832 | 0.697979965 | -12.39381614 | 4.22992E-34 | 1.3726E-32 | 65.79112544 |
| Actb | -0.346810123 | 2.822368113 | -22.55004393 | 4.8543E-101 | 4.23234E-99 | 219.3776116 |
| Serpinf2 | -0.347953982 | 3.054152971 | -26.47868669 | 1.3323E-133 | 1.6262E-131 | 294.2096262 |
| Rps8 | -0.348573846 | 3.215096146 | -27.61057696 | 1.7562E-143 | 2.3031E-141 | 316.9216466 |
| Rps24 | -0.34890752 | 3.279576857 | -29.75780799 | 9.8178E-163 | 1.4837E-160 | 361.18465 |
| Tars | -0.349912203 | 1.307037789 | -12.76818817 | 5.15202E-36 | 1.75058E-34 | 70.17117908 |
| Rps26 | -0.351507513 | 2.75875548 | -23.36324676 | 1.5468E-107 | 1.4698E-105 | 234.3050649 |
| Uox | -0.351842296 | 3.600119174 | -33.57106019 | 2.8546E-198 | 5.3289E-196 | 442.8991233 |
| Rps27a | -0.352082989 | 3.207682387 | -27.96254501 | 1.3573E-146 | 1.8893E-144 | 324.0756666 |
| Rnase4 | -0.352908804 | 2.868544623 | -18.49038278 | 7.40736E-71 | 4.57354E-69 | 150.0564791 |
| Ifitm2 | -0.35311312 | 1.540310676 | -13.99351249 | 1.30026E-42 | 5.08192E-41 | 85.27790458 |
| Rpl32 | -0.353302999 | 2.985501928 | -24.76535274 | 4.7667E-119 | 5.1107E-117 | 260.7585232 |
| Tle5 | -0.353574513 | 2.338788417 | -19.94947527 | 3.03361E-81 | 2.18806E-79 | 173.906226 |
| Rpl30 | -0.353727512 | 2.814326813 | -24.81244846 | 1.9279E-119 | 2.0811E-117 | 261.6620296 |
| Acacb | -0.354178203 | 0.348728416 | -15.4300515 | 5.68283E-51 | 2.61377E-49 | 104.4356406 |
| Sqle | -0.354500604 | 1.030754633 | -12.84278678 | 2.11233E-36 | 7.27083E-35 | 71.05731646 |
| Rpl29 | -0.354571091 | 2.300373677 | -19.88494873 | 8.97039E-81 | 6.41181E-79 | 172.8249769 |
| Erg28 | -0.354758433 | 0.760073561 | -12.27377385 | 1.69788E-33 | 5.41002E-32 | 64.41048377 |
| Tram1 | -0.355240208 | 1.299268481 | -12.80227035 | 3.43009E-36 | 1.17051E-34 | 70.57548452 |
| Tecr | -0.355393525 | 1.262599164 | -12.83035308 | 2.45151E-36 | 8.40185E-35 | 70.90931321 |
| Rps10 | -0.355678117 | 2.739668806 | -24.53000446 | 4.3313E-117 | 4.5516E-115 | 256.2576704 |
| Scap | -0.355907853 | 0.823726954 | -12.38205452 | 4.84943E-34 | 1.57042E-32 | 65.6553381 |
| Rpl36al | -0.356756666 | 2.596652147 | -22.57487017 | 3.0891E-101 | 2.70821E-99 | 219.8286024 |
| Rpl13 | -0.357012229 | 3.303541924 | -29.93554605 | 2.3507E-164 | 3.6214E-162 | 364.9113093 |
| H2-Q7 | -0.357062208 | 1.080560687 | -12.36034303 | 6.23934E-34 | 2.01231E-32 | 65.4049728 |
| Habp2 | -0.357525288 | 1.133637924 | -12.73041992 | 8.07779E-36 | 2.73301E-34 | 69.72422513 |
| Cideb | -0.359543008 | 1.230094064 | -12.96297062 | 4.97663E-37 | 1.72046E-35 | 72.49421948 |
| Slc29a1 | -0.359898072 | 1.17783162 | -13.03979415 | 1.96355E-37 | 6.90858E-36 | 73.41868612 |
| Qdpr | -0.360281463 | 2.279663937 | -19.80190143 | 3.60814E-80 | 2.56745E-78 | 171.4369092 |
| Tst | -0.360450396 | 1.515006505 | -13.84604679 | 8.60114E-42 | 3.32885E-40 | 83.39844636 |
| Atp1a1 | -0.36064196 | 1.741719694 | -15.66281793 | 2.18281E-52 | 1.04014E-50 | 107.6812374 |
| Ces1g | -0.361139532 | 1.730688483 | -14.88358942 | 1.02742E-47 | 4.45441E-46 | 96.96906007 |
| Tmem176a | -0.361615827 | 1.650538134 | -15.09925944 | 5.4627E-49 | 2.41454E-47 | 99.8900177 |
| Npc1 | -0.362559438 | 0.583682468 | -13.28353252 | 9.96293E-39 | 3.62596E-37 | 76.38242204 |
| Rps12 | -0.362637543 | 3.02090214 | -28.34831896 | 5.0272E-150 | 7.1866E-148 | 331.9642933 |
| Nr1d1 | -0.362692816 | 0.380263389 | -16.43795272 | 3.21017E-57 | 1.64319E-55 | 118.7639733 |
| Ido2 | -0.362795059 | 0.719117133 | -13.04283953 | 1.8923E-37 | 6.68754E-36 | 73.45542892 |
| Egfr | -0.363910852 | 1.945468085 | -17.14455538 | 8.83832E-62 | 4.73806E-60 | 129.225528 |
| Aox3 | -0.365252023 | 2.332610155 | -18.68154194 | 3.46976E-72 | 2.18485E-70 | 153.1081058 |
| Slc33a1 | -0.36525483 | 0.849190133 | -13.01868469 | 2.53643E-37 | 8.84574E-36 | 73.16419925 |
| Orm1 | -0.365472454 | 3.995013708 | -38.56086368 | 1.4054E-246 | 3.9124E-244 | 553.9967927 |
| Mpeg1 | -0.368448414 | 0.644538249 | -13.38262615 | 2.92627E-39 | 1.07736E-37 | 77.60062048 |
| Rbp1 | -0.368681268 | 0.753692245 | -12.8234178 | 2.66369E-36 | 9.10934E-35 | 70.82681304 |
| Taldo1 | -0.369616497 | 1.460337519 | -14.68816938 | 1.42477E-46 | 6.02888E-45 | 94.35176969 |
| Mup20 | -0.369911041 | 6.27435199 | -34.01376633 | 1.7345E-202 | 3.5287E-200 | 452.5952727 |
| Xbp1 | -0.375016217 | 1.500507733 | -14.50083925 | 1.72602E-45 | 7.07713E-44 | 91.86929306 |
| Vmp1 | -0.375354714 | 1.280860334 | -14.00295708 | 1.15143E-42 | 4.51133E-41 | 85.39883972 |
| Tuba1b | -0.376348145 | 0.791096837 | -13.1742207 | 3.81501E-38 | 1.37271E-36 | 75.04749084 |
| Rpl28 | -0.377238778 | 3.010339451 | -27.94088953 | 2.1118E-146 | 2.914E-144 | 323.6342962 |
| Haao | -0.377951193 | 2.164857355 | -18.17933566 | 1.02752E-68 | 6.12957E-67 | 145.1394872 |
| Gjb1 | -0.377954755 | 2.448091008 | -21.25651502 | 5.33664E-91 | 4.34266E-89 | 196.3100499 |
| Slc22a30 | -0.378482827 | 1.589438075 | -14.8813432 | 1.05912E-47 | 4.57932E-46 | 96.93881682 |
| Aadat | -0.378756644 | 0.950405496 | -13.24475654 | 1.6058E-38 | 5.83087E-37 | 75.90781603 |
| Rarres1 | -0.380850419 | 0.513504958 | -14.43493424 | 4.12562E-45 | 1.66579E-43 | 91.00212517 |
| Ppib | -0.381790982 | 2.450537867 | -21.67989164 | 3.03211E-94 | 2.53229E-92 | 203.765424 |
| Ptms | -0.381996453 | 2.105607079 | -17.91291762 | 6.69338E-67 | 3.8622E-65 | 140.9763797 |
| Rps16 | -0.382532506 | 2.908520003 | -27.75559579 | 9.2173E-145 | 1.2291E-142 | 319.8641787 |
| Slc8b1 | -0.383988889 | 0.422094537 | -15.85077427 | 1.52712E-53 | 7.38788E-52 | 110.3300535 |
| Sec61a1 | -0.386070977 | 1.635323805 | -15.95619368 | 3.39849E-54 | 1.65421E-52 | 111.8265795 |
| Eif4ebp3 | -0.387470099 | 0.758209575 | -13.54703428 | 3.76957E-40 | 1.41074E-38 | 79.6385725 |
| Cyb5r3 | -0.387496514 | 2.052332253 | -18.84255078 | 2.58871E-73 | 1.6497E-71 | 155.6958251 |
| Acsl5 | -0.388489542 | 1.688395422 | -16.73566444 | 4.00799E-59 | 2.09897E-57 | 123.1307003 |
| Dgat2 | -0.388509995 | 2.172142526 | -19.88680381 | 8.69539E-81 | 6.24337E-79 | 172.8560284 |
| Aspg | -0.389133717 | 1.411965005 | -14.33793232 | 1.47896E-44 | 5.89651E-43 | 89.73168484 |
| Hhex | -0.390584699 | 1.105766207 | -14.17370159 | 1.26406E-43 | 5.00202E-42 | 87.59682137 |
| Grb7 | -0.390767367 | 0.699430587 | -14.14216077 | 1.90424E-43 | 7.51653E-42 | 87.18913973 |
| Rpl19 | -0.39169387 | 2.721749889 | -26.48984382 | 1.0671E-133 | 1.3126E-131 | 294.4312225 |
| Etnk2 | -0.39332738 | 1.845395955 | -17.89050114 | 9.49238E-67 | 5.45743E-65 | 140.6281518 |
| C6 | -0.394440258 | 1.320858812 | -14.56437374 | 7.42839E-46 | 3.0938E-44 | 92.70832885 |
| Fads1 | -0.39568861 | 1.404089769 | -14.82468245 | 2.27649E-47 | 9.71057E-46 | 96.17715512 |
| Uba52 | -0.395974389 | 1.920469909 | -17.87538261 | 1.20124E-66 | 6.88131E-65 | 140.3934751 |
| Saa4 | -0.397058452 | 2.030123611 | -18.93376475 | 5.90838E-74 | 3.78041E-72 | 157.1688162 |
| Rpl11 | -0.397400851 | 2.90605599 | -28.07822402 | 1.2764E-147 | 1.8083E-145 | 326.4360212 |
| Khk | -0.397664439 | 2.441163511 | -22.94961301 | 3.2442E-104 | 2.9084E-102 | 226.6726354 |
| Cyp2f2 | -0.397973323 | 3.479223399 | -27.88336034 | 6.829E-146 | 9.2618E-144 | 322.462529 |
| Apcs | -0.398565497 | 1.851622659 | -17.41404945 | 1.47658E-63 | 8.07944E-62 | 133.3032149 |
| Qsox1 | -0.398870756 | 2.144908798 | -19.79144519 | 4.29803E-80 | 3.04469E-78 | 171.2624239 |
| 2200002D01Rik | -0.402430414 | 0.647568823 | -14.49178836 | 1.94581E-45 | 7.93732E-44 | 91.75001122 |
| Lactb2 | -0.402510852 | 1.45269904 | -15.61826093 | 4.08576E-52 | 1.93531E-50 | 107.0569706 |
| Slc25a1 | -0.402555757 | 1.715680477 | -17.5615355 | 1.54167E-64 | 8.5836E-63 | 135.5549546 |
| C8b | -0.402834505 | 2.362961525 | -23.98165885 | 1.4426E-112 | 1.458E-110 | 245.864417 |
| Qprt | -0.405624136 | 1.431320104 | -15.39148922 | 9.71551E-51 | 4.43005E-49 | 103.9016781 |
| C9 | -0.40585328 | 2.571944946 | -26.07400564 | 4.0495E-130 | 4.8679E-128 | 286.2039657 |
| Rps2 | -0.408741413 | 3.387198231 | -33.32837741 | 5.7521E-196 | 1.0613E-193 | 437.6001292 |
| Fga | -0.409891763 | 4.035539356 | -48.67629708 | 0 | 0 | 785.1097361 |
| Nat8f1 | -0.411451435 | 2.18817012 | -21.46540532 | 1.35222E-92 | 1.10603E-90 | 199.9766933 |
| Rpl18 | -0.412936192 | 2.512902558 | -24.69363099 | 1.8885E-118 | 1.9978E-116 | 259.3843905 |
| Tmem97 | -0.413261072 | 0.888787321 | -14.40749328 | 5.92449E-45 | 2.37999E-43 | 90.64201607 |
| Srd5a1 | -0.41500385 | 0.885148462 | -14.48886156 | 2.02269E-45 | 8.22974E-44 | 91.71145196 |
| Dbp | -0.415235157 | 0.520901525 | -15.52688287 | 1.47135E-51 | 6.90753E-50 | 105.7811284 |
| F11 | -0.415297341 | 0.76477701 | -15.14757767 | 2.81776E-49 | 1.25244E-47 | 100.5490623 |
| Gm10076 | -0.415519688 | 1.448099287 | -16.46945321 | 2.02462E-57 | 1.04308E-55 | 119.2231555 |
| Srebf1 | -0.417333142 | 0.916417462 | -14.53219178 | 1.139E-45 | 4.69444E-44 | 92.28295946 |
| Tmbim6 | -0.417985448 | 2.706002451 | -25.84778361 | 3.4869E-128 | 4.0387E-126 | 281.7561629 |
| Cyp2d40 | -0.419148103 | 1.258387724 | -15.06472288 | 8.75908E-49 | 3.86081E-47 | 99.41998865 |
| Ifi27 | -0.419999339 | 1.473497066 | -14.47059308 | 2.57573E-45 | 1.04531E-43 | 91.47091689 |
| 1810008I18Rik | -0.420717018 | 0.457044661 | -16.28798058 | 2.85473E-56 | 1.44725E-54 | 116.5871625 |
| Tomm6 | -0.420772485 | 1.633271416 | -17.86490205 | 1.41409E-66 | 8.04257E-65 | 140.2308766 |
| Oaf | -0.420953509 | 2.183713285 | -22.34445433 | 2.02574E-99 | 1.74698E-97 | 215.654586 |
| Alas2 | -0.423014223 | 0.929596008 | -14.83778553 | 1.90768E-47 | 8.15931E-46 | 96.3530839 |
| C8a | -0.424658833 | 2.506770503 | -25.57748374 | 6.9676E-126 | 7.8973E-124 | 276.4681424 |
| Mcm10 | -0.425182428 | 0.585330172 | -16.14048048 | 2.41228E-55 | 1.19619E-53 | 114.4613508 |
| Rpl10a | -0.433375429 | 2.528413524 | -26.50079341 | 8.5815E-134 | 1.0638E-131 | 294.6487419 |
| Fdx1 | -0.434649432 | 1.115546418 | -15.46758089 | 3.36869E-51 | 1.55843E-49 | 104.9563228 |
| Dhrs3 | -0.434888738 | 1.243594708 | -15.93087164 | 4.87917E-54 | 2.36766E-52 | 111.466399 |
| Tm7sf2 | -0.435774787 | 0.998148289 | -15.5061731 | 1.96549E-51 | 9.17308E-50 | 105.4928 |
| Irf7 | -0.43650189 | 0.692684145 | -15.30698864 | 3.13477E-50 | 1.41717E-48 | 102.735349 |
| Slc26a1 | -0.436531631 | 1.072745042 | -15.51414587 | 1.75823E-51 | 8.22995E-50 | 105.6037633 |
| Hsd17b12 | -0.437788599 | 1.53111434 | -17.40559034 | 1.68016E-63 | 9.13043E-62 | 133.174496 |
| Angptl4 | -0.438235524 | 1.33901663 | -16.05506024 | 8.24544E-55 | 4.06331E-53 | 113.2371458 |
| Rps5 | -0.440284363 | 2.388743118 | -24.7234378 | 1.066E-118 | 1.1352E-116 | 259.9551997 |
| Tmem205 | -0.440403622 | 2.784837391 | -31.00471198 | 3.4833E-174 | 5.8182E-172 | 387.5118485 |
| Lsr | -0.440579499 | 1.380775075 | -16.76327781 | 2.66102E-59 | 1.39818E-57 | 123.5387684 |
| Mup10 | -0.441057042 | 1.200792807 | -15.71179797 | 1.09404E-52 | 5.22896E-51 | 108.3690963 |
| H2-T23 | -0.453015291 | 1.257841123 | -16.77303182 | 2.3023E-59 | 1.21372E-57 | 123.6830351 |
| Rpl27a | -0.453103566 | 3.005123413 | -35.95726431 | 3.5611E-221 | 8.4339E-219 | 495.5718 |
| Depp1 | -0.454787344 | 0.284219125 | -22.07735639 | 2.50163E-97 | 2.13418E-95 | 210.8491935 |
| Cyb5b | -0.45481565 | 1.523077622 | -17.9807247 | 2.32189E-67 | 1.35955E-65 | 142.0316756 |
| Adipor2 | -0.457504235 | 1.639776858 | -19.08213766 | 5.28613E-75 | 3.45187E-73 | 159.5756064 |
| Apol9b | -0.457840964 | 1.15667819 | -16.63885349 | 1.67803E-58 | 8.75888E-57 | 121.7041069 |
| Lgals9 | -0.46594871 | 2.073084959 | -23.26211916 | 1.011E-106 | 9.382E-105 | 232.431584 |
| Tmem258 | -0.467515073 | 0.981706547 | -16.48909028 | 1.51846E-57 | 7.87416E-56 | 119.509748 |
| Rpl27 | -0.468258981 | 2.20996731 | -25.48051354 | 4.6277E-125 | 5.1713E-123 | 274.5781719 |
| Cish | -0.470192497 | 0.1960698 | -27.71906601 | 1.9378E-144 | 2.5625E-142 | 319.122288 |
| Pdhb | -0.47048405 | 1.281380241 | -17.10558914 | 1.59078E-61 | 8.49915E-60 | 128.6398996 |
| Slco1a1 | -0.47077006 | 1.818581797 | -19.78952327 | 4.43847E-80 | 3.1302E-78 | 171.2303593 |
| Ces3a | -0.471620522 | 3.671562034 | -50.59104821 | 0 | 0 | 828.8730071 |
| Pqlc1 | -0.471674721 | 0.95508733 | -16.48611908 | 1.58604E-57 | 8.19783E-56 | 119.4663681 |
| Mettl7a1 | -0.472754689 | 1.398696175 | -18.26841834 | 2.51747E-69 | 1.50744E-67 | 146.5415119 |
| Txn1 | -0.474724549 | 1.692403066 | -19.65479861 | 4.20492E-79 | 2.9137E-77 | 168.9880028 |
| Fdft1 | -0.476528034 | 1.429166714 | -18.32428574 | 1.03943E-69 | 6.29529E-68 | 147.4233182 |
| Bri3 | -0.478264964 | 1.166138373 | -17.71367638 | 1.47695E-65 | 8.34027E-64 | 137.8925564 |
| Rpl7a | -0.478589223 | 2.236792518 | -24.49908067 | 7.8195E-117 | 8.1632E-115 | 255.668046 |
| Fxyd1 | -0.478990471 | 1.722540129 | -19.630032 | 6.35005E-79 | 4.36202E-77 | 168.5769361 |
| Gcat | -0.481002823 | 1.276358351 | -17.41779369 | 1.39451E-63 | 7.65677E-62 | 133.3602045 |
| Fgg | -0.485066796 | 4.161120906 | -61.31528174 | 0 | 0 | 1069.683416 |
| AW112010 | -0.485738919 | 1.455044331 | -17.61091741 | 7.21233E-65 | 4.044E-63 | 136.312052 |
| Sec61b | -0.485754234 | 1.702577481 | -20.76504104 | 2.77329E-87 | 2.15719E-85 | 187.7753179 |
| Ulk1 | -0.489547387 | 0.535124199 | -18.80755798 | 4.55668E-73 | 2.89222E-71 | 155.1320778 |
| H6pd | -0.492555374 | 1.389886711 | -19.17788668 | 1.10556E-75 | 7.2492E-74 | 161.1358022 |
| Mlxipl | -0.49449427 | 0.933375377 | -16.84831996 | 7.51335E-60 | 3.97406E-58 | 124.798741 |
| Apol9a | -0.496178315 | 0.769563315 | -17.4601613 | 7.2966E-64 | 4.02023E-62 | 134.0057031 |
| Pklr | -0.496374691 | 0.424396794 | -20.7310684 | 4.98593E-87 | 3.85936E-85 | 187.1902078 |
| Fkbp11 | -0.496421931 | 0.69233109 | -18.10501965 | 3.30909E-68 | 1.96662E-66 | 143.973689 |
| Ang | -0.496666537 | 2.062836167 | -21.08242331 | 1.12178E-89 | 8.94496E-88 | 193.2719522 |
| Krtcap2 | -0.501367552 | 1.223895943 | -18.3238834 | 1.04608E-69 | 6.31148E-68 | 147.4169607 |
| Cyp4a12a | -0.503852301 | 0.944992503 | -17.58050534 | 1.15169E-64 | 6.43489E-63 | 135.8456031 |
| Pmvk | -0.504330571 | 0.941560952 | -18.78702072 | 6.34776E-73 | 4.013E-71 | 154.8015621 |
| Apoa1 | -0.505390456 | 5.424205436 | -71.72664005 | 0 | 0 | 1292.623648 |
| Abcb11 | -0.506326669 | 1.896688191 | -23.21569599 | 2.3898E-106 | 2.1794E-104 | 231.57316 |
| Dbi | -0.509892434 | 3.675673787 | -49.02203618 | 0 | 0 | 793.0213558 |
| Slc22a1 | -0.511909909 | 1.737147869 | -21.19078157 | 1.68847E-90 | 1.36003E-88 | 195.1610226 |
| Dio1 | -0.512225225 | 1.510015277 | -20.00526817 | 1.18578E-81 | 8.6312E-80 | 174.8430533 |
| Rpl22l1 | -0.513464248 | 1.508087674 | -19.61321028 | 8.40003E-79 | 5.74533E-77 | 168.2979397 |
| Akr1c14 | -0.53108738 | 1.557448268 | -20.22025444 | 3.1251E-83 | 2.32813E-81 | 178.4695106 |
| Lect2 | -0.532370177 | 0.768165577 | -18.29918296 | 1.5471E-69 | 9.29899E-68 | 147.0268564 |
| Tlcd2 | -0.535572949 | 1.183059485 | -19.01703929 | 1.52686E-74 | 9.88904E-73 | 158.5179962 |
| Cyp2c67 | -0.535582345 | 1.212745694 | -18.9544781 | 4.22146E-74 | 2.72302E-72 | 157.5040137 |
| Gne | -0.536340629 | 1.028784952 | -19.23832669 | 4.10586E-76 | 2.71466E-74 | 162.1234743 |
| Ttr | -0.536974677 | 5.440219604 | -89.37892587 | 0 | 0 | 1640.403826 |
| Sec61g | -0.542194836 | 2.110113188 | -27.37112632 | 2.2447E-141 | 2.9196E-139 | 312.0788803 |
| AI182371 | -0.543295059 | 2.392100751 | -30.44843656 | 4.7059E-169 | 7.3934E-167 | 375.7146493 |
| Atp5g2 | -0.551318751 | 1.58446837 | -22.86043529 | 1.6697E-103 | 1.4884E-101 | 225.0377916 |
| Erbb3 | -0.5557211 | 0.445609932 | -22.95807847 | 2.7764E-104 | 2.5032E-102 | 226.8280264 |
| Mup17 | -0.561122091 | 3.450683013 | -29.43702331 | 8.0784E-160 | 1.2093E-157 | 354.4817379 |
| Glul | -0.568776818 | 0.92298748 | -18.42742525 | 2.01997E-70 | 1.23281E-68 | 149.0563843 |
| Gm47283 | -0.571300035 | 1.293475188 | -20.45751794 | 5.48265E-85 | 4.16262E-83 | 182.5020377 |
| Pygl | -0.575470322 | 1.851440143 | -25.95472642 | 4.2536E-129 | 4.9997E-127 | 283.8563031 |
| Ces3b | -0.579500079 | 2.374445652 | -31.86223704 | 3.684E-182 | 6.4239E-180 | 405.8513871 |
| Nsdhl | -0.582875385 | 0.573094137 | -22.04242888 | 4.6837E-97 | 3.97438E-95 | 210.2234669 |
| Cyp51 | -0.595118943 | 1.517027117 | -22.82730154 | 3.066E-103 | 2.7179E-101 | 224.4313457 |
| Pnpla7 | -0.601912052 | 1.416199728 | -23.34301893 | 2.2526E-107 | 2.1277E-105 | 233.9299428 |
| Slc38a3 | -0.607363671 | 3.009925132 | -44.73394689 | 8.861E-308 | 3.1246E-305 | 694.7551228 |
| Aqp8 | -0.608345011 | 0.900604667 | -21.48832853 | 9.02144E-93 | 7.41721E-91 | 200.380465 |
| Enho | -0.610336036 | 1.013967376 | -21.57457653 | 1.96277E-93 | 1.63064E-91 | 201.9021142 |
| Acads | -0.616955788 | 1.535700728 | -24.84674297 | 9.967E-120 | 1.0833E-117 | 262.3205422 |
| Lpin1 | -0.617681114 | 0.886367995 | -21.76119624 | 7.14238E-95 | 6.02847E-93 | 205.2078611 |
| Cela1 | -0.618070979 | 0.689484875 | -23.60541056 | 1.693E-109 | 1.6481E-107 | 238.8107162 |
| Slc2a2 | -0.623790944 | 1.477216936 | -23.9985224 | 1.0493E-112 | 1.0673E-110 | 246.182063 |
| Akr1c6 | -0.633148238 | 3.14437736 | -45.77397985 | 3.8013E-318 | 1.4028E-315 | 718.6002738 |
| Rgn | -0.639497682 | 2.084157254 | -23.30609188 | 4.472E-107 | 4.1989E-105 | 233.2456299 |
| Lpin2 | -0.642688545 | 0.917280641 | -22.69016601 | 3.7716E-102 | 3.32489E-100 | 221.9270134 |
| Hsd3b3 | -0.651452857 | 1.379742952 | -24.49057925 | 9.1977E-117 | 9.5392E-115 | 255.5060216 |
| Hsd3b7 | -0.663505037 | 1.863396285 | -29.10480037 | 8.1795E-157 | 1.213E-154 | 347.5718263 |
| Msmo1 | -0.670298606 | 1.788959802 | -28.39965125 | 1.7505E-150 | 2.5251E-148 | 333.017642 |
| Lifr | -0.670970859 | 1.36751429 | -25.5905425 | 5.3979E-126 | 6.1621E-124 | 276.7229491 |
| Slc22a7 | -0.674006539 | 1.005869488 | -24.19027798 | 2.788E-114 | 2.8542E-112 | 249.8029382 |
| Hsd3b5 | -0.678623791 | 1.172863092 | -23.82926662 | 2.5453E-111 | 2.5243E-109 | 242.9997149 |
| Gamt | -0.679725129 | 1.776687815 | -30.49503451 | 1.7548E-169 | 2.8126E-167 | 376.6997339 |
| Acss2 | -0.695495735 | 1.292912245 | -25.85547877 | 2.9975E-128 | 3.4974E-126 | 281.9071306 |
| Fabp1 | -0.715279533 | 5.128772177 | -78.26874969 | 0 | 0 | 1426.058899 |
| Saa2 | -0.731287854 | 1.933639852 | -30.0971905 | 7.8304E-166 | 1.2182E-163 | 368.3082773 |
| Hsd17b2 | -0.731411975 | 1.369609418 | -27.84602224 | 1.4619E-145 | 1.9659E-143 | 321.7026135 |
| Ttc39c | -0.734312408 | 1.89330462 | -34.41035862 | 2.8086E-206 | 5.788E-204 | 461.312654 |
| Mup7 | -0.739098732 | 4.881190147 | -71.71093942 | 0 | 0 | 1292.297036 |
| Foxq1 | -0.771430036 | 0.403077686 | -35.28116618 | 1.2112E-214 | 2.7069E-212 | 480.5505289 |
| Hmgcs1 | -0.771850952 | 1.84928021 | -31.21697144 | 3.7595E-176 | 6.4147E-174 | 392.0344264 |
| Ces2c | -0.773826732 | 0.388247337 | -36.97063765 | 5.1005E-231 | 1.2646E-228 | 518.2111388 |
| Cyp2c29 | -0.775400073 | 1.984513219 | -23.62360373 | 1.2047E-109 | 1.18E-107 | 239.1503082 |
| Keg1 | -0.788918413 | 1.136615294 | -27.89277395 | 5.6362E-146 | 7.71E-144 | 322.6541915 |
| Ubc | -0.791538204 | 2.994893215 | -57.52658043 | 0 | 0 | 985.672654 |
| Arrdc3 | -0.792851088 | 0.507522776 | -34.93848387 | 2.4086E-211 | 5.2357E-209 | 472.9646793 |
| Mup18 | -0.804378853 | 1.119886959 | -29.0491186 | 2.6004E-156 | 3.8206E-154 | 346.4169526 |
| Car3 | -0.805383948 | 3.722750416 | -68.64504893 | 0 | 0 | 1227.945965 |
| Slc25a47 | -0.806874042 | 2.688947642 | -51.1883809 | 0 | 0 | 842.4948917 |
| Gulo | -0.813174284 | 1.364210867 | -27.21844919 | 4.8958E-140 | 6.1656E-138 | 309.0015196 |
| Dhcr7 | -0.815790505 | 0.973769735 | -30.80158961 | 2.6272E-172 | 4.2978E-170 | 383.1947401 |
| Sc5d | -0.851589249 | 2.129420599 | -42.96637982 | 3.5537E-290 | 1.1748E-287 | 654.268069 |
| Osgin1 | -0.854708514 | 1.500576219 | -33.59007978 | 1.8825E-198 | 3.5561E-196 | 443.314911 |
| Acly | -0.861543655 | 1.862623209 | -37.32980056 | 1.6015E-234 | 4.0988E-232 | 526.2677328 |
| Mup12 | -0.870603394 | 2.092548429 | -35.72632961 | 6.1096E-219 | 1.405E-216 | 490.4330915 |
| Cyp7b1 | -0.88331914 | 2.104994602 | -39.42884139 | 4.0413E-255 | 1.166E-252 | 573.6410002 |
| Comt | -0.909251013 | 2.254488943 | -45.77258898 | 3.9246E-318 | 1.4153E-315 | 718.5683815 |
| Idi1 | -0.910615088 | 1.367505114 | -34.69495342 | 5.2572E-209 | 1.1273E-206 | 467.5856645 |
| Tkfc | -0.916399371 | 1.853881402 | -40.92497104 | 6.6138E-270 | 2.1418E-267 | 607.6481698 |
| Bst2 | -0.93708962 | 1.670998173 | -38.35951621 | 1.3324E-244 | 3.6452E-242 | 549.4502796 |
| Serpina12 | -0.989697066 | 1.730156381 | -36.7736871 | 4.2223E-229 | 1.0308E-226 | 513.8002111 |
| Upp2 | -1.005197131 | 1.562911445 | -40.85979029 | 2.9245E-269 | 9.2813E-267 | 606.1633015 |
| Nudt7 | -1.010586068 | 2.708531675 | -65.79208333 | 0 | 0 | 1167.062231 |
| Ifitm3 | -1.013411377 | 2.705642531 | -62.9038121 | 0 | 0 | 1104.484084 |
| Mup1 | -1.016146672 | 2.776231798 | -58.1856571 | 0 | 0 | 1000.383265 |
| Elovl3 | -1.021266954 | 1.285340559 | -46.2387726 | 8.8932E-323 | 3.4417E-320 | 729.257716 |
| Mup11 | -1.047822421 | 4.626821447 | -56.32453573 | 0 | 0 | 958.7465995 |
| Fasn | -1.101144582 | 2.149908213 | -51.43461397 | 0 | 0 | 848.1051133 |
| Fdps | -1.126186833 | 1.978301812 | -51.00826258 | 0 | 0 | 838.38915 |
| Ly6e | -1.161150766 | 2.074773851 | -51.86875319 | 0 | 0 | 857.9890357 |
| Mup16 | -1.200583233 | 2.412989002 | -54.92073748 | 0 | 0 | 927.1533962 |
| Fabp5 | -1.214797766 | 0.864919044 | -46.04165313 | 8.1521E-321 | 3.0799E-318 | 724.7379265 |
| Saa1 | -1.227051007 | 3.020384405 | -52.45208078 | 0 | 0 | 871.2533295 |
| Phlda1 | -1.259711462 | 0.877882943 | -50.24447982 | 0 | 0 | 820.9623016 |
| Cyp2c70 | -1.272061683 | 2.572140299 | -67.88962263 | 0 | 0 | 1211.91728 |
| Cyp1a2 | -1.30349925 | 2.100251311 | -51.13518848 | 0 | 0 | 841.2825504 |
| Selenbp2 | -1.404956929 | 3.251584828 | -96.30519575 | 0 | 0 | 1766.366657 |
| Thrsp | -2.118645756 | 2.590445485 | -105.6055639 | 0 | 0 | 1926.720153 |

Table S2

|  | logFC | AveExpr | t | P.Value | adj.P.Val | B |
| --- | --- | --- | --- | --- | --- | --- |
| Cyp4a14 | 3.094074799 | 2.191116532 | 170.1243158 | 0 | 0 | 2818.066975 |
| Cyp4a10 | 2.775464904 | 2.100170727 | 148.455683 | 0 | 0 | 2555.114845 |
| Mt1 | 1.520174794 | 1.643217888 | 64.41798417 | 0 | 0 | 1137.405489 |
| Angptl8 | 1.244569841 | 1.221224482 | 48.64931842 | 0 | 0 | 784.492231 |
| Retsat | 1.082467354 | 2.461287263 | 58.96567992 | 0 | 0 | 1017.742809 |
| Tsku | 1.03405412 | 0.837779038 | 37.04964589 | 8.6619E-232 | 2.1817E-229 | 519.9820259 |
| Creld2 | 1.017940127 | 1.848767352 | 43.54685796 | 5.9344E-296 | 2.0035E-293 | 667.5557839 |
| Herpud1 | 1.009577746 | 3.058055425 | 74.64282008 | 0 | 0 | 1352.761086 |
| Tsc22d3 | 0.990925759 | 1.610620216 | 39.64454721 | 3.0154E-257 | 8.8608E-255 | 578.5333674 |
| Gck | 0.96040244 | 0.985429066 | 33.69557806 | 1.8676E-199 | 3.6586E-197 | 445.6224968 |
| G0s2 | 0.959065592 | 0.837897595 | 35.20035869 | 7.2743E-214 | 1.6032E-211 | 478.7599853 |
| Hspa1b | 0.897473744 | 0.785864794 | 35.58045234 | 1.5691E-217 | 3.5569E-215 | 487.19123 |
| Alas1 | 0.889866301 | 1.340039844 | 32.61591096 | 3.122E-189 | 5.5663E-187 | 422.1133103 |
| Mat1a | 0.874092249 | 3.561747584 | 84.01836104 | 0 | 0 | 1538.905713 |
| Etnppl | 0.87375654 | 1.303190661 | 30.47499687 | 2.6821E-169 | 4.256E-167 | 376.2760648 |
| mt-Cytb | 0.84443909 | 4.120578326 | 65.25960913 | 0 | 0 | 1155.595174 |
| Mfsd2a | 0.805252361 | 0.545816501 | 30.98531723 | 5.2657E-174 | 8.7038E-172 | 387.0991787 |
| mt-Co1 | 0.780425146 | 4.372108183 | 64.70913126 | 0 | 0 | 1143.706863 |
| Cyp7a1 | 0.775429496 | 0.428286474 | 32.37241137 | 6.0998E-187 | 1.0755E-184 | 416.845341 |
| Mt2 | 0.742558653 | 0.747778354 | 29.89825537 | 5.1473E-164 | 7.8536E-162 | 364.1286865 |
| Noct | 0.726206578 | 0.609657978 | 28.06622971 | 1.6312E-147 | 2.2907E-145 | 326.1910787 |
| Nr1i3 | 0.64604753 | 1.327587452 | 23.28740558 | 6.3255E-107 | 5.9043E-105 | 232.8995884 |
| Serpina3m | 0.633040113 | 2.095478351 | 31.21156541 | 4.2198E-176 | 7.1234E-174 | 391.9190999 |
| mt-Nd2 | 0.617542067 | 3.528447254 | 38.97158049 | 1.2881E-250 | 3.65E-248 | 563.2834355 |
| Ttpa | 0.616998975 | 2.283558786 | 33.65194789 | 4.8572E-199 | 9.3992E-197 | 444.6679017 |
| Ppp1r3b | 0.612723779 | 1.483694937 | 23.2548054 | 1.1579E-106 | 1.0682E-104 | 232.2962758 |
| Slc25a25 | 0.589849592 | 0.895560131 | 20.23413269 | 2.46908E-83 | 1.84808E-81 | 178.7045138 |
| mt-Nd3 | 0.586227914 | 2.729153184 | 34.41841768 | 2.3515E-206 | 4.9096E-204 | 461.4900938 |
| Hamp | 0.581470266 | 2.580003487 | 23.80778512 | 3.8114E-111 | 3.7565E-109 | 242.596744 |
| mt-Nd4 | 0.579353325 | 3.573992602 | 37.40417389 | 3.0079E-235 | 7.9548E-233 | 527.9380391 |
| Foxa3 | 0.579182237 | 1.021121704 | 19.68990494 | 2.3428E-79 | 1.63769E-77 | 169.5712964 |
| mt-Co3 | 0.571641695 | 4.732237944 | 49.92463275 | 0 | 0 | 813.6570803 |
| Sult1a1 | 0.570257334 | 2.410910816 | 32.71913033 | 3.3238E-190 | 5.9934E-188 | 424.3503195 |
| Hsph1 | 0.561347963 | 1.028371216 | 20.63472182 | 2.62258E-86 | 2.01039E-84 | 185.5342812 |
| mt-Atp6 | 0.558549823 | 4.317479805 | 43.65558664 | 4.9064E-297 | 1.6925E-294 | 670.0457768 |
| Tat | 0.548261204 | 3.029386579 | 40.7222685 | 6.7267E-268 | 2.0929E-265 | 603.0313437 |
| Hspa5 | 0.537151085 | 3.39532622 | 47.54417897 | 0 | 0 | 759.1816036 |
| Acsl1 | 0.53280699 | 2.620175762 | 33.1788724 | 1.5025E-194 | 2.7405E-192 | 434.3415813 |
| Dnaja1 | 0.527539426 | 2.04314966 | 25.7065881 | 5.5691E-127 | 6.4036E-125 | 278.9902609 |
| Ddit3 | 0.527014028 | 0.380966154 | 23.24459373 | 1.3991E-106 | 1.2833E-104 | 232.1073971 |
| Gm29966 | 0.526510968 | 0.518449194 | 20.24030152 | 2.22349E-83 | 1.67215E-81 | 178.8090066 |
| Hacl1 | 0.503650741 | 1.615428372 | 20.40710455 | 1.29783E-84 | 9.80665E-83 | 181.6425733 |
| Hsp90aa1 | 0.49497007 | 2.31525429 | 27.24447507 | 2.8965E-140 | 3.677E-138 | 309.5255161 |
| Bnip3 | 0.473325015 | 1.389902656 | 17.68620903 | 2.25811E-65 | 1.27063E-63 | 137.4694217 |
| St3gal5 | 0.469869233 | 1.583395634 | 18.53545116 | 3.60683E-71 | 2.25328E-69 | 150.7739099 |
| mt-Co2 | 0.464936902 | 3.806656628 | 28.64932919 | 1.0219E-152 | 1.4877E-150 | 338.1531228 |
| Bag3 | 0.453092008 | 0.666705939 | 16.37459201 | 8.09644E-57 | 4.13101E-55 | 117.8424231 |
| Ehhadh | 0.450898013 | 1.397870512 | 15.99038968 | 2.08391E-54 | 1.0206E-52 | 112.3136948 |
| mt-Nd5 | 0.450611829 | 1.073260805 | 15.13450879 | 3.37084E-49 | 1.49409E-47 | 100.37064 |
| Tmem37 | 0.444912489 | 1.511717374 | 17.86621655 | 1.38545E-66 | 7.90806E-65 | 140.2512664 |
| mt-Nd1 | 0.443429708 | 3.250439372 | 26.02583557 | 1.0475E-129 | 1.2404E-127 | 285.2552139 |
| Crat | 0.442849894 | 0.361454556 | 19.58072155 | 1.44127E-78 | 9.81547E-77 | 167.7595663 |
| Fkbp5 | 0.439603341 | 0.484964936 | 17.47899175 | 5.46932E-64 | 3.03451E-62 | 134.2929734 |
| Crot | 0.437135308 | 2.302218773 | 23.42286384 | 5.1029E-108 | 4.9075E-106 | 235.4117645 |
| Gpat4 | 0.435782722 | 1.079442514 | 15.41931847 | 6.59833E-51 | 3.01736E-49 | 104.286916 |
| Cth | 0.435221729 | 2.317346821 | 23.41996981 | 5.3854E-108 | 5.1479E-106 | 235.3580032 |
| Paqr9 | 0.434532372 | 1.722087882 | 18.43376766 | 1.82601E-70 | 1.11873E-68 | 149.1570234 |
| Nnmt | 0.432408608 | 2.010314832 | 19.7408502 | 1.00128E-79 | 7.03023E-78 | 170.4190295 |
| Abhd2 | 0.432355443 | 0.576012205 | 16.21068541 | 8.75151E-56 | 4.38072E-54 | 115.4712873 |
| Ephx2 | 0.429191063 | 3.128664756 | 36.09448031 | 1.6685E-222 | 4.0114E-220 | 498.6288467 |
| Scd1 | 0.426473834 | 3.456751748 | 23.8744608 | 1.0877E-111 | 1.0855E-109 | 243.8481931 |
| Sdc1 | 0.420818438 | 1.880514525 | 19.21114441 | 6.41194E-76 | 4.22177E-74 | 161.6790089 |
| Gk | 0.416682165 | 0.654571412 | 15.3357509 | 2.10518E-50 | 9.57164E-49 | 103.1317676 |
| Vnn1 | 0.414513238 | 0.466613969 | 16.24449582 | 5.36401E-56 | 2.7021E-54 | 115.9588853 |
| Dnajb9 | 0.38761497 | 1.002130703 | 15.16535639 | 2.20766E-49 | 9.84022E-48 | 100.7919838 |
| Tmed5 | 0.386680884 | 0.820746364 | 13.59026249 | 2.19169E-40 | 8.24117E-39 | 80.17788661 |
| Cyp3a25 | 0.383919613 | 1.865098694 | 17.21905781 | 2.86516E-62 | 1.54641E-60 | 130.3480321 |
| Abcc3 | 0.381729061 | 1.113568953 | 13.37072847 | 3.39134E-39 | 1.24281E-37 | 77.45395393 |
| Hspa8 | 0.381409295 | 3.37618962 | 34.00017146 | 2.3383E-202 | 4.6968E-200 | 452.2969622 |
| Manf | 0.381017729 | 2.030389713 | 17.95091807 | 3.69928E-67 | 2.15019E-65 | 141.567428 |
| Acot4 | 0.375857776 | 0.282004272 | 18.044854 | 8.5077E-68 | 5.03732E-66 | 143.032424 |
| Acot1 | 0.361688717 | 0.248356009 | 18.0188174 | 1.27931E-67 | 7.54652E-66 | 142.6258037 |
| Atf5 | 0.361323407 | 1.422618083 | 13.08301858 | 1.16122E-37 | 4.113E-36 | 73.9408755 |
| Gm4952 | 0.360284135 | 1.036423251 | 13.52038159 | 5.26245E-40 | 1.95561E-38 | 79.30677312 |
| Dpyd | 0.35842791 | 1.782337161 | 15.71542161 | 1.03946E-52 | 4.98314E-51 | 108.4200528 |
| Usp2 | 0.35829634 | 0.377357149 | 15.99484616 | 1.95511E-54 | 9.60484E-53 | 112.3772366 |
| Tmem56 | 0.355561121 | 1.032137842 | 12.64474843 | 2.23111E-35 | 7.46905E-34 | 68.71458823 |
| Pim3 | 0.354964569 | 0.562927403 | 13.36082845 | 3.83387E-39 | 1.40175E-37 | 77.331997 |
| Hnf4a | 0.353917232 | 1.726673936 | 15.3165292 | 2.74712E-50 | 1.24546E-48 | 102.8667767 |
| Hsd17b13 | 0.350118281 | 2.570068908 | 17.96933787 | 2.77424E-67 | 1.61844E-65 | 141.8542555 |
| Sgk1 | 0.349537891 | 0.335512345 | 15.41936849 | 6.59374E-51 | 3.01736E-49 | 104.2876088 |
| Asl | 0.349401999 | 2.969258181 | 25.32954002 | 8.7559E-124 | 9.716E-122 | 271.643231 |
| Ppp1r3c | 0.346270366 | 0.655046495 | 12.6133161 | 3.23424E-35 | 1.07817E-33 | 68.34562659 |
| Per1 | 0.345334314 | 0.311031596 | 15.50198495 | 2.08395E-51 | 9.69739E-50 | 105.4345283 |
| Acnat2 | 0.342851042 | 0.351868868 | 14.67834614 | 1.62492E-46 | 6.85749E-45 | 94.22094794 |
| Plin5 | 0.336246143 | 0.694458295 | 12.09831598 | 1.26788E-32 | 3.96818E-31 | 62.41346255 |
| Apoa4 | 0.33505028 | 1.357664042 | 11.84412314 | 2.23322E-31 | 6.74984E-30 | 59.564749 |
| Inhbc | 0.332330811 | 1.124612734 | 11.16922919 | 3.51168E-28 | 9.64072E-27 | 52.25962945 |
| Hspb8 | 0.327887614 | 1.2589241 | 12.16298149 | 6.06064E-33 | 1.91574E-31 | 63.14656504 |
| Eci2 | 0.326138098 | 1.415846017 | 12.3589625 | 6.34004E-34 | 2.04064E-32 | 65.38906615 |
| St3gal4 | 0.324513332 | 0.810869117 | 11.42947425 | 2.14861E-29 | 6.0774E-28 | 55.03176331 |
| Cxcl12 | 0.320683897 | 1.407442307 | 11.80844386 | 3.32645E-31 | 9.9405E-30 | 59.16912631 |
| Agxt | 0.317580652 | 2.7658542 | 20.03202471 | 7.55249E-82 | 5.52271E-80 | 175.2929581 |
| Dusp1 | 0.314080923 | 0.343770896 | 13.7362298 | 3.47494E-41 | 1.33189E-39 | 82.00960215 |
| Hsdl2 | 0.311587652 | 0.567000846 | 11.67232077 | 1.50681E-30 | 4.41961E-29 | 57.66936567 |
| Cd1d1 | 0.311288665 | 1.695468612 | 13.08724971 | 1.10293E-37 | 3.91529E-36 | 73.99207027 |
| Htatip2 | 0.306025086 | 0.750333854 | 10.76603303 | 2.38264E-26 | 6.1576E-25 | 48.07706588 |
| Syvn1 | 0.304476502 | 1.071734272 | 11.67180284 | 1.51545E-30 | 4.43676E-29 | 57.66368851 |
| Acaa1b | 0.303983554 | 3.142863102 | 21.73481335 | 1.14224E-94 | 9.58995E-93 | 204.7394234 |
| Cpt1a | 0.300504279 | 1.361894701 | 10.90133473 | 5.87518E-27 | 1.56685E-25 | 49.46532224 |
| Atp2a2 | 0.298049579 | 1.482244692 | 12.04258506 | 2.38874E-32 | 7.43227E-31 | 61.78437831 |
| Lpgat1 | 0.296426328 | 1.313661499 | 11.29311086 | 9.35338E-29 | 2.60843E-27 | 53.57216647 |
| Slc4a4 | 0.295755511 | 0.390856344 | 12.61948169 | 3.00725E-35 | 1.00461E-33 | 68.4179379 |
| Tm4sf4 | 0.294070006 | 0.881857039 | 10.18698452 | 8.00692E-24 | 1.91346E-22 | 42.3120889 |
| Hsp90ab1 | 0.292306602 | 2.872457117 | 21.18400054 | 1.90125E-90 | 1.52369E-88 | 195.0426208 |
| Pik3r1 | 0.291847668 | 0.427417919 | 11.62780947 | 2.46131E-30 | 7.15312E-29 | 57.18226736 |
| Rdh16 | 0.291086534 | 0.478371734 | 11.26834585 | 1.21974E-28 | 3.38964E-27 | 53.3087526 |
| Gnmt | 0.28987917 | 4.41897118 | 35.80011182 | 1.1817E-219 | 2.7575E-217 | 492.0740062 |
| Aldoa | 0.289640542 | 0.835080107 | 9.992448634 | 5.3018E-23 | 1.23719E-21 | 40.44002199 |
| Maob | 0.287065649 | 1.218089988 | 10.27357708 | 3.41601E-24 | 8.27562E-23 | 43.15590985 |
| Slc20a1 | 0.286707031 | 0.41419271 | 11.65654155 | 1.79344E-30 | 5.23129E-29 | 57.49650268 |
| Pcx | 0.283791882 | 2.27476523 | 16.0821088 | 5.59018E-55 | 2.7634E-53 | 113.624245 |
| Ecm1 | 0.283580856 | 1.485332222 | 9.042076021 | 3.39577E-19 | 6.87297E-18 | 31.76994192 |
| Rcan1 | 0.280171872 | 0.345220951 | 12.00952386 | 3.47414E-32 | 1.07461E-30 | 61.41238296 |
| Gm16157 | 0.276306314 | 0.469279838 | 10.61407249 | 1.12718E-25 | 2.85263E-24 | 46.53641081 |
| 5033403H07Rik | 0.275218149 | 0.64475171 | 10.0726931 | 2.44056E-23 | 5.76293E-22 | 41.20826361 |
| Acadm | 0.271911442 | 1.762636074 | 11.869518 | 1.68069E-31 | 5.11885E-30 | 59.84697105 |
| BC005537 | 0.268478737 | 1.325879278 | 9.552064877 | 3.3935E-21 | 7.38656E-20 | 36.32374619 |
| Slc39a14 | 0.266863435 | 1.198421709 | 9.470880556 | 7.17241E-21 | 1.52973E-19 | 35.58345806 |
| Slco1a4 | 0.266307891 | 0.364798604 | 11.2441184 | 1.5807E-28 | 4.37741E-27 | 53.05155241 |
| Ddx3x | 0.266136838 | 0.99370281 | 9.315929796 | 2.94501E-20 | 6.1651E-19 | 34.18664382 |
| Ech1 | 0.264003347 | 1.95201697 | 12.56784363 | 5.5265E-35 | 1.82697E-33 | 67.81325516 |
| Ahcy | 0.262419795 | 2.24295933 | 14.53464931 | 1.10245E-45 | 4.56754E-44 | 92.31541498 |
| Picalm | 0.262238172 | 0.747092827 | 9.439619851 | 9.55327E-21 | 2.02662E-19 | 35.29995095 |
| Chka | 0.260333045 | 0.503539156 | 10.37184032 | 1.2893E-24 | 3.18121E-23 | 44.12127935 |
| Klf15 | 0.260142475 | 1.277766241 | 9.468458754 | 7.33369E-21 | 1.55784E-19 | 35.56146368 |
| Prlr | 0.259008745 | 0.523281059 | 9.790106111 | 3.65894E-22 | 8.2238E-21 | 38.52769032 |
| Mafb | 0.25656964 | 0.668847643 | 9.197993275 | 8.50895E-20 | 1.7467E-18 | 33.13772128 |
| Lrrc58 | 0.255908053 | 0.580557782 | 9.312004823 | 3.05146E-20 | 6.37952E-19 | 34.15153721 |
| Lbp | 0.252893824 | 1.003833392 | 8.897391372 | 1.20337E-18 | 2.36325E-17 | 30.52006394 |
| Eif1a | 0.250653547 | 0.713775569 | 9.015756569 | 4.28041E-19 | 8.57596E-18 | 31.54118862 |

Table S3

| **Table S3-Primers used in this study** | |
| --- | --- |
| **RT-qPCR** | |
| **Oligonucleotides** | **Sequence** |
| Cd36_F | GGAGCCATCTTTGAGCCTTCA |
| Cd36_R | GAACCAAACTGAGGAATGGATCT |
| Plin5_F | TGTCCAGTGCTTACAACTCGG |
| Plin5_R | CAGGGCACAGGTAGTCACAC |
| Ces1e_F | CCAGTGACAGGGCAAATAGTC |
| Ces1e_R | GTAGACAGGACCAGTCCATCATA |
| Mfsd2a_F | AGAAGCAGCAACTGTCCATTT |
| Mfsd2a_F | CTCGGCCCACAAAAAGGATAAT |
| Scd1_F | TTCTTGCGATACACTCTGGTGC |
| Scd1_R | CGGGATTGAATGTTCTTGTCGT |
| G0s2_F | GTGAAGCTATACGTGCTGGG |
| G0s2_R | CCGTCTCAACTAGGCCGAG |
| Acsl1_F | TGCCAGAGCTGATTGACATTC |
| Acsl1_R | GGCATACCAGAAGGTGGTGAG |
| Cyp4a10_F | TTCCCTGATGGACGCTCTTTA |
| Cyp4a10_R | GCAAACCTGGAAGGGTCAAAC |
| Cyp4a14_F | TTTAGCCCTACAAGGTACTTGGA |
| Cyp4a14_R | GCAGCCACTGCCTTCGTAA |
| Cpt1a_F | CTCCGCCTGAGCCATGAAG |
| Cpt1a_R | CACCAGTGATGATGCCATTCT |
| Acot1_F | ATACCCCCTGTGACTATCCTGA |
| Acot1_R | CAAACACTCACTACCCAACTGT |
| Ehhadh_F | ATGGCTGAGTATCTGAGGCTG |
| Ehhadh_R | GGTCCAAACTAGCTTTCTGGAG |
| Ech1_F | GCTACCGCGATGACAGTTTC |
| Ech1_R | TCAGAGATCGAAGGCTGATGTT |
| Acaa1b_F | CAGGACGTGAAGCTAAAGCCT |
| Acaa1b_R | CTCCGAAGTTATCCCCATAGGAA |
| Crot_F | GAACGGACATTTCAGTACCAGG |
| Crot_R | CTTCATTTGCGAATGGTTTCACT |
| Decr1_F | GATCCGGGTCCTCAGAGGTTT |
| Decr1_R | ATCAGGTGGTAGCATAGGCTT |
| Hsd17b10_F | GCTTGGTCGCGGTAGTAACTG |
| Hsd17b10_R | TGGGGCAAATATGCAGCTTTC |
| Hnf4a_F | CACGCGGAGGTCAAGCTAC |
| Hnf4a_R | CCCAGAGATGGGAGAGGTGAT |
| Acly_ F | ACCCTTTCACTGGGGATCACA |
| Acly_ R | GACAGGGATCAGGATTTCCTTG |
| Acss2_ F | AAACACGCTCAGGGAAAATCA |
| Acss2_ R | ACCGTAGATGTATCCCCCAGG |
| Fasn_ F | GGAGGTGGTGATAGCCGGTAT |
| Fasn_ R | TGGGTAATCCATAGAGCCCAG |
| Lpin1_ F | CATGCTTCGGAAAGTCCTTCA |
| Lpin1_ R | GGTTATTCTTTGGCGTCAACCT |
| Thrsp_ F | ATGCAAGTGCTAACGAAACGC |
| Thrsp_ R | CCTGCCATTCCTCCCTTGG |
| Mlxipl_ F | AGATGGAGAACCGACGTATCA |
| Mlxipl_ R | ACTGAGCGTGCTGACAAGTC |
| Fdx1_F | CAAGGGGAAAATTGGCGACTC |
| Fdx1_R | TTGGTCAGACAAACTTGGCAG |
| Cyb5b_F | GAGCCCTCCGTCACCTACTA |
| Cyb5b_R | AGCTTTCAGTTGCATCAGCAC |
| Akr1c6_F | CAGACAGTGCGTCTAAGTGATG |
| Akr1c6_R | CGGATGGCTAGTCCTACTTCCT |
| Hsd17b2_F | ATGAGCCCGTTTGCCTCTG |
| Hsd17b2_R | CCACAGGTAACAAGTCTTGGTC |
| Hsd3b3_F | TGGGGAGAGAAGTCAATTCCTT |
| Hsd3b3_R | TGAACGTACTAGCTGTCAGTGT |
| Hsd3b5_F | AGTGCTAAATAGCGTGTTTACCA |
| Hsd3b5_R | ACTTTTTGTGTAGTGTCTCCCTG |
| Akr1c14_F | GTGTGGTACTAAACGATGGTCAC |
| Akr1c14_R | CAAATAAGCGGAGTCAAAATGGC |
| Comt_F | CTGGGGGTTGGTGGCTATTG |
| Comt_R | CCCACTCCTTCTCTGAGCAG |
| Cyp1a2_F | AGTACATCTCCTTAGCCCCAG |
| Cyp1a2_R | GGTCCGGGTGGATTCTTCAG |
| Cyp2c29_F | ATCTGGTCGTGTTCCTAGCG |
| Cyp2c29_R | AGTAGGCTTTGAGCCCAAATAC |
| Cyp2c40_F | GGCTCACAGCCTATTGTGGTA |
| Cyp2c40_R | TCAAAAACCGGAATCCTTCCTC |
| Cyp2c70_F | AGTATGGCCCTGTGTTTACTGT |
| Cyp2c70_R | GCCTTGGCTGGTTCTACTGAG |
| Cyp4a12a_F | CCTCTAATGGCTGCAAGGCTA |
| Cyp4a12a_R | CCAGGTGATAGAAGTCCCATCT |
| Gstm3_F | CCCCAACTTTGACCGAAGC |
| Gstm3_R | GGTGTCCATAACTTGGTTCTCCA |
| Gstm6_F | TTGGAGAACAGGGTCATGGAC |
| Gstm6_R | GGGTTCAAACATTCGATGCTGA |
| Hmgcs1_F | AATTGGGCCAAACGCTCCT |
| Hmgcs1_R | TCTCGTACAAGAGAACTGGCTA |
| Pmvk_F | CTTGGAGGTAACATCTGTGCTC |
| Pmvk_R | GTGCTCGCATCCAGAAGTCTC |
| Fdps_F | CCGAGCCACCCTCTCATTTG |
| Fdps_R | TCAGTCCTGAATGCCTCACCT |
| Fdft1_F | ATGGAGTTCGTCAAGTGTCTAGG |
| Fdft1_R | CGTGCCGTATGTCCCCATC |
| Cyp51_F | GACAGGAGGCAACTTGCTTTC |
| Cyp51_R | GTGGACTTTTCGCTCCAGC |
| Msmo1_F | AAACAAAAGTGTTGGCGTGTTC |
| Msmo1_R | AAGCATTCTTAAAGGGCTCCTG |
| Nsdhl_F | TCATGGTGAATCAAAGCGAGG |
| Nsdhl_R | CCGGGGGTTATCAAAGCCTTG |
| Dhcr7_F | AGGCTGGATCTCAAGGACAAT |
| Dhcr7_R | GCCAGACTAGCATGGCCTG |
| Manf_F | TCTGGGACGATTTTACCAGGA |
| Manf_R | TCTTGCTTCACGGCAAAACTTTA |
| Dnajb9_F | CTCCACAGTCAGTTTTCGTCTT |
| Dnajb9_R | GGCCTTTTTGATTTGTCGCTC |
| Hsp90ab1_F | TCAAACAAGGAGATTTTCCTCCG |
| Hsp90ab1_R | GCTGTCCAACTTAGAAGGGTC |
| Pdia4_F | ACTCTCCGGGAATTTGTCACA |
| Pdia4_R | ATGTCGTTGGCGAGTAGCATC |
| Hsp90aa1_F | TGTTGCGGTACTACACATCTGC |
| Hsp90aa1_R | GTCCTTGGTCTCACCTGTGATA |
| Hspb8_F | TCCCGTGCTCCTACCCAAG |
| Hspb8_R | GCTGTCAAGTCGTCTGGAAAAG |
| Hspa8_F | TCTCGGCACCACCTACTCC |
| Hspa8_R | CTACGCCCGATCAGACGTTT |
| Ddit3_F | CAAGGCAAGAGCTGCCATAG |
| Ddit3_R | CCGGTACTTAGCGTCAGGG |
| Atf4_F | ATGGCGCTCTTCACGAAATC |
| Atf4_R | ACTGGTCGAAGGGGTCATCAA |
| Ces1b_F | GCTCTTTGCCCAATATGGGGA |
| Ces1b_R | ACTGCCACAGGGTGTTCAAAT |
| Ces1c_F | CTCAGATATGTTCAGCACCGAAA |
| Ces1c_R | GCTTTTTGTCAAATCGGCAGG |
| Ces2b_F | AACGATGAGTTTGGTTGGACC |
| Ces2b_R | GAGGCAGCATCAGTTGTGC |
| Ces2c_F | GCCAACCCCATCAGAAACACA |
| Ces2c_R | TTCAGCATGTCAAGATTTTGCAG |
| Ces1d_F | ATGCGCCTCTACCCTCTGATA |
| Ces1d_R | AGCAAATCTCAAGGAGCCAAG |
| Ces1e_F | CCAGTGACAGGGCAAATAGTC |
| Ces1e_R | GTAGACAGGACCAGTCCATCATA |
| Ces1f_F | ATGCAGCTAGAGGACAGGC |
| Ces1f_R | GAAAAGTCCGCAGGAGTGTAA |
| Ces1g_F | CTGCTTGCTTGAGTCTGGGAC |
| Ces1g_R | TTGGTAGCACAAAGGAGGGTA |
| Ces2e_F | TGACCGAGTCACCATTTTTGG |
| Ces2e_R | GGAGATAAGATCAGGGAGCACA |
| Ces2g_F | AGGTCCAAGGCAGGCTCAT |
| Ces2g_R | GGCCCTCCATATTCATCGTAACA |
| Ces2h_F | AACTGTCTACGAGGCAAAAGCG |
| Ces2h_R | GAGGATGTCTGGGCAGGAAGAT |
| Hmgcs2_F | GAAGAGAGCGATGCAGGAAAC |
| Hmgcs2_R | GTCCACATATTGGGCTGGAAA |
| Hmgcl_F | CAGGTGAAGATCGTGGAAGTC |
| Hmgcl_R | GGAGCCCTGCTTCGGAAAC |
| Bdh1_F | ACAAGACACACGCTGTTGTTT |
| Bdh1_R | CTCTTCAAGCTGTCCAGTTCC |
| Acat1_F | CAGGAAGTAAGATGCCTGGAAC |
| Acat1_R | TTCACCCCCTTGGATGACATT |
| Nr1i3_F | CTCCCCTGGTGAGGATCATC |
| Nr1i3_R | GACCGAGAGTTGGGTAGAGGT |
| Cyp2f2_F | GCACCCAAACCTCTCCCAATC |
| Cyp2f2_R | CCGTGACACCGACCCATAC |
| Cyp2e1_F | CGTTGCCTTGCTTGTCTGGA |
| Cyp2e1_R | AACAAACGAATTCGCAAACCTCC |
| Ppara_F | AGAGCCCCATCTGTCCTCTC |
| Ppara_R | ACTGGTAGTCTGCAAAACCAAA |
| Ppard_F | GAGCACACCCTTCCTTCCAG |
| Ppard_R | CTCGTACTTGAGCTTCATGCG |
| Acot1_F | ATACCCCCTGTGACTATCCTGA |
| Acot1_R | CAAACACTCACTACCCAACTGT |
| Acot2_F | GTTGTGCCAACAGGATTGGAA |
| Acot2_R | GCTCAGCGTCGCATTTGTC |
| Ces2a_F | GTTGTGCCAACAGGATTGGAA |
| Ces2a_R | GCTCAGCGTCGCATTTGTC |
| Hspa5_F | ACTTGGGGACCACCTATTCCT |
| Hspa5_R | ACTTGGGGACCACCTATTCCT |
|  |  |
| **ChiP-qPCR** | |
| ChIP_Acot1_F | CACCGGAGTCACCTGATAGAGTC |
| ChIP_Acot1_R | GCCAGGGTGCACAGACTTT |
| ChIP_Cyp4a10_F | GAAGGAAAAGGCCACCGTCT |
| ChIP_Cyp4a10_R | AACTCCTAGGGCCAAAGGTC |
| ChIP_Nr1i3_F | AGGTATCAGGGTTGGAGCCT |
| ChIP_Nr1i3_R | AAGACTCGGTAGGCAAAGGC |
